# The collection-based inventory and spatial analysis of *Hieracium* s.str. (Asteraceae) in Finland

**DOI:** 10.3897/BDJ.13.e154676

**Published:** 2025-07-09

**Authors:** Alexander Sennikov

**Affiliations:** 1 University of Helsinki, Helsinki, Finland University of Helsinki Helsinki Finland

**Keywords:** apomictic taxa, checklist, distribution pattern, endemic, hawkweeds

## Abstract

**Background:**

Due to the lack of complete taxonomic inventories and revisions for nearly 120 years and a high number of chaotic and conflicting species descriptions in the times of the primary biodiversity exploration, the actual diversity of apomictic species of *Hieracium* in Finland is currently unknown. The existing nomenclatural and bibliographic inventory, published in 2002, does not include taxonomic evaluations. To compensate for this deficiency, there is an urgent need for a new taxonomic revision. The operative foundation for this revision is an inventory of the taxonomic diversity and distribution patterns on the basis of primary sources, i.e. herbarium collections identified by major taxonomic authorities.

**New information:**

A complete checklist of *Hieracium* species occurring in Finland, which were accepted in major authoritative sources (recently or in the past) and identified in herbarium collections, includes 137 accepted species, which are deemed mostly apomictic. These taxa are considered taxonomically evaluated and are recommended for current use in national checklists and manuals. Brief species descriptions are provided as the basis for a new identification key. Species distributions in Finland are recorded on the basis of herbarium specimens according to the traditional biogeographic provinces of Eastern Fennoscandia. The distribution patterns revealed in the hierarchical cluster analysis highlight a largely isolated position of the Åland Islands, with strong phytogeographical connections to Sweden; the presence of taxa with strictly oceanic distributions in south-western Finland; and a high level of taxonomic dissimilarity between the north (Lapland and neighbouring territories) and the east (southern and central mainland Finland and neighbouring territories) of Finland. The eastern floristic element is considered autochthonously developed in Finland during the postglacial colonisation of Northern Europe by *Hieracium* plants and contains numerous endemics or near-endemics of the country. Two species are established aliens, which were introduced as park ornamentals over 100 years ago. A new synonymy is established: Hieraciumschmidtiisubsp.residuum is reduced to a synonym of *H.crinellum*; its only record in Finland is a local relic of the postglacial period, remotely isolated from the main populations in Norway and southern Sweden. Numerous further province-level and country-level records are expected in Finland in the course of a new taxonomic revision, with a total to reach or exceed 200 species.

## Introduction

Human impact on the global biosystems is constantly and aggressively changing the world. For this reason, biodiversity conservation is currently considered the highest challenge that will supersede climate change risk in global financial priorities (Karolyi and Tobin‐de la Puente 2023). The task of cataloguing the biodiversity data in an efficient and sustainable way requires an unprecedented effort in completing biodiversity databases across countries (Chapin III et al. 2000, Ceballos et al. 2015) and harmonising the effort at the global scale (Feng et al. 2022). Much work is still required to complete the coverage and to level the discrepancies between the exising global plant diversity databases (Schellenberger Costa et al. 2023). In particular, territorial coverage remains incomplete or insufficiently detailed in many areas and much effort has been recently used to aggregate the information and fill the gaps in large-scale regional data compilations, for example, for Northern Asia (Chepinoga et al. 2024) and Central Asia (Ma et al. 2024).

With the development of the national biodiversity information facility (FINBIF 2025), Finland may be considered at the top of the global effort to catalogue and provide the biodiversity data. A national checklist for vascular plants has been published in a book version (Kurtto et al. 2019) and has been regularly updated at the annual basis online (the latest update: Kurtto et al. (2025)). It covers all native and alien plants recorded in the country, as well as most commonly cultivated plants, except for the major apomictic groups (*Hieracium* L., *Pilosella* Hill, *Ranunculusauricomus*-group, *Taraxacum* F.H.Wigg.), for which a selection of taxa is provided.

The taxonomy of *Hieracium* in Finland has early roots (Sennikov 2002a). Johan Petter Norrlin (1842-1917) was the leading authority in the genus; his most complete monograph (Norrlin 1906) covered the whole of Eastern Fennoscandia, including Finland, the Kola Peninsula (now Murmansk Region, Russia) and Russian Karelia. However, he did not live to produce the updated version of that treatment and his sad competition with another greatest authority, Mårten Magnus Wilhelm Brenner (1843-1930), led to the confusing situation when numerous taxa were described locally without subsequent taxonomic evaluation (Sennikov 2002a). This nomenclatural disorder and the long-standing lack of taxonomic expertise in the country accounted for the loss of the actual knowledge. While the Russian parts of Eastern Fennoscandia had been covered by a comprehensve revision (Schljakov 1989), only a selection of taxa appeared in more recent Finnish national treatments (Fagerström 1984, Hackman 1986, Hackman and Sennikov 1998). Although this selection had increased with every next edition, the resulting treatment was far from complete.

As the first step towards a new comprehensive revision, the bibliography of the Finnish *Hieracium* species was compiled, their nomenclature was established and the Finnish *Hieracium* literature was indexed to facilitate informed taxonomic revisions and correct synonymisations (Sennikov 2002a). This work became a cornerstone for the nomenclatural backbone of the European *Hieracium* checklist (Greuter 2006). Meanwhile Torbjörn Tyler (Lund) initiated a large-scale taxonomic revision of *Hieracium* in Sweden, producing a number of detailed regional treatments (Tyler 1997, Tyler 1998, Tyler 2002a, Tyler 2002b, Tyler 2002c, Tyler 2002d, Tyler 2003a, Tyler 2003b, Tyler 2003c, Tyler 2004a, Tyler 2005, Tyler 2006a, Tyler 2008, Tyler 2010a, Tyler 2010c). This revision paralleled the monograph of *Hieracium* in Denmark (Schou 2001). In appreciation of the process, frankly but aptly, Nordenstam (2003) called it "Renascence of Scandinavian hieraciology".

Despite these sincere hopes, a new taxonomic revision of the Finnish *Hieracium* species did not take place in time, largely because of my ongoing involvement in *Atlas Florae Europeae* (Kurtto et al. 2010, Kurtto et al. 2013, Kurtto et al. 2018) and large-scale projects in Central Asia (e.g. Sennikov et al. (2016), Sennikov and Lazkov (2024)). Nomenclatural contributions, dealing with typifications of classical plant names, appeared from Finland and Sweden (Tyler 2000a, Sennikov 2003a, Sennikov 2005, Tyler 2006b, Tyler 2006c, Tyler 2006d, Tyler 2007, Sennikov 2008a, Tyler 2009, Tyler and Sennikov 2015), whereas significant taxonomic revisions came from Sweden, but also affected Finland because of the overlapping species distributions (Tyler 2004b, Tyler 2006e, Tyler 2011, Tyler 2017).

There has also been significant progress in the understanding of evolutionary processes in Swedish *Hieracium* species, with significant implications for classification of the Finnish taxa. Ploidy levels in several apomictic *Hieracium* species have been determined, showing the likelihood of interlineage crosses in the postglacial evolution that led to the formation of the current *Hieracium* diversity in Fennoscandia (Tyler and Jönsson 2009). A study on the variability of plastid and nuclear markers (Tyler and Jönsson 2013) has demonstrated the heterogeneity of the traditional taxonomic groups and the need for smaller supraspecific entities in order to achieve more natural infrageneric classifications.

The preparation of a new national manual of vascular plants (Finnish-language paper book) has long been announced in Finland (Uotila 2012). As part of this work, an updated overview of the *Hieracium* species occurring in Finland has been prepared and is provided here for the international audience. It can also be considered the second major step towards a new taxonomic revision of the genus in Finland.

The aim of this study is to collect the information on the taxonomic and spatial diversity of *Hieracium* in Finland. The synopsis provided in this work summarises the published taxonomic knowledge by listing the species-level taxa which have been accepted by previous authoritative taxonomic treatments in the country, i.e. which have been accepted and evaluated with certainty after their formal description and for which some material exists in addition to their type collections.

## Materials and methods

Finland is situated in Northern Europe, with the geographical area totalling 337,030 km^2^. Its territory is predominantly lowland or hilly (Fig. 1a), with a small portion of the Scandinavian Mountains in the extreme north-west. The territory stretches latitudinally through several biomes, from the northern limit of the broadleaved forest in the south through all types of taiga forest to the tundra fells in the extreme north (Hämet-Ahti 1988), which is determined by a high variation in regional temperatures (Fig. 1c, d). The country is broadly open to the Baltic Sea, with a high influence of the oceanic climate in the south-west, but also with a proximity to the extensive continental landmass in the south-east, both strongly affecting plant distributions (Ahti et al. 1968).

The taxonomic inventory of *Hieracium* in Finland is based exclusively on the herbarium material kept at the Botanical Museum, Finnish Museum of Natural History, University of Helsinki (H). Mostly, the published evidence is taken into account, largely based on the latest presentation in Hackman and Sennikov (1998) with the additional information from Norrlin (1906).

Species delimitation largely follows the major synopses (Norrlin 1906, Hackman and Sennikov 1998). Unresolved taxonomic and nomenclatural conflicts are treated conservatively, pending the future studies.

The species grouping is formal and employed for convenience rather than for natural classification, although smaller and more natural groups are preferred and adopted as much as currently possible. Informal group names are used instead of the sectional nomenclature because of the lack of taxonomic updates for Finland and the conflicting taxonomic treatments published for the neighbouring countries.

The taxonomic nomenclature follows the earlier inventory of the bibliography of *Hieracium* taxa published by the Finnish authors (Sennikov 2002a, Sennikov 2006a); nomenclatural and bibliographic references can be found in these sources and are not reproduced here. Recent nomenclatural updates and corrections (Greuter 2006, POWO 2025) have been implemented. Synonyms are limited to the most important names featuring in the recent literature.

Morphological descriptions are based on personal observations in herbarium collections of H. As this inventory is focused on the taxonomic diversity and distribution patterns, the species descriptions are brief and limited to the most essential characters, providing the basis for the identification keys. The descriptions do not necessarily reflect the complete species variation ranges. The quantitative terminology in the descriptions of the leaves and synflorescences follows Schljakov (1989). Fine-scale measurements are made exclusively on dried material, using a stereomicroscope with a ruler.

Species distributions are provided according to the traditional biogeographic provinces of Eastern Fennoscandia (Fig. 1b). The reason to recognise these provinces comes from their current practical use in national manuals (e.g. Hämet-Ahti et al. (1998)) and herbarium collections (e.g. Uotila (2013)). Frequency of occurrence is estimated for each province-level record according to the number of herbarium specimens available from a province and formalised as three-step grading system.

Species distributions are analysed using the IBM SPSS Statistics software (IBM 2021). A province-level species occurrence dataset (Suppl. material 1) has been subjected to the hierarchical cluster analysis (Nearest Neighbour cluster method, Pearson correlation measure) to uncover a major similarity pattern in the *Hieracium* species distributions in Finland. Two territories are excluded from the analysis due to their much smaller size and the poor availability of their *Hieracium* data, i.e. the Finnish fragments of *Kareliaaustralis* and *Karelialadogensis* (their major parts were ceded to the USSR in 1940).

The floristic elements used in the analysis follow Lahti et al. (1988). These authors established that, besides the common taxa occurring in the whole country, the following main types of specific plant distributions can be determined in Finland: south-western or southern (limited to the southernmost provinces adjacent to the Gulf of Finland, more abundant in the west), oceanic (following the coastal areas of the Gulf of Bothnia and the Gulf of Finland), eastern/continental (south-east of the country) and northern (Lapland and neighbouring areas, including Kuusamo).

## Checklists

### Prenanthoidea-group

#### Hieracium karelorum

(Norrl.) Norrl.

7429C407-3241-5EE0-9CC1-CEE97365C129

Synonym: *Hieraciummultiglandulosum* Üksip

##### Native status

Native. Birch forests, shrubs, forest margins.

##### Distribution

Finland: Sa, Kl, Sb, Kb; northern European Russia (Schljakov 1989, Sennikov 2000, Sennikov 2008b) reaching as far as Tver Region to the east (Sennikov 2006b).

##### Diagnosis

Stems 60–100 cm tall. Basal leaves withering at anthesis; cauline leaves 13–18, rather densely spaced, distinctly panduriform, base amplexicaul to broadly subrotund, apex broadly triangular, without teeth, rigid, pale green, glabrous, but with few stellate hairs above, sessile. Phyllaries oblong, with subobtuse apex, 8–9(10) mm long, dark grey-green, with solitary to rather rare simple hairs ca. 0.5 mm long, with dense to abundant glandular hairs (0.4–0.6(0.5–0.8(1) mm long and nearly without stellate hairs, with very narrow glabrous margins, apex without ciliae. Synflorescence branches with solitary to rather rare simple hairs, abundant glandular hairs 0.2–0.3 mm long and stellate hairs. Styles with black papillae. Ligules ciliate.

### Aestiva-group

#### Hieracium angustum

Lindeb.

133F202A-26F8-5E4A-857A-762E78DD4209

##### Native status

Native. Forest margins, riversides.

##### Distribution

Finland: Om, Lks; Norway, Sweden (Samuelsson 1954), northern European Russia (Sennikov 1999a).

##### Diagnosis

Stems 40–60 cm tall. Basal leaves withering at anthesis; cauline leaves 10–15, densely spaced, narrowly oblong to slightly panduriform, base narrowly subrotund to auriculate, apex triangular, with a few small teeth, rigid, dark green, glabrous, but with few stellate hairs above, sessile. Phyllaries oblong, with rather obtuse apex, 9–10 mm long, dark grey-green, with or without solitary simple hairs ca. 1 mm long, with rare to sparse glandular hairs 0.2–0.4 mm long along the central line and rare to sparse dense stellate hairs, with broad glabrous margins, apex with indistinct short ciliae. Synflorescence branches usually without simple and glandular hairs, with stellate hairs. Styles with black papillae. Ligules glabrous.

#### Hieracium condylodes

Brenner

66D67C31-2C6E-5204-99A4-E80677527FE4

Synonym: *Hieraciumpolycomum* Dahlst. ex Norrl.

##### Native status

Native. Riversides, shrubs, tundra, sparse birch forests.

##### Distribution

Finland: Obu, Ks, Lkk, Lks, Le; Norway, Sweden, northern European Russia (Schljakov 1989, Sennikov 1999a, Sennikov 2008b).

##### Diagnosis

Stems 60–80 cm tall. Basal leaves withering at anthesis; cauline leaves 15–20, densely spaced, narrowly oblong to slightly panduriform, base narrowly subrotund to broadly cuneate, apex triangular, with 3–5 small broad teeth in the middle part, rigid, dark green, glabrous, but with few stellate hairs above, sessile. Phyllaries oblong, with obtuse apex, 9–10 mm long, dark grey-green, with very rare to sparse (rather dense) simple hairs ca. 1 mm long, rather dense to dense glandular hairs 0.1–0.4(0.6) mm long and sparse to rather dense stellate hairs, with narrow glabrous margins, apex with indistinct short ciliae. Synflorescence branches with sparse to dense stiff black-based simple hairs 0.5–0.8 mm long, rare to rather dense glandular hairs 0.2–0.3 mm long and stellate hairs. Styles with black papillae. Ligules glabrous.

#### Hieracium crocatum

Fr.

BD615D18-65B2-5034-8A72-AAE453E9E6F7

##### Native status

Native. Riversides, shrubs, tundra, sparse birch forests.

##### Distribution

Finland. Ok, Obu, Ks, Lkk, Lks; Norway, Sweden (Samuelsson 1954), northern European Russia (Sennikov 1999a).

##### Diagnosis

Stems 60–80 cm tall. Basal leaves withering at anthesis; cauline leaves 10–18, densely spaced, oblong to slightly panduriform, base broadly subrotund, apex broadly triangular, with 3–5 small broad teeth in the middle part, rigid, dark green, glabrous and usually without stellate hairs above, sessile. Phyllaries broadly triangular to oblong, with rather obtuse apex, 10–11 mm long, blackish grey-green, with very rare to rather rare simple hairs 1–2 mm long, sparse to rather dense glandular hairs 0.1–0.3(0.5–0.9) mm long and usually without stellate hairs, with narrow glabrous margins, apex with indistinct short ciliae. Synflorescence branches with very rare to sparse (dense) black-based simple hairs 0.5–1 mm long, solitary (to sparse) glandular hairs 0.1–0.2 mm long and stellate hairs. Styles with black papillae. Ligules glabrous.

#### Hieracium pruiniferum

(Norrl.) Norrl.

8B06D26A-AC48-50FE-8FDD-76117B45B3D5

##### Native status

Native. Birch forests, shrubs, forest margins.

##### Distribution

Finland: Ka, Sa, Kl, Sb, Kb; northern European Russia (Schljakov 1989, Sennikov 2008b).

##### Diagnosis

Stems 60–100 cm tall. Basal leaves withering at anthesis; cauline leaves 25–30, densely spaced, indistinctly panduriform to lanceolate-ovate, base broadly subrotund, apex triangular, with a few indistinct minute teeth in the middle, rather rigid, pale green, glabrous, but with few stellate hairs above, sessile. Phyllaries oblong, with broadly acute apex, ca. 10 mm long, grey-green, without simple hairs, with dense glandular hairs 0.2–0.6 mm long and nearly without stellate hairs, with rather broad glabrous margins, apex without ciliae. Synflorescence branches with very rare to sparse glandular hairs 0.1–0.2 mm long and stellate hairs. Styles with black papillae. Ligules glabrous.

#### Hieracium pseudohypochnoodes

Schljakov

928E5E75-10F2-550D-9667-E02CDC4C85CA

Synonym: *Hieraciumsubhypochnoodes* H.Lindb.

##### Native status

Native. Birch forests.

##### Distribution

Finland: Sb; northern European Russia (Schljakov 1989).

##### Diagnosis

Stems 80–100 cm tall. Basal leaves withering at anthesis; cauline leaves ca. 25, densely spaced, narrowly lanceolate-ovate, base narrowly subrotund, apex narrowly triangular, with 4–5 small acute teeth in the basal half, rigid, pale green, glabrous, but with sparse stellate hairs above, sessile. Phyllaries oblong-triangular, with broadly acute apex, 9–10 mm long, dark grey-green, with solitary simple hairs 0.5–0.8 mm long, dense glandular hairs 0.2–0.6 mm long and nearly without stellate hairs, with broad glabrous margins, apex without ciliae. Synflorescence branches with very rare to sparse simple hairs up to 0.5 mm long, very rare glandular hairs 0.1–0.2 mm long and stellate hairs. Styles with black papillae. Ligules glabrous.

### Umbellata-group

#### Hieracium umbellatum

L.

69C33EF2-F21A-5022-BD7E-F85FCEE5DA12

##### Native status

Native. Sparse forests, forest margins, riverside meadows, shrubs.

##### Distribution

Finland: whole territory; Northern (extratropical) Eurasia, North America (POWO 2025).

##### Diagnosis

Stems 60–120 cm tall. Basal leaves rudimentary, withering at anthesis. Cauline leaves 10–30, oblong-lanceolate, oblong-ovate, oblong or linear, base cuneate, apex narrowly triangular, with sparse minute or long narrow teeth, rigid, rather dark-green, glabrous with sparse stellate hairs above, sessile. Phyllaries narrowly oblong, with acute apex, 8–11 mm long, pale to dark grey-green, with or without solitary simple hairs and short glandular hairs, usually with rare to sparse stellate hairs throughout, apex without ciliae. Synflorescence branches usually without simple and glandular hairs, with dense stellate tomentum. Styles with yellow or black papillae. Ligules glabrous.

### Rigida-group

#### Hieracium archaeum

Norrl.

A70564CD-C07C-55EB-9633-44BF83AA11BA

##### Native status

Native. Open forest margins, shrubs.

##### Distribution

Finland: Al. Not known elsewhere, possibly endemic to Finland.

##### Notes

Erroneously included in *Hieraciumsubrigidum* in POWO (2025).

##### Diagnosis

Stems 60–80 cm tall. Basal leaves withering at anthesis; cauline leaves 10–12, sparse in the basal half, dense in the upper half of the stem, rapidly decreasing in size from the middle of the stem, lowermost ones broadly elliptic-ovate to broadly rhombic-ovate, base cuneate, apex broadly triangular to subobtuse, with small sparse teeth in the basal half, rigid, dark green with purple spots, with sparse to rather dense simple hairs 1–1.5 mm long and sparse stellate hairs above, on distinct petioles; middle ones broadly lanceolate to ovate-lanceolate, base broadly cuneate. Phyllaries triangular, with acute apex, ca. 10 mm long, grey-green, with rather rare to sparse simple hairs 1.5–2 mm long, very dense glandular hairs 0.5–1(1.5) mm long and sparse stellate hairs along the margins, apex with an indistinct coma of very short ciliae. Synflorescence branches with rare to sparse simple hairs, rather dense glandular hairs 0.2–0.5 mm long and stellate hairs. Styles with blackish (discoloured) papillae. Ligules glabrous.

#### Hieracium avae

Dahlst. ex Johanss.

F3DA955A-00CC-5F1F-B895-280CBD1669AE

##### Native status

Native. Open forest margins, shrubs.

##### Distribution

Finland: Al, Ab; Sweden (Johansson 1929).

##### Diagnosis

Stems 60–70 cm tall. Basal leaves reduced in size, usually withering at anthesis; cauline leaves 11–13, gradually decreasing in size from the stem base, lowermost ones lanceolate to rhombic-lanceolate, base cuneate, apex triangular, with sparse prominent teeth in the basal half, rigid, dark green, with rather dense simple hairs along the margins and numerous stellate hairs above, on short petioles; middle ones similar. Phyllaries broadly triangular, with acute apex, ca. 9 mm long, blackish, with very rare to rare black-based simple hairs ca. 1 mm long, very dense glandular hairs 0.3–0.5(0.7) mm long and nearly without stellate hairs, apex without ciliae. Synflorescence branches with or without simple to rare simple hairs, without glandular hairs, with dense stellate pubescence. Styles with black papillae. Ligules glabrous.

#### Hieracium cornigerum

Norrl.

7CCDE625-03AF-5C2B-869A-882BBDEF1D50

##### Native status

Native. Open forest margins, sparse forests.

##### Distribution

Finland: St, Ta, Oa, Tb, Sb, Kb, Om. Endemic to Finland.

##### Diagnosis

Stems 50–75 cm tall. Basal leaves 2–3, present at anthesis. Cauline leaves narrowly lanceolate, attenuated into a short, but distinct petiole, apex narrowly obtuse, usually with long rather narrow teeth pointing sidewards, rigid, pale-green, with sparse simple hairs 0.3–0.7 mm and numerous stellate hairs above, lower ones on conspicuous petioles. Phyllaries narrowly triangular, with acute apex, 8–9 mm long, intensely grey-green, with solitary simple hairs at the base, sparse glandular hairs 0.3–0.5 mm long along the middle line and scattered stellate hairs in the basal half, apex without ciliae. Synflorescence branches without simple hairs, sometimes with solitary glandular hairs 0.2 mm long and dense stellate hairs. Styles with black papillae. Ligules glabrous.

#### Hieracium floccimarginatum

Brenner

479B8191-6CD3-52C5-A19D-A8625DC71CE6

##### Native status

Native. Pine forests, forest margins, forested rocks.

##### Distribution

Finland: Ab, N. Endemic to Finland.

##### Diagnosis

Stems 50–70 cm tall. Basal leaves lanceolate to narrowly rhombic-lanceolate, 3–4; cauline leaves 18–25, rapidly decreasing in size from the very base, lowermost ones narrowly rhombic-lanceolate to ovate-lanceolate, base cuneate, apex narrowly attenuated, with prominent narrow teeth, rigid, grass-green, glabrous with abundant stellate hairs above, on indistinct petioles. Phyllaries narrowly triangular, with narrowly acute apex, 9–10 mm long, pale grey-green, without simple hairs, with sparse to rather dense glandular hairs 0.1–0.5 mm long along the central line and sparse stellate hairs in the basal half, apex without ciliae. Synflorescence branches without simple and glandular hairs, with dense stellate tomentum. Styles with black papillae. Ligules glabrous.

#### Hieracium godbyense

(Norrl.) Norrl.

56EC586E-5E59-522F-BCF7-21993762A42F

##### Native status

Native. Open forest margins, shrubs.

##### Distribution

Finland: Al. Not known outside Finland, probably endemic.

##### Notes

This species was treated broadly and reported from the neighbouring territories in the recent sources (Hackman and Sennikov 1998, Sennikov 2000, Sennikov 2003b, POWO 2025). Its actual distribution and distinction from *H.griseellum* should be studied.

##### Diagnosis

Stems 70–100 cm tall. Basal leaves 2–3, partly withering at anthesis; cauline leaves 11–13, evenly distributed along the stem, lowermost ones lanceolate, base cuneate, apex narrowly triangular, with sparse small teeth, rigid, grass-green, with rather dense simple hairs 1.5–2 mm long and sparse stellate hairs above, on indistinct petioles. Phyllaries triangular, with acute apex, 9–10 mm long, grey-green, with rather rare black-based simple hairs ca. 1 mm long, dense glandular hairs 0.2–0.4 mm long and rather dense stellate hairs throughout (somehow resembling the phyllaries of *H.vulgatum*), apex with few short ciliae. Synflorescence branches with sparse to rather dense simple hairs, rather dense glandular hairs 0.1–0.2 mm long and stellate hairs. Styles with black papillae. Ligules glabrous.

#### Hieracium griseellum

Brenner

AC252A15-9677-53A1-9A19-80CB5E9E6ADF

Synonym: *Hieraciummixopolium* Dahlst.

##### Native status

Native. Open forest margins, shrubs.

##### Distribution

Finland: Al, Ab, N, Ta, Sa; Sweden (Johansson 1929), northern European Russia (Schljakov 1989, Sennikov 2000), Estonia (Sennikov 2003b).

##### Notes

This species was included in *Hieraciumgodbyense* in the recent sources (Hackman and Sennikov 1998, Sennikov 2000, Sennikov 2003b, POWO 2025).

##### Diagnosis

Stems 70–110 cm tall. Basal leaves 1–3, withering or present at anthesis; cauline leaves 15–20, evenly and densely situated on the stem, gradually decreasing in size from the stem base, lowermost ones lanceolate to oblong-lanceolate, base cuneate, apex broadly triangular, with sparse small to prominent acute teeth, rigid, dark green, with sparse simple hairs 0.5–1 mm long (largely along the margins) and numerous stellate hairs above, on indistinct petioles, middle ones ovate-lanceolate, base broadly cuneate. Phyllaries narrowly triangular, with acute apex, ca. 9 mm long, grey-green, with rather rare to sparse black-based simple hairs 0.5–1 mm long, rather dense glandular hairs 0.3–0.5 mm long along the middle line and dense stellate hairs throughout (much resembling the phyllaries of *H.vulgatum*), apex without ciliae. Synflorescence branches with very rare simple hairs, solitary glandular hairs 0.1–0.2 mm long and stellate hairs. Styles with blackish (discoloured) papillae. Ligules glabrous.

#### Hieracium impunctatum

Norrl.

27844F78-7C8D-5DB5-9331-B7EE8E0D8668

##### Native status

Native. Forest margins, shrubs.

##### Distribution

Finland: N, St, Oa. Endemic to Finland.

##### Diagnosis

Stems 60–80 cm tall. Rosulate leaves usually withering by anthesis, 1–2, lanceolate, with narrowly serrate teeth, rigid, dark-green, nearly glabrous above, on rather short petioles. Cauline leaves lanceolate, rhombic-lanceolate or ovate-lanceolate, sessile to shortly petiolate, 7–10. Phyllaries with a rather obtuse apex, 10–11 mm long, grey-green, with sparse black simple hairs up to 1 mm long in the basal part, dense glandular hairs 0.8–1.3 mm long and rather dense stellate hairs throughout, but more along the margins, apex with a short coma and abundant short ciliae. Synflorescence branches with rare simple hairs, sparse glandular hairs ca. 0.2 mm long and stellate hairs. Styles with pale dark papillae. Ligules glabrous.

#### Hieracium internatum

Brenner

C84AB179-3CD7-5718-88B5-F76A1C5F04E9

##### Native status

Native. Forest margins, shrubs.

##### Distribution

Finland: Al, N, Oa. Endemic to Finland.

##### Diagnosis

Stems 60–90 cm tall. Rosulate leaves usually withering by anthesis, 1–2, lanceolate, with narrowly serrate teeth, rigid, dark-green, nearly glabrous above, on rather short petioles. Cauline leaves lanceolate, rhombic-lanceolate or ovate-lanceolate, sessile to shortly petiolate, 8–12. Phyllaries with an obtuse apex, 10–11 mm long, blackish-green, with solitary to sparse black simple hairs up to 1 mm long in the basal part, dense glandular hairs 0.7–1.2 mm long and scattered stellate hairs mostly along the margin, apex with a poorly visible coma and abundant short ciliae. Synflorescence branches with rare simple hairs, sparse glandular hairs 0.2–0.3 mm long and stellate hairs. Styles with black papillae. Ligules glabrous.

#### Hieracium lapponicum

Fr.

621B5E02-3AB3-5715-9659-EDA2B9890BD7

##### Native status

Native. Riversides, tundra.

##### Distribution

Finland: Obu, Ks, Lkk, Lks, Le, Li; Norway, Sweden, northern European Russia (Schljakov 1989).

##### Diagnosis

Stems 30–40 cm tall. Basal leaves usually present at anthesis, lanceolate or rhombic-lanceolate; cauline leaves 5–7, gradually decreasing from the stem base, lowermost ones lanceolate, base narrowly cuneate, apex acute, with sparse minute teeth, rather dark green, rigid, with sparse to rather dense simple hairs 0.8–1 mm long and sparse stellate hairs above, on sessile or on indistinct petioles. Phyllaries broadly triangular, with broadly acute to obtuse apex, 9–10 mm long, blackish, with rare to rather rare black-based simple hairs 0.5–1 mm long, rare glandular hairs 0.1–0.3 mm long and sparse stellate hairs throughout, apex with few very short ciliae. Synflorescence branches with rare to sparse (rather dense) simple hairs, usually without glandular hairs and with stellate pubescence. Styles with black papillae. Ligules glabrous.

#### Hieracium laterale

(Norrl.) Brenner

3BCCA185-CB0F-5513-A897-97D38C0252DD

##### Native status

Native. Riversides, tundra.

##### Distribution

Finland: Obu, Lkk, Le, Li; Norway, Sweden, northern European Russia (Schljakov 1989).

##### Diagnosis

Stems 35–45 cm tall. Basal leaves usually present at anthesis, lanceolate; cauline leaves 6–8, gradually decreasing from the stem base, lowermost ones lanceolate to narrowly rhombic-lanceolate, base attenuated into a petiole, apex narrowly triangular, with sparse small teeth, rigid, rather dark green, with sparse simple hairs 1–1.5 mm long and abundant stellate hairs above, on short, but distinct petioles. Phyllaries broadly triangular, with a broad apex, 10–11 mm long, dark grey-green, with solitary to rare (or lacking) black-based simple hairs 1–1.3 mm long, rather dense glandular hairs 0.3–0.7(1) mm long and very rare (nearly lacking) stellate hairs, apex with very short ciliae. Synflorescence branches with solitary to rather rare (sparse) simple hairs, solitary to rather rare (dense) glandular hairs 0.2–0.3 (0.5) mm long and stellate hairs. Styles with black papillae. Ligules glabrous.

#### Hieracium lissolepium

Johanss. & Sam.

3C3A1815-E9D2-5A7D-B931-283ABB600789

##### Native status

Native. Open forest margins, shrubs.

##### Distribution

Finland: Al, Ab, N, Om; Denmark (Schou 2001), Norway, Sweden (Johansson 1929), eastern Baltic coastline, including Russia and Estonia (Sennikov 2000, Sennikov 2003b). Reported elsewhere in Europe (POWO 2025).

##### Diagnosis

Stems 70–90 cm tall. Basal leaves rudimentary, withering at anthesis. Cauline leaves 13–18, lowermost ones oblong-lanceolate, base attenuated to cuneate, apex narrowly triangular, with sparse narrowly triangular teeth, rigid, dark-green, glabrous with sparse stellate hairs above, on indistinct petioles; middle ones ovate-lanceolate, base broadly cuneate to subrotund, apex attenuated. Phyllaries triangular-oblong, with rather acute apex, 8–9 mm long, pale grey-green, with or without solitary simple hairs 0.5–0.8 mm long, usually without glandular hairs, with sparse to rather dense stellate hairs throughout, apex without ciliae. Synflorescence branches without simple and glandular hairs, with dense stellate tomentum. Styles with pale black (discoloured) papillae. Ligules glabrous.

#### Hieracium obatrescens

(Dahlst.) Dahlst.

429310A3-9C64-59A2-8FED-A5E8808F57B8

##### Native status

Native. Open forest margins, shrubs.

##### Distribution

Finland: Al, Ab; Denmark (Schou 2001), Sweden (Johansson 1929).

##### Diagnosis

Stems 60–90 cm tall. Basal leaves reduced in size, withering at anthesis; cauline leaves 10–13, gradually decreasing in size from the stem base, lowermost ones rhombic-lanceolate to ovate-lanceolate, base narrowly cuneate, apex triangular, with sparse small triangular teeth, grass-green, rigid, with sparse simple hairs 0.5–1 mm long and numerous stellate hairs above, on very short petioles, middle ones ovate-lanceolate, base broadly cuneate, apex long triangular. Phyllaries triangular, with acute apex, 9–10 mm long, grey-green, with rather rare half-black simple hairs 1–1.5 mm long, rather dense glandular hairs 0.5–0.8 mm long and some stellate hairs in the basal part only along the margins, apex without ciliae. Synflorescence branches with rare simple hairs, with solitary glandular hairs 0.2–0.3 mm long and stellate hairs. Styles with black papillae. Ligules glabrous.

#### Hieracium orbolense

(Stenstr.) Dahlst.

B69B34FE-AA5A-54A1-95BC-0560DF778E99

##### Native status

Native. Open forest margins, shrubs.

##### Distribution

Finland: Al, Ab; Norway, Sweden (Samuelsson 1954, Tyler 2017).

##### Diagnosis

Stems 40–50 cm tall. Basal leaves 3–5, usually well-developed at anthesis; cauline leaves 8–10, evenly distributed along the stem, lowermost ones rhombic-lanceolate, base cuneate, apex narrowly triangular, with prominent narrow teeth, rigid, greyish-green, glabrous, but with stellate hairs above, on very short petioles. Phyllaries narrowly triangular, with acute apex, 10–11 mm long, grey-green, with sparse to rather dense simple hairs ca. 1 mm long, sparse to rather dense glandular hairs 0.3–0.5(0.7) mm long and sparse stellate hairs along the margins and in the basal part, apex with numerous short ciliae. Synflorescence branches with rather rare simple hairs, rare glandular hairs 0.2–0.3 mm long and stellate hairs. Styles with black or blackish papillae. Ligules glabrous.

#### Hieracium rasile

Norrl.

AA383BE8-BE63-5942-8585-F9B472FB4461

##### Native status

Native. Open forest margins, shrubs.

##### Distribution

Finland: Oa, Tb, Sb, Om, Ok, Obo, Obu, Ks, Lks. Endemic to Finland.

##### Diagnosis

Stems 40–50 cm tall. Basal leaves 2–3, present at anthesis; cauline leaves 6–8, very gradually decreasing in size from the stem base, lowermost ones rhombic-lanceolate, sessile, base cuneate, apex attenuate-triangular, with sparse (3–4) prominent acute teeth in the basal half, rigid, intensely green, glabrous, but with numerous stellate hairs above. Phyllaries nearly linear-oblong, with subacute apex, ca. 10 mm long, grey-green, with solitary pale simple hairs 0.5–1 mm long, sparse glandular hairs 0.3–0.8 mm long along the narrow central line and sparse stellate hairs mostly in the basal half, apex without ciliae. Synflorescence branches without simple and glandular hairs, with dense stellate tomentum. Styles with blackish (discoloured) papillae. Ligules glabrous.

#### Hieracium savo-karelicum

Norrl.

B68B66CB-E6A8-55FC-A1B9-69619CE5E0F6

##### Native status

Native. Pine forests.

##### Distribution

Finland: Tb, Sb, Kb; northern European Russia (Schljakov 1989, Sennikov 2008b).

##### Diagnosis

Stems 60–70 cm tall. Basal leaves 2–3, present at anthesis. Cauline leaves lanceolate or rhombic-lanceolate, attenuated into a short, but distinct petiole, apex sharply triangular, with prominent very narrow teeth, rigid, grass-green, glabrous, but with scattered stellate hairs above. Phyllaries triangular, with broadly acute apex, 8–9 mm long, grey-green, with rare to rather rare half-black simple hairs 0.7–1 mm long, rather dense black glandular hairs 0.3–0.7 mm long and very rare stellate hairs, apex without ciliae. Synflorescence branches with solitary simple hairs, rare glandular hairs 0.1–0.3 mm long and stellate hairs. Styles with black papillae. Ligules glabrous.

#### Hieracium subaureum

H.Lindb. ex Norrl.

931394BA-81C0-54F0-868D-5455B7237166

##### Native status

Native. Forest margins, sparse forests, shrubs.

##### Distribution

Finland: Sb. Endemic to Finland.

##### Diagnosis

Stems 70–90 cm tall. Basal leaves 1–2, present at anthesis; cauline leaves 10–15, gradually decreasing in size from the stem middle, lowermost ones lanceolate or rhombic-lanceolate, base cuneate, apex triangular, with sparse narrow teeth, rigid, greyish-green, with sparse simple hairs 0.5–0.8 mm long and stellate hairs above, attenuated into very short petioles, middle ones narrowly rhombic lanceolate, broadly attenuate-petiolate. Phyllaries oblong, with subobtuse apex, ca. 9 mm long, grey-green, with or without solitary simple hairs 0.5–0.8 mm long in the basal part, with rather dense to dense glandular hairs 0.1–0.5 mm long and nearly without stellate hairs, apex without ciliae. Synflorescence branches without simple hairs, with very rare glandular hairs 0.1–0.2 mm long and stellate hairs. Styles with black papillae. Ligules glabrous.

#### Hieracium subrigidum

(Almq. ex Stenstr.) Dahlst.

F2B8A1B6-7A0F-593E-8FE3-8AEC67EF6EBC

##### Native status

Native. Shrubs, forest margins.

##### Distribution

Finland: Al, N, St, Sa, Oa; Sweden, possibly Norway. Reported from northern European Russia (Schljakov 1989).

##### Diagnosis

Stems 50–70 cm tall. Rosulate leaves narrowly lanceolate, base gradually attenuated into a petiole, apex triangular, with sparse small narrow teeth, rigid, grass-green, with sparse simple hairs 1–1.2 mm long and stellate hairs above, on very short petioles. Cauline leaves rhombic-lanceolate or oblong-lanceolate, sessile, base broadly rotund, with prominent very narrow spreading teeth. Phyllaries broadly linear, with acute apex, 10–11 mm long, blackish-green, with rather dense dark-based simple hairs 1–1.3(1.5) mm long, rare to sparse glandular hairs 0.2–0.5 mm long and almost without stellate hairs, apex without ciliae. Synflorescence branches with rather rare simple hairs, with solitary or without glandular hairs 0.2 mm long, with stellate pubescence. Styles with black papillae. Ligules glabrous.

#### Hieracium tersiflorum

Norrl.

F4B5249B-F4A3-500F-9080-3B181C9860CA

##### Native status

Native. Shrubs.

##### Distribution

Finland: Obu; possibly also in Norway (POWO 2025).

##### Diagnosis

Stems 30–40 cm tall. Rosulate leaves very few, lanceolate to rhombic-lanceolate, base attenuate to the petiole, apex narrowly triangular, with sparse serrate teeth, rigid, dark-green, with sparse to rather dense simple hairs 1.5–2.5 mm long and sparse or rather dense stellate hairs above, on short petioles. Cauline leaves similar, 4–7. Phyllaries broadly triangular, with rather obtuse apex, 10–11 mm long, grey-green, with rather dense white simple hairs 0.8–1(1.3) mm long, rather dense glandular hairs 0.1–0.3 mm long and rare to sparse stellate hairs throughout, apex with a small coma of short ciliae. Synflorescence branches without or with solitary simple hairs, solitary glandular hairs 0.2 mm long and stellate hairs. Styles with dark papillae. Ligules glabrous.

### Tridentata-group

#### Hieracium crepidioides

Brenner

53F2D9AC-430F-5DA3-B2E9-98E8F06D2C1B

##### Native status

Native. Riversides, shrubs.

##### Distribution

Finland: Obu, Lkk, Lks, Le; Sweden (Johansson 1929), northern European Russia (Schljakov 1989).

##### Diagnosis

Stems 40–60 cm tall. Basal leaves usually withering at anthesis; cauline leaves 8–10, gradually decreasing, lowermost ones lanceolate to oblong-lanceolate, base cuneate to broadly attenuate, apex broadly triangular, with sparse large teeth, rather rigid, dark green, glabrous, but with rather dense stellate hairs above, sessile. Phyllaries (broadly) triangular, with acute apex, ca. 9 mm long, dark grey-green, with a few simple hairs 1 mm long at the base only, rather rare to sparse glandular hairs 0.3–0.5 mm long along the narrow middle line and rather dense stellate hairs throughout, apex with short ciliae. Synflorescence branches with sparse simple hairs and rare glandular hairs 0.2–0.3 mm long mostly under the central head, with stellate pubescence. Styles with black papillae. Ligules glabrous.

#### Hieracium cruentiferum

(Norrl. & H.Lindb.) Brenner

E07EC28C-DA40-5639-8C88-189D8B5875CA

##### Native status

Native. Sparse forests, forest margins.

##### Distribution

Finland: Sa, Oa, Tb, Sb, Om, Ok, Obo, Obu, Lks; Sweden (Johansson 1929), northern European Russia (Schljakov 1989).

##### Diagnosis

Stems 70–100 cm tall. Basal leaves withering at anthesis; cauline leaves 8–12, gradually decreasing from the middle of the stem, lowermost ones long-lanceolate, base narrowly cuneate to attenuated, apex narrowly triangular, with sparse long narrow teeth, rigid, dark green with abundant violet spots, glabrous, but with rather dense stellate hairs above, on short petioles. Phyllaries triangular, with acute apex, ca. 10 mm long, dark grey-green, with a few simple hairs 0.5 mm long at the base only, rather rare to sparse glandular hairs 0.3–0.8 mm long along the narrow middle line and sparse stellate hairs throughout, apex with short ciliae. Synflorescence branches with or without very rare simple hairs and glandular hairs 0.1–0.2 mm long, with stellate pubescence. Styles with black papillae. Ligules glabrous.

#### Hieracium dolabratum

(Norrl.) Norrl.

8BEE9E8C-776E-58E7-A860-AF8E1440BCC4

##### Native status

Native. Riversides, shrubs, tundra.

##### Distribution

Finland: Ok, Obu, Ks, Lkk, Lks, Le, Li; Norway, Sweden (Johansson 1929), northern European Russia (Schljakov 1989, Sennikov 2008b).

##### Diagnosis

Stems 30–70 cm tall. Basal leaves reduced in size, often withering at anthesis; cauline leaves 5–10, decreasing in size from the stem base, lowermost ones narrowly oblong-lanceolate, base narrowly cuneate to attenuated, apex narrowly triangular, with sparse minute teeth, rigid, greyish-green, glabrous, but with sparse to rather dense stellate hairs above, on indistinct petioles. Phyllaries (narrowly) triangular, with acute apex, 9–10 mm long, dark grey-green, without or with solitary to very rare black-based to half-black simple hairs 0.5–1 mm long, sparse glandular hairs 0.2–0.5 mm long mostly along the middle line and sparse to rather dense stellate hairs throughout, apex without ciliae. Synflorescence branches usually without simple and glandular hairs and with stellate pubescence. Styles with black papillae. Ligules glabrous.

#### Hieracium linifolium

Saelan ex Lindeb.

0C563ADA-F141-58A9-B534-79114D98ABF5

##### Native status

Native. Open rocks.

##### Distribution

Ab, N, Oa. Likely endemic to Finland, although reported elsewhere due to the expanded species concept (Johansson 1929, Schljakov 1989, Sennikov 2003b).

##### Diagnosis

Stems 40–60 cm tall. Basal leaves 1–2, much reduced, present at anthesis; cauline leaves 10–15, evenly distributed along the stem, very long below and gradually decreasing in size from the basal part of the stem (plants of pyramidal shape), linear to very narrowly ovate-lanceolate, base narrowly cuneate, apex long attenuated, with sparse small teeth, rigid, dark-green, glabrous and with sparse stellate hairs above, sessile. Phyllaries triangular, with acute apex, 9–10 mm long, grey-green, with rather rare dark-based pale rigid simple hairs 1–1.5 mm long, rather dense glandular hairs 0.2–0.5 mm long along the central line and rather rare stellate hairs mostly in the basal part, apex with indistinct ciliae. Synflorescence branches without simple and glandular hairs, with dense stellate pubescence. Styles with discoloured (yellowish) papillae. Ligules glabrous.

#### Hieracium subumbellatum

(Norrl.) Brenner

569A3496-AB7D-5D9B-B0D1-C5081BAF8067

Synonym: *Hieraciumsemiumbellatum* Norrl.

##### Native status

Native. Sparse forests, forest margins.

##### Distribution

Finland: Sa, Oa, Sb, Om, Ok, Obu. Possibly endemic to Finland.

##### Diagnosis

Stems 50–80 cm tall. Rosulate leaves absent; cauline leaves 10–15, evenly distributed along the stem and gradually decreasing in size in the upper part, linear-lanceolate, base cuneate, apex long and broadly attenuate, with sparse coarse teeth, rigid, pale greyish-green, glabrous and with stellate hairs above, sessile or on minute petioles. Phyllaries oblong-triangular, with acute apex, ca. 9 mm long, grey-green, without simple hairs, with rather dense glandular hairs 0.3–0.8(1–1.5) mm long mostly along the central line and rare to sparse stellate hairs mostly in the basal part, apex with indistinct ciliae. Synflorescence branches without simple and glandular hairs, with dense stellate pubescence. Styles with black papillae. Ligules glabrous.

#### Hieracium succedaneum

Brenner

051C4AA0-FB1A-5EEE-8119-170FF59E475B

##### Native status

Native. Shrubs, riverside meadows.

##### Distribution

Finland: Ok, Obo. Endemic to Finland.

##### Diagnosis

Stems 60–70 cm tall. Basal leaves 2–3, partly withering at anthesis; cauline leaves 10–12, evenly distributed along the stem, gradually decreasing in size from the stem base, lowermost ones broadly linear-lanceolate or oblong-lanceolate, base cuneate, apex narrowly triangular, with sparse minute to small teeth, rather rigid, glabrous, but with numerous stellate hairs above, sessile or attenuated into a very brief petiole, middle ones narrowly ovate-lanceolate, base broadly cuneate, apex attenuated. Phyllaries narrowly triangular, with acute apex, ca. 9 mm long, grey-green, with rather rare black-based simple hairs 0.5–1 mm long in the basal half, sparse glandular hairs 0.3–0.8 mm long along the central line and rare stellate hairs throughout, apex with short ciliae. Synflorescence branches with solitary or very rare simple hairs, solitary or very rare glandular hairs 0.1–0.3 mm long and stellate hairs. Styles with black papillae. Ligules glabrous.

### Oreadea-group

#### Hieracium argenteum

(Fr.) Fr.

D463820B-A34C-500D-BB50-CFFA13329A3B

Synonym: *Hieraciumbottnicum* Dahlst. ex Norrl.

##### Native status

Native. Open and forested rocks.

##### Distribution

Finland: Ab, N, St, Oa; Norway, Sweden (Samuelsson 1954, Tyler 2011).

##### Notes

Tyler (2011) doubted the application of this species name (which remains formally unresolved) and suggested using the name *Hieraciumanodon* Brenner instead.

##### Diagnosis

Stems 15–25 cm tall, with simple hairs 3–3.5 mm long at the base. Rosulate leaves lanceolate to broadly lanceolate, with minute to prominent narrow teeth, thick, intensely plumbeous, glabrous above, margin with simple hairs 1.5–3 mm long, on very short petioles. Cauline leaves similar, 1–3 at the stem base. Phyllaries narrowly triangular, attenuated to the apex, 10–12 mm long, blackish, with sparse simple hairs 1.5–2.5 mm long, dark glandular hairs 0.3–0.5 mm long and stellate hairs throughout, apex with numerous long ciliae. Synflorescence branches with solitary simple hairs, rather dense glandular hairs 0.1–0.4 mm long and stellate hairs. Styles with dark papillae. Ligules glabrous.

#### Hieracium crinellum

Omang

CEDA9657-7E68-5C0D-9807-D816CC273D66

Synonym: Hieraciumschmidtiisubsp.residuum Norrl., **syn. nov.** Its lectotype (**designated here**): Finland. Tavastia australis: Kuhmois å en högt berg vid Isojärvi, 19.07.1866, *J.P. Norrlin* (H, isolectotype H).

##### Native status

Native. Sparsely forested rocks.

##### Distribution

Finland: Ta; Norway, Sweden (Tyler 2011). The Finnish occurrence of the species is rather unusual. It is remotely isolated from the Scandinavian part of its distribution area and also distinctly separated from the main distribution of the Oreadea-group in Finland, which is confined to the coastal areas of the Gulf of Bothnia and the Gulf of Finland. Nevertheless, *Hieraciumsubonosmoides* shows a similar extension to the mainland rocks of Tavastia australis and apparently represents a similar relic occurrence there.

##### Notes

Hieraciumschmidtiisubsp.residuum Norrl. has been long known as a local endemic of Tavastia australis in southern Finland, readily differring from the other Finnish taxa of the Oreadea-group in a longer pubescence on its stems and leaves (Norrlin 1906, Hackman and Sennikov 1998). The recent treatment of the Oreadea-group in Sweden (Tyler 2011) revealed that the only taxon with the longer hairs in this country is *H.crinellum*, which is also dissimilar from the other Swedish taxa. Our examination of the lectotype specimen of H.schmidtiisubsp.residuum (Fig. 2), which remains the only collection of the taxon in Finland since its collection year (1866), unambiguously shows its identity to the Swedish collections, described by Tyler, not only in the long simple hairs on the whole plant, but also in the characteristically narrow phyllaries with numerous long ciliae on the top. A new synonymy is consequently proposed here.

##### Diagnosis

Stems 25–35 cm tall. Rosulate leaves narrowly oblong, base broadly cuneate, apex broadly triangular, with repand margins, thick and rigid, plumbeous-green, glabrous above, with simple hairs 4–5 mm long along the margins, on rather long petioles. Cauline leaf single, usually strongly reduced. Phyllaries very narrowly triangular, with very narrowly attenuated apex, 11–12 mm long, grey-green, with sparse to rather dense simple hairs 2–3 mm long, rather dense glandular hairs 0.1–0.3 mm long and sparse to rather dense stellate hairs throughout, apex with long ciliae. Synflorescence branches with sparse simple hairs, abundant glandular hairs 0.1–0.2 mm long and stellate hairs. Styles with yellow papillae. Ligules glabrous.

#### Hieracium cuspidifolium

Brenner

97E922C8-33E4-5613-AA3E-5F814C89EFD8

##### Native status

Native. Sparse forests, forest margins.

##### Distribution

Finland: Ab, N, Om. Endemic to Finland.

##### Diagnosis

Stems 50–75 cm tall, with simple hairs 1.5–2 mm long at the base. Basal leaves withering at anthesis; cauline leaves 10–15, gradually decreasing in size from the stem base, lowermost ones very narrowly ovate-lanceolate (nearly linear), base narrowly cuneate to attenuated, apex narrowly attenuated, with sparse long narrow teeth, rigid, dark-green, glabrous, but with rather dense stellate hairs above, with rare simple hairs 1–1.5 mm long along the margins, on indistinct petioles. Phyllaries triangular, with narrowly acute apex, ca. 11 mm long, dark grey-green, with rare to sparse black-based simple hairs 0.5–0.8 mm long, dense glandular hairs 0.1–0.3 mm long on the whole surface and rather dense stellate hairs in the basal part, apex without ciliae. Synflorescence branches with or without very rare simple hairs and glandular hairs 0.1–0.2 mm long, with stellate pubescence. Styles with yellow papillae. Ligules glabrous.

#### Hieracium norvegicum

Fr.

231449C3-463E-5FDA-BFA4-FDE98EC4A0F1

##### Native status

Native. Open forest margins, shrubs.

##### Distribution

Finland: Ab, N, St; Norway, Sweden (Tyler 2011).

##### Diagnosis

Stems 60–80 cm tall. Basal leaves withering at anthesis; cauline leaves 11–13, gradually decreasing from the stem base, lowermost ones narrowly lanceolate, base narrowly cuneate, apex narrowly triangular, attenuate, with narrowly acute teeth, rigid, dark green, glabrous with sparse stellate hairs above, with simple hairs ca. 1.5 mm long along the margins, on indistinct petioles, middle ones similar. Phyllaries triangular, with acute apex, ca. 10 mm long, dark grey-green, with sparse to rather dense whitish simple hairs 0.5–1 mm long, dense glandular hairs 0.1–0.3 mm long and sparse stellate hairs along the margins, apex without ciliae. Synflorescence branches without simple hairs, with sparse glandular hairs 0.1 mm long and stellate hairs. Styles with black papillae. Ligules glabrous.

#### Hieracium rufescens

(Fr.) Dahlst.

74BD2F3A-A4B1-5AE4-BE7F-961B9A0A08B1

##### Native status

Native. Open and forested rocks.

##### Distribution

Finland: Al, Ab, N, St; Sweden (Tyler 2011).

##### Diagnosis

Stems 40–70 cm tall. Rosulate leaves largely withering at anthesis; cauline leaves 7–15, lanceolate to ovate-lanceolate, base cuneate, apex narrowly triangular to attenuate, with sparse small, but prominent teeth, rigid, dark glaucous-green, glabrous above, with simple hairs 0.5–1 mm long along the margins, on short petioles. Phyllaries narrowly triangular, with narrowly acute apex, 9–10 mm long, grey-green, with rather rare to sparse simple hairs 0.7–1 mm long, rather dense glandular hairs 0.2–0.3(0.5) mm long and rather dense stellate hairs throughout, apex with a small coma of short ciliae. Synflorescence branches usually without simple hairs, with solitary to very rare glandular hairs 0.1–0.2 mm long and stellate hairs. Styles with yellow papillae. Ligules glabrous.

#### Hieracium saxifragum

Fr.

42C72422-C305-55F2-9859-C79672A50275

##### Native status

Native. Open and forested rocks.

##### Distribution

Finland: Al, Ab, N, St; Norway, Sweden (Samuelsson 1954), Estonia (Sennikov 2003b), north-western European Russia (Sennikov 2000).

##### Notes

Tyler (2011) circumscribed this species in a restricted sense, possibly excluding the Finnish populations, which he referred to as *Hieraciumlindebergii* (Nym.) Dahlst.

##### Diagnosis

Stems 40–50 cm tall. Rosulate leaves narrowly lanceolate, base narrowly cuneate, apex narrowly triangular, with numerous minute teeth, rather thick and rigid, pale glaucous-green, glabrous above, with simple hairs ca. 2 mm long along the margins, on rather short petioles. Cauline leaves similar, 2–3. Phyllaries very narrowly triangular, with narrowly attenuated apex, ca. 11 mm long, grey-green, with sparse simple hairs 1–1.5 mm long, sparse to rather dense glandular hairs 0.1–0.3(0.4) mm long and sparse to rather dense stellate hairs throughout, apex with numerous long flexuous ciliae. Synflorescence branches with sparse simple hairs, rare to sparse (rather dense) glandular hairs 0.1–0.2(0.3) mm long and stellate hairs. Styles with discoloured papillae. Ligules glabrous.

#### Hieracium subobatrescens

Brenner

D16FACE4-FBB9-5CD5-8316-F36F5D28C94A

##### Native status

Native. Open and forested rocks.

##### Distribution

Finland: Ab, N. Endemic to Finland.

##### Diagnosis

Stems 50–60 cm tall, with simple hairs 2.5–3.5 mm long at the base. Rosulate leaves withering at anthesis; cauline leaves 15–20, linear to narrowly lanceolate, base cuneate, apex narrowly triangular, with sparse small teeth, rather thick and rigid, glaucous-green, glabrous above, with simple hairs 1.5–2 mm long along the margins, on indistinct petioles. Phyllaries broadly triangular, with acute apex, 9–9.5 mm long, grey-green, with sparse pale simple hairs 1–2 mm long, rather dense pale glandular hairs 0.1–0.2(0.3) mm long and sparse stellate hairs mostly along the margins, apex with a small coma of very short ciliae. Synflorescence branches without simple and glandular hairs, with dense stellate hairs. Styles with yellowish papillae. Ligules glabrous.

#### Hieracium subonosmoides

Brenner

F63CF1B2-EB16-5C82-B934-A1E66D8A7B8C

##### Native status

Native. Open and forested rocks.

##### Distribution

Finland: Ab, N, St, Ta. Possibly endemic to Finland.

##### Notes

This species was tentatively included in *Hieraciumextensiforme* Dahlst. by Tyler (2011).

##### Diagnosis

Stems 40–50 cm tall, with simple hairs 2–2.5 mm long at the base. Rosulate leaves lanceolate, base narrowly cuneate, apex triangular, with minute teeth, thick, intensely plumbeous, glabrous above, with simple hairs ca. 1 mm long along the margins, on short petioles. Cauline leaves similar, 4–5. Phyllaries narrowly triangular, with acute apex, 11 mm long, grey-green, with rather dense pale simple hairs ca. 1 mm long, very dense pale glandular hairs 0.1–0.4 mm long and sparse stellate hairs mostly along the margins, apex with rather numerous ciliae. Synflorescence branches with rather dense simple hairs, very dense glandular hairs 0.2–0.3 mm long and stellate hairs. Styles with yellow papillae. Ligules glabrous.

### Subsimilia-group

#### Hieracium caespiticola

Norrl.

E27E73DB-02C6-5775-B411-33F72F81D2FF

##### Native status

Native. Sparse pine forests.

##### Distribution

Finland: Sa, Oa, Tb, Sb, Kb, Om, Ok, Obo, Obu, Ks; Sweden (Tyler 2017), northern European Russia (Samuelsson 1954, Schljakov 1989, Sennikov 2000, Sennikov 2008b).

##### Diagnosis

Stems 40–60 cm tall. Rosulate leaves narrowly lanceolate, base narrowly cuneate to attenuated, apex narrowly triangular, with very small sparse serrate teeth, rigid, grass-green, with sparse simple hairs 0.3–0.5 mm long and stellate hairs above, on short petioles. Cauline leaves similar, 2–3. Phyllaries narrowly triangular, with acute apex, 9–10 mm long, grey-green, without simple hairs, with dense glandular hairs 0.5–0.8 mm long and sparse stellate hairs, apex with numerous ciliae. Synflorescence branches without simple hairs, with dense glandular hairs 0.3–0.5 mm long and stellate hairs. Styles with black papillae. Ligules glabrous.

#### Hieracium delineatum

Norrl.

99EB124D-DB7E-5182-AD96-F210D14BA3E3

##### Native status

Native. Forest margins.

##### Distribution

Finland: St, Ta, Tb. Endemic to Finland.

##### Diagnosis

Stems 25–40 cm tall. Rosulate leaves lanceolate to oblong-lanceolate, with small broad teeth, thin, dark-green with purple spots, with sparse simple hairs 0.8–1.5 mm above, on short petioles. Cauline leaves similar, but reduced in size, 2–3. Phyllaries triangular, with a narrowly triangular apex, 8–9 mm long, grey-green, without simple hairs, with rather dense glandular hairs 0.5–0.8 mm long and sparse stellate hairs in the basal half, apex with indistinct ciliae. Synflorescence branches without simple hairs, with sparse glandular hairs 0.2–0.3 mm long and stellate hairs. Styles with dark papillae. Ligules glabrous.

#### Hieracium improvisum

Norrl.

E2BEE5C1-6370-52AA-BF05-8B4EAECA8312

##### Native status

Native. Sparse forests.

##### Distribution

Finland: Ab, N, St, Ta, Oa, Tb, Sb. Endemic to Finland.

##### Diagnosis

Stems 50–60 cm tall. Rosulate leaves narrowly lanceolate, with small to prominent, narrow teeth, rather thick, pale-green, with rare to sparse simple hairs 0.5–1 mm long above, on short petioles. Cauline leaves similar, 3–5. Phyllaries triangular, with a rather obtuse triangular apex, 8–9 mm long, dark-green, with solitary simple hairs 0.5 mm long at the base only, abundant glandular hairs 0.5–0.8 mm long throughout (including margins) and scattered stellate hairs mostly at the base, apex with very few ciliae. Synflorescence branches without simple hairs, with numerous glandular hairs 0.2–0.4 mm long and stellate hairs. Styles with black papillae. Ligules glabrous.

#### Hieracium resupinatum

(Almq. ex Stenstr.) Dahlst.

F7DFA501-62BD-5214-A7EF-6A67F8FA40A2

##### Native status

Native. Forest margins.

##### Distribution

Finland: Al, Ab, St; Norway, Sweden (Samuelsson 1954, Tyler 2017).

##### Diagnosis

Stems 30–50 cm tall. Rosulate leaves lanceolate, base narrowly attenuated, apex narrowly triangular, with prominent serrate teeth, rigid, greyish-green, with sparse simple hairs ca. 0.5 mm long along the margins above, on short petioles. Cauline leaves similar, petiolate, ca. 5. Phyllaries triangular, with acute apex, 8–9 mm long, grey-green, without simple hairs, with rather dense glandular hairs 0.1–0.4 mm long and sparse stellate hairs throughout and along the margin, apex with numerous short ciliae. Synflorescence branches without simple hairs, with dense glandular hairs 0.1–0.2 mm long and stellate hairs. Styles with black papillae. Ligules glabrous.

#### Hieracium subsimile

Norrl.

736C0992-4267-5C4A-8319-0693F07B918E

Synonym: *Hieraciumreclinatum* (Almq. ex Dahlst.) Johanss.

##### Native status

Native. Forest margins.

##### Distribution

Finland: Al, N; Norway, Sweden (Samuelsson 1954, Tyler 2017). The occurrence in Central Europe is also reported (POWO 2025).

##### Diagnosis

Stems 40–50 cm tall. Rosulate leaves broadly lanceolate, base cuneate, apex triangular, with very small serrate teeth, rigid, bright glaucous-green, nearly glabrous or with rare simple hairs up to 1 cm long and sparse stellate hairs above, on short petioles. Cauline leaves similar, reduced in size, 3–4. Phyllaries triangular, with acute apex, ca. 9 mm long, grey-green, basally with a few or without simple hairs, with very dense glandular hairs 0.5–0.7 mm long and rather dense stellate hairs throughout, apex with numerous short ciliae. Synflorescence branches without simple hairs, with rather dense glandular hairs 0.2–0.3 mm long and stellate hairs. Styles with dark papillae. Ligules glabrous.

### Diaphanoidea-group

#### Hieracium acidodontum

Dahlst. ex Johanss.

FFEDF84E-6CBF-5607-B142-869DBE0B2FBE

##### Native status

Native. Spruce and pine forests.

##### Distribution

Finland: Ab, N, Ta, Oa, Tb, Kb, Om, Ok; Norway (POWO 2025), Sweden (Tyler 2017). The reported occurrence in Eastern Europe (POWO 2025) is inexplicable.

##### Diagnosis

Stems 30–50 cm tall. Rosulate leaves narrowly lanceolate, with small and narrow (serrate) teeth, thin, dark green, with dense simple hairs 1–1.5 mm long above, on rather long petioles. Cauline leaves similar, 2–3. Phyllaries with a narrowly obtuse apex, 10–11 mm long, grey-green, with solitary simple hairs at the base, dense glandular hairs 0.5–1 mm long and sparse stellate hairs in the basal part, apex with few short ciliae. Synflorescence branches with solitary simple hairs, rather dense glandular hairs 0.2–0.3 mm long and stellate hairs. Styles with dark papillae. Ligules glabrous.

#### Hieracium diaphanoides

Lindeb.

C8D61422-C8E9-5643-9F34-84EC77D21ABB

Synonym: *Hieraciumtenebrosum* Norrl.

##### Native status

Native. Pine and spruce forests.

##### Distribution

Finland: Al, Ab, N, Ka, St, Ta, Sa, Oa, Tb, Sb, Kb, Om, Ok; broadly distributed in Northern and Central Europe (Samuelsson 1954, Schljakov 1989, Sennikov 2000, Schou 2001, Sennikov 2003b, Sennikov 2008b, Sennikov and Golubeva 2014, Tyler 2017). Reports from Western and Central Europe, as well as Asia Minor (POWO 2025), may belong to similar, but different taxa.

##### Diagnosis

Stems 40–70 cm tall. Rosulate leaves narrowly to broadly lanceolate, lanceolate-oblong or lanceolate-ovate, base cuneate, apex broadly triangular, with rather numerous minute to small serrate teeth, rather thin, dark-green, with sparse simple hairs 0.7–1.2 mm long above, on rather short petioles. Cauline leaves lanceolate-ovate, 2–4. Phyllaries broadly linear, with a broadly triangular, obtuse apex, 10–11 mm long, blackish-green, without or with a few simple hairs at the base, with abundant glandular hairs 0.8–1.5 mm long, and rare stellate hairs along the margins in the basal half, apex with indistinct ciliae. Synflorescence branches with or without simple hairs, with very dense glandular hairs 0.5–1 mm long and stellate hairs. Styles with black papillae. Ligules glabrous.

#### Hieracium hyalinellum

Brenner

26788B51-862B-54F0-AA3E-EC094DF1D28B

##### Native status

Native. Sparse pine forests.

##### Distribution

Finland: Ab, N, Ta. Endemic to Finland.

##### Diagnosis

Stems 40–50 cm tall. Rosulate leaves lanceolate, base cuneate, apex triangular, with small serrate teeth, thin, grass-green with purple spots, with sparse simple hairs 0.5–0.8 mm long along narrow margins and sparse stellate hairs above, on short petioles. Cauline leaves similar, reduced in size, 2–3. Phyllaries nearly linear, with broadly acute or rather obtuse apex, 9–10 mm long, dark-green, with rare blackish simple hairs ca. 1 mm long, dense to very dense glandular hairs 0.5–1 mm long and sparse stellate hairs mostly along the margins, apex without ciliae. Synflorescence branches with or without rare simple hairs, with rare to sparse glandular hairs 0.2–0.3 mm long and stellate hairs. Styles with black papillae. Ligules glabrous.

#### Hieracium progrediens

Norrl.

1E00E964-8C85-5783-8F76-999D35A9C81D

##### Native status

Native. Birch forests, forest margins, shrubs.

##### Distribution

Tb, Sb, Kb; Sweden (Tyler 2017), northern European Russia (Schljakov 1989).

##### Diagnosis

Stems 50–70 cm tall. Rosulate leaves long-lanceolate, base attenuated to cuneate, apex triangular, with very sparse minute teeth, rather thin, dark-green, with sparse to rather dense simple hairs 1–1.5 mm long above, on rather long petioles. Cauline leaves similar, 2–3. Phyllaries broadly triangular, with acute apex, 11 mm long, blackish-green, usually without simple hairs, with abundant glandular hairs 1–2 mm long and rare stellate hairs along the margins in the basal half, apex with indistinct ciliae. Synflorescence branches without simple hairs, with very dense glandular hairs 0.5–1.2 mm long and stellate hairs. Styles with black papillae. Ligules glabrous.

#### Hieracium silenii

(Norrl.) Norrl.

1E68BF57-3D5E-5734-B2B7-0D108B97A078

##### Native status

Native. Sparse pine forests.

##### Distribution

Ab, N, Ka, St, Ta, Sa, Oa, Tb, Sb, Kb, Ok; northern European Russia (Samuelsson 1954, Schljakov 1989, Sennikov 2000, Sennikov 2008b), Baltic countries (Sennikov 2003b), Belarus (Sennikov 1999b).

##### Diagnosis

Stems 40–50 cm tall. Rosulate leaves lanceolate to oblong-lanceolate, base cuneate to broadly cuneate, apex triangular to subrotund, with minute repand dentation, rigid, grass-green, with simple hairs ca. 0.5 mm long along the margins above, on rather long petioles. Cauline leaves similar, long-petiolate, 2–4. Phyllaries broadly triangular, with broadly acute apex, 8–9(10) mm long, grey-green, with a few short simple hairs at the base, dense glandular hairs 0.5–1(1.2) mm long and sparse stellate hairs throughout, apex with a small coma of numerous or abundant short ciliae. Synflorescence branches with or without solitary simple hairs, with rather dense glandular hairs 0.3–0.5(0.6) mm long and stellate hairs. Styles with black papillae. Ligules glabrous.

#### Hieracium subarctoum

Norrl.

CFD89466-EC71-5A3C-9BFD-E24992B63C42

##### Native status

Native. Birch forests, shrubs, tundra.

##### Distribution

Finland: Oa, Sb, Kb, Om, Ok, Obo, Obu, Ks, Lkk, Lks, Le, Li; Norway, Sweden (Samuelsson 1954, Tyler 2017), northern European Russia (Schljakov 1989, Sennikov 2008b, Sennikov 2009).

##### Diagnosis

Stems 30–50 cm tall. Rosulate leaves broadly lanceolate or oblong-lanceolate, base cuneate, apex broadly triangular, with sparse very small teeth, rather rigid, grass-green, with sparse simple hairs ca. 1 mm long and stellate hairs above, on rather short petioles. Cauline leaves similar, petiolate, 1–3. Phyllaries broadly triangular, with obtuse apex, 8–9 mm long, dark grey-green, without simple hairs, with abundant glandular hairs 0.5–1(1.2) mm long, and stellate hairs rare on the surface and sparse along the margins, apex with a small coma of short ciliae. Synflorescence branches without simple hairs, with dense glandular hairs 0.5–0.7 mm long and stellate hairs. Styles with black papillae. Ligules glabrous.

#### Hieracium subpellucidum

Norrl.

945C7B74-A0BE-5622-86E6-3EE5BFCBA507

##### Native status

Native. Sparse spruce and pine forests and forest margins.

##### Distribution

Finland: Ab, N, Ka, St, Ta, Sa, Oa, Tb, Sb, Kb, Om, Ok, Obo, Obu, Ks, Lkk, Lks, Le; Norway, Sweden (Samuelsson 1954, Tyler 2017), Estonia (Sennikov 2003b), northern and central European Russia (Schljakov 1989, Sennikov 2000, Sennikov 2006b, Sennikov 2008b), extending to Northern Asia as far as Lake Baikal (Chepinoga et al. 2024).

##### Diagnosis

Stems 40–60 cm tall. Rosulate leaves oblong-lanceolate, base cuneate, apex broadly triangular, with small sparse teeth, rather thin, dark-green, with sparse simple hairs 1–1.5 mm long (glabrescent in the centre) and numerous stellate above, on short petioles. Cauline leaves similar, petiolate, usually 1–2 in the basal part. Phyllaries narrowly triangular, with acute apex, 8–9(10) mm long, grey-green, basally sometimes with solitary, but usually without simple hairs, with rather dense to dense glandular hairs 0.6–0.9(1.2) mm long and sparse to rather dense stellate hairs throughout, apex with numerous short ciliae. Synflorescence branches without simple hairs, rather dense glandular hairs 0.3–0.4(0.8) mm long and stellate hairs. Styles with black papillae. Ligules glabrous.

### Vulgata-group

#### Hieracium megavulgatum

T.Tyler

FBCD63E4-FB75-5752-A06E-65D9FBDD2EDB

Synonym: *Hieraciumvulgatiforme* (Dahlst.) Johanss.

##### Native status

Native. Pine and spruce forests, forest margins, shrubs, roadsides.

##### Distribution

Finland: Al, Ab, N, Ka, St, Ta, Sa, Tb, Sb, Kb, Ok; Denmark (Schou 2001), Norway, Sweden (Tyler 2017), northern European Russia (Schljakov 1989). Reported also from Central Europe (POWO 2025).

##### Diagnosis

Stems 40–80 cm tall. Rosulate leaves lanceolate, base cuneate, apex triangular, with sparse serrate teeth, rather thin, but rigid, dark-green, with sparse simple hairs 1 mm long and rare to sparse stellate hairs above, on rather short petioles. Cauline leaves similar, shortly petiolate to sessile, 5–10. Phyllaries triangular, with acute apex, 9–10 mm long, grey-green, with sparse to rather dense black-based simple hairs 1–1.5 mm long, rare to sparse glandular hairs 0.2–0.5(0.7) mm long and sparse stellate hairs on the surface and more prominently along the margins, apex without ciliae. Synflorescence branches with rare to sparse simple hairs, solitary to rare glandular hairs 0.2–0.3 mm long and stellate hairs. Styles with black papillae. Ligules glabrous.

#### Hieracium vulgatum

Fr.

26FE9925-2AA3-5483-A51D-B021309D531E

Synonym: *Hieraciumtriviale* (Norrl.) Norrl.

##### Native status

Native. Pine and spruce forests, forest margins, shrubs, roadsides.

##### Distribution

Al, Ab, N, Ka, St, Ta, Sa, Kl, Oa, Tb, Sb, Kb, Ok, Obo; British Isles (Sell and Murrell 2006, Tyler 2014), Denmark, Norway, Sweden (Samuelsson 1954, Schou 2001, Tyler 2017), Baltic countries (Sennikov 2003b), western and northern Belarus (Sennikov 1999b, Sennikov 1999c) and northern European Russia (Schljakov 1989, Sennikov 2000, Sennikov 2006b, Sennikov 2008b), limited by Tver Region in the south-east (Sennikov 2006c). Apparently present in Central Europe and possibly elsewhere (POWO 2025).

##### Notes

Tyler (2017) rejected the name *Hieraciumvulgatum* in favour of *H.triviale*, following the neotypification of *H.vulgatum* made by Greuter (Greuter and von Raab-Straube 2007).

##### Diagnosis

Stems 40–60 cm tall. Rosulate leaves lanceolate, base narrowly cuneate, apex rather narrowly triangular, with sparse serrate teeth, rather thin, but rigid, mostly dark-green, with sparse to dense simple hairs 0.8–1.2 mm long and with or without rare to sparse stellate hairs above, on rather long petioles. Cauline leaves similar, 3–5. Phyllaries triangular, with acute apex, 8–10 mm long, grey-green, with sparse to dense black-based simple hairs 1(1.5) mm long, rare to rather dense glandular hairs 0.2–0.5(0.7) mm long and sparse to dense appressed stellate hairs on the surface and along a broad stripe on the margins, apex without ciliae. Synflorescence branches with solitary to sparse simple hairs, very rare glandular hairs 0.2–0.3 mm long and stellate hairs. Styles with black papillae. Ligules glabrous.

### Constringentia-group

#### Hieracium brennerianum

Norrl.

62DB20D8-7235-5333-9FAE-7F1BB3DFA6EA

##### Native status

Native. Riversides, tundra.

##### Distribution

Obo, Ks. Possibly endemic to Finland.

##### Notes

This species is inexplicably included in *Hieraciumsparsifolium* Lindeb. in POWO (2025). However, the two species are completely different in their foliage (narrowly oblong to almost linear and sessile in *H.sparsifolium* vs. lanceolate and indistinctly petiolate in *H.brennerianum*), synflorescences (long-branched in *H.sparsifolium* vs. short-branched in *H.brennerianum*) and involucres (phyllaries narrowly triangular to almost oblong in *H.sparsifolium* vs. clearly triangular in *H.brennerianum*). *Hieraciumsparsifolium* was described from Norway and was not reported from Sweden (Johansson 1929).

##### Diagnosis

Stems 35–45 cm tall. Basal leaves usually present at anthesis, lanceolate; cauline leaves 6–8, gradually decreasing from the stem base, lowermost ones lanceolate, base narrowly cuneate or attenuate, apex narrowly triangular, with sparse small teeth, rigid, rather dark green, glabrous, but with sparse to rather dense stellate hairs above, narrowed into indistinct petioles. Phyllaries broadly triangular, with acute apex, ca. 9 mm long, dark grey-green, with rather rare to sparse whitish simple hairs 0.5–1 mm long, rather dense glandular hairs 0.1–0.3 mm long and sparse stellate hairs along the margins, with rather broad glabrous margins, apex with indistinct very short ciliae. Synflorescence branches strongly abbreviated under the central head, with dense simple hairs, very rare to rare glandular hairs 0.1–0.2 mm long and stellate hairs. Styles with black papillae. Ligules glabrous.

#### Hieracium constringens

Norrl.

895FAD9A-D7E0-55CA-B176-7C039725848C

##### Native status

Native. Forest margins, shrubs.

##### Distribution

Finland: Al; Norway, Sweden (Samuelsson 1954, Tyler 2017).

##### Notes

This species is included in *Hieraciumgulldalense* Norrl. in POWO (2025). However, the latter species clearly differs in a much greater number of prominently black-based (vs. nearly white in *H.constringens*) simple hairs on its synflorescence branches.

##### Diagnosis

Stems 30–50 cm tall. Basal leaves always present at anthesis, broadly lanceolate, with small acute teeth, thin, greyish-green, sparsely hairy above (simple hairs 0.7–1 mm long), but glabrous in the centre, on long petioles. Cauline leaves similar, 1–2. Phyllaries triangular, with an acute apex, 9–10 mm long, grey-green, with very dense pale simple hairs 1–1.5 mm long, rare glandular hairs 0.1–0.3 mm long and sparse stellate hairs throughout, apex with numerous short ciliae. Synflorescence branches often strongly abbreviated under the central head, with dense simple hairs, scattered glandular hairs 01–0.2 mm long and stellate hairs; acladium short. Styles with black papillae. Ligules glabrous.

#### Hieracium kuusamoense

Vainio

C652D46A-7BB7-5EA6-B5BF-81596D2B55A3

##### Native status

Native. Tundra, in shrubs and along brooks.

##### Distribution

Finland: Ok, Obu, Ks, Lkk, Lks, Le, Li; northern Scandinavia, northern European Russia (Schljakov 1989, Sennikov 2008b), extending eastwards as far as southern Siberia (Chepinoga et al. 2024). In Asia, the species was reported from the mountains as far as the Tian-Shan and the Himalayas (Sennikov 2012).

##### Notes

This species has been synonymised with *Hieraciumplicatum* Lindeb. (Tyler 2017), but differs from the latter in its rosulate leaves being much more numerous and cauline leaves being largely reduced in size.

##### Diagnosis

Stems 30–40 cm tall. Basal leaves always present at anthesis, narrowly lanceolate to narrowly rhombic-lanceolate, base narrowly cuneate, apex narrowly triangular, with prominent serrate teeth, rigid, dark-green, with sparse to dense simple hairs 0.5–0.8(1) mm and stellate hairs above, on rather short petioles. Cauline leaves similar, shortly petiolate, 2–3. Phyllaries narrowly triangular, with acute apex, (8)9–10 mm long, dark grey-green, with abundant whitish simple hairs 1–1.5(1.8) mm long, sparse glandular hairs 0.1–0.2(0.3) mm long and sparse stellate hairs along the margin, apex with numerous long ciliae. Synflorescence branches strongly abbreviated under the central head, with dense simple hairs, sparse to rather dense glandular hairs 0.1–0.2(0.3) mm long and stellate hairs. Styles with black papillae. Ligules glabrous.

#### Hieracium turbidum

Norrl.

F039E623-F62F-5EAC-88AB-EA483B61F47C

##### Native status

Native. Riverside shrubs and meadows.

##### Distribution

Finland: Ks; northern Russia (Schljakov 1989).

##### Diagnosis

Stems 30–40 cm tall. Basal leaves usually present at anthesis, slightly oblanceolate; cauline leaves 3–4, sparsely spaced, lowermost ones lanceolate, shortly petiolate, middle ones very narrowly rhombic-oblong to indistinctly panduriform, base broadly cuneate, apex triangular, with 5–7 minute narrow teeth in the basal and middle parts, rigid, dark green, lowermost ones with sparse simple hairs ca. 1 mm long, others glabrous, but with dense stellate hairs above. Phyllaries broadly triangular to oblong, with broadly acute apex, 8–9 mm long, blackish-grey-green, with rather dense pale simple hairs 1–1.5 mm long, rare glandular hairs 0.1–0.2 mm long and sparse stellate hairs in the basal part, with rather broad glabrous margins, apex with some short ciliae. Synflorescence branches usually abbreviated under the central head, with sparse pale simple hairs 1–1.5 mm long, rare glandular hairs 0.1–0.2 mm long and stellate hairs, central branch often strongly abbreviated. Styles with black papillae. Ligules glabrous.

### Caesia-group

#### Hieracium caesiomurorum

Lindeb.

5E45CC94-ECD0-5F0F-B7EA-080189083FA7

##### Native status

Native. Pine and spruce forests.

##### Distribution

Finland: Al, Ab, N, St, Ta, Sa, Oa, Tb, Sb, Om; Denmark (Schou 2001), Norway, Sweden (Samuelsson 1954, Tyler 2006e), northern European Russia (Schljakov 1989, Sennikov 2000, Sennikov 2008b), Baltic countries (Sennikov 2003b). Reported also from other parts of Europe (POWO 2025).

##### Diagnosis

Stems 30–50 cm tall. Rosulate leaves broadly lanceolate to lanceolate-ovate, with small to prominent narrow teeth, rather thick, glaucous-green, with sparse simple and stellate hairs above, on long petioles. Cauline leaves similar, 1–2 in the basal half. Phyllaries with a broad apex, 10–11 mm long, grey-green, with rather dense simple hairs 0.5–1 mm long, sparse glandular hairs 0.2–0.5 mm long and stellate hairs mostly along the margins, apex white-margined, with dense and abundant short ciliae and stellate hairs. Synflorescence branches with rare simple hairs, dense glandular hairs 0.2–0.3 mm long and stellate hairs. Styles with black papillae. Ligules glabrous.

#### Hieracium caesium

(Fr.) Fr.

404BD3E6-8C4A-5914-99DA-3EA7933AEC59

Synonym: *Hieraciumbasifolium* (Fr. ex Almq.) Lönnr.

##### Native status

Native. Pine and spruce forests.

##### Distribution

Finland: Al, Ab, N, Ka, St, Ta, Sa, Kl, Oa, Tb, Sb, Kb, Ok, Obo, Ks; Denmark (Schou 2001), Norway, Sweden (Samuelsson 1954, Tyler 2006e), northern European Russia (Schljakov 1989, Sennikov 2000, Sennikov 2008b), Baltic countries (Sennikov 2003b). Reported also elsewhere, but in a different interpretation (POWO 2025).

##### Notes

Sennikov (2005) interpreted the lectotype of *Hieraciumcaesium* as referable to *H.basifolium*, but Tyler (2006e) preferred to exclude the name *H.caesium* from use.

##### Diagnosis

Stems 40–50 cm tall. Rosulate leaves lanceolate to broadly lanceolate, with small to prominent teeth, thick, dark glaucous-green, sometimes with purple spots, usually glabrous above, on long petioles. Cauline leaves similar, 1–3 in the basal half. Phyllaries gradually attenuated to the apex, 10–11 mm long, very dark, with sparse to dense simple hairs 1–1.5 mm long, very rare to rare glandular hairs 0.3–0.4 mm long and sparse stellate hairs throughout, apex with irregular ciliae. Synflorescence branches with solitary to rare simple hairs, very rare glandular hairs 0.2–0.3 mm long and stellate hairs. Styles with black papillae. Ligules glabrous.

#### Hieracium coniops

Norrl.

2E41CF57-61E6-53CD-B901-87C6DC36ADC5

##### Native status

Native. Pine forests, forest margins, shrubs.

##### Distribution

Finland: Ab, N, Ka, St, Ta, Sa, Oa, Ok, Obo, Obu, Ks, Lkk, Lks, Le; Norway, Sweden (Samuelsson 1954, Tyler 2017), northern European Russia (Schljakov 1989, Sennikov 2000, Sennikov 2008b).

##### Notes

This species was treated broadly with the inclusion of *Hieraciumumbricola* (Samuelsson 1954, Schljakov 1989, Sennikov 2000, Sennikov 2008b, Tyler 2017) and the distributions of both taxa are currently unclear due to this confusion.

##### Diagnosis

Stems 25–70 cm tall. Rosulate leaves narrowly lanceolate, often with rather coarse narrow teeth, rather thick, pale glaucous-green, nearly glabrous with numerous stellate hairs above, on short petioles. Cauline leaves similar, (1)2–4(5). Phyllaries narrowly triangular, with an acute apex, 9–10 mm long, pale greyish-green, with sparse to rather dense pale simple hairs 0.8–1(1.3) mm long, rather rare blackish glandular hairs 0.1–0.3(0.5) mm long and sparse to rather dense stellate hairs throughout, apex with few short ciliae. Synflorescence branches without simple hairs, with solitary glandular hairs 0.1–0.2 mm long and stellate hairs. Styles with black papillae. Ligules glabrous.

#### Hieracium coronarium

Brenner

61A075F3-44DC-5261-8FC4-7164EC889590

Synonyms: *Hieraciumadunans* Norrl., *H.diversifolium* Sael. ex Norrl.

##### Native status

Native. Sparse pine and spruce forests, forest margins, shrubs.

##### Distribution

Finland: Al, Ab, N, Ka, St, Ta, Sa, Kl, Oa, Tb, Sb, Kb, Om, Ok, Obo, Ks; Norway, Sweden (Samuelsson 1954, Tyler 2006e), northern European Russia (Schljakov 1989, Sennikov 2000, Sennikov 2008b), Baltic countries (Sennikov 2003b).

##### Diagnosis

Stems 40–50 cm tall. Rosulate leaves lanceolate to elliptic-lanceolate, base cuneate, apex narrowly triangular, with small sparse serrate teeth, rather thick, grey-green, with rare to rather dense simple hairs 1–1.5 mm long and sparse stellate hairs above, on short petioles. Cauline leaves similar, reduced in size, 2–3. Phyllaries triangular or narrowly triangular, with acute apex, 10–11 mm long, dark grey-green, with rare to rather dense blackish simple hairs 0.8–1 mm long, sparse to rather dense blackish glandular hairs 0.4–0.8(1) mm long and sparse stellate hairs along the margins, apex without ciliae. Synflorescence branches with very rare simple hairs, rare to rather dense glandular hairs 0.3–0.5 mm long and stellate hairs. Styles with dark papillae. Ligules glabrous.

#### Hieracium laeticolor

(Almq.) Lönnr.

8BE025A5-3870-550E-A19A-FDDA6B8F26CA

Synonym: *Hieraciumprolixiforme* Norrl.

##### Native status

Native. Sparse pine forests, forest margins.

##### Distribution

Finland: Ab, N, Ka, St, Ta, Sa, Oa, Tb, Sb, Kb, Om, Ok, Obu, Ks; Scandinavia (Samuelsson 1954, Tyler 2006e), northern European Russia (Schljakov 1989, Sennikov 2000, Sennikov 2008b), Baltic countries (Sennikov 2003b). Reported also from other parts of Europe (POWO 2025).

##### Diagnosis

Stems 30–40 cm tall. Rosulate leaves narrowly lanceolate, base cuneate, apex narrowly triangular, with minute or small teeth, thick, glaucous-green, glabrous or with a few short simple hairs along the margins above, on short petioles. Cauline leaves similar, usually much reduced in size, 1–2(3). Phyllaries narrowly triangular, with acute apex, ca. 9 mm long, grey-green, with rather dense black-based simple hairs 0.5–1 mm long, rather rare to sparse glandular hairs 0.1–0.3 mm long and sparse to rather dense stellate hairs throughout, apex with a few ciliae. Synflorescence branches with rather dense simple hairs, rare to sparse glandular hairs 0.1–0.3 mm long and stellate hairs. Styles with black papillae. Ligules glabrous.

#### Hieracium longimanum

(Norrl.) Dahlst.

6F0AFA2B-75F2-52D9-AE7E-DB0AE4ACEA88

##### Native status

Native. Forest margins, shrubs.

##### Distribution

Finland: Al, Ab, N; Sweden (Tyler 2006e).

##### Diagnosis

Stems 50–60 cm tall. Rosulate leaves lanceolate to ovate-lanceolate, base broadly cuneate, apex triangular, with small to coarse remote teeth, thick, bright glaucous-green, glabrous above, on rather long petioles. Cauline leaves similar, mostly basal, petiolate, 1–2. Phyllaries triangular, with acute apex, 12–13 mm long, grey-green, with sparse to rather dense half-dark simple hairs 1–1.5 mm long, rather dense glandular hairs 0.3–0.5(0.7) mm long and sparse stellate hairs mostly along the margins, apex with few ciliae. Synflorescence branches with solitary simple hairs, rather rare glandular hairs 0.2–0.4 mm long and stellate hairs. Styles with black papillae. Ligules glabrous.

#### Hieracium plumbeum

Blytt & Fr.

FB433673-4D98-502C-BC0F-101296D5C59B

##### Native status

Native. Pine forests, forested rocks.

##### Distribution

Finland: Al, Ab, N, Ka, St, Ta, Sa, Oa, Sb, Obu, Ks; Denmark (Schou 2001), Norway, Sweden (Samuelsson 1954, Tyler 2006e), Baltic countries (Sennikov 2003b), northern European Russia (Schljakov 1989, Sennikov 2000, Sennikov 2008b). Reported also elsewhere in Europe (POWO 2025).

##### Notes

Some authors used the name *Hieraciumcaesium* for this species (Samuelsson 1954, Schljakov 1989, Schou 2001).

##### Diagnosis

Stems 30–50 cm tall. Rosulate leaves lanceolate to broadly lanceolate, with minute to prominent broad teeth, thick, plumbeous, sometimes with purple spots, glabrous above, on long petioles. Cauline leaves similar, 1–2 in the basal half. Phyllaries attenuated to the very narrow apex, 10–12 mm long, blackish, with sparse black-based flexuous simple hairs 1.5–2 mm long, rare glandular hairs 0.1–0.3 mm long and sparse stellate hairs throughout, apex without ciliae. Synflorescence branches with solitary to rare simple hairs, solitary to rare glandular hairs 0.1–0.3 mm long and stellate hairs. Styles with dark papillae. Ligules glabrous.

#### Hieracium ravidum

Brenner

253872FA-7E63-5A56-A5A3-AD078A5BC657

Synonym: *Hieraciumgalbanum* (Dahlst.) Brenner

##### Native status

Native. Sparse pine forests.

##### Distribution

Finland: Al, Ab, N, Ka, St, Ta, Sa, Kl, Oa, Tb, Sb, Kb, Ok, Obo, Obu; Norway, Sweden (Samuelsson 1954, Tyler 2006e), Baltic countries (Sennikov 2003b), northern European Russia (Schljakov 1989, Sennikov 2000, Sennikov 2008b, Sennikov 2009).

##### Diagnosis

Stems 30–50 cm tall. Rosulate leaves broadly lanceolate, oblong-lanceolate or lanceolate-ovate, base broadly cuneate, subrotund or nearly truncate, apex narrowly to broadly triangular, with distant, narrowly acute, small or coarse teeth, thick, greyish-glaucous-green, with or without rare simple hairs above, on rather short petioles. Cauline leaves lanceolate-ovate, 1–2. Phyllaries broadly triangular, with rather obtuse apex, 10–12 mm long, grey-green, with sparse to rather dense pale to blackish simple hairs 1–1.5 mm long, rare glandular hairs 0.1–0.3 mm long and sparse to rather dense stellate hairs mostly along the margins, apex with few ciliae. Synflorescence branches with rare simple hairs, solitary to rare glandular hairs 0.2–0.3 mm long and stellate hairs. Styles with black papillae. Ligules glabrous.

#### Hieracium umbricola

Saelan ex Norrl.

ED903652-6078-5B94-AF60-0B616389DE05

##### Native status

Native. Pine forests, forest margins, shrubs.

##### Distribution

Finland: Al, Ab, N, Ka, St, Ta, Sa, Oa; Norway, Sweden (Samuelsson 1954), northern European Russia (Schljakov 1989, Sennikov 2000) as far eastwards as Kostroma Region (Sennikov and Golubeva 2014).

##### Notes

The distribution in Scandinavia may be incorrect due to the ongoing confusion with *Hieraciumconiops* (Samuelsson 1954, Tyler 2017).

##### Diagnosis

Stems 30–60 cm tall. Rosulate leaves lanceolate, oblong-lanceolate to ovate-lanceolate, base cuneate, apex triangular to narrowly triangular, with sparse small acute teeth, rather thick, dark glaucous-green, usually without simple hairs, but with sparse long-armed stellate hairs above, on rather short petioles. Cauline leaves similar, 1–2. Phyllaries triangular, with acute apex, 10–11 mm long, dark or blackish-grey-green, with rather dense, nearly half-black simple hairs 0.8–1.2(1.5) mm long, rather rare black glandular hairs 0.1–0.3(0.5) mm long and rare to rather dense stellate hairs throughout, apex with sparse short ciliae. Synflorescence branches with solitary to very rare simple hairs, solitary to very rare glandular hairs 0.2–0.3 mm long and stellate hairs. Styles with black papillae. Ligules glabrous.

#### Hieracium wainioi

Norrl.

8EDA9EAB-7A37-5EAF-B25E-40F54E93173B

##### Native status

Native. Shrubs, riversides.

##### Distribution

Finland: Obu, Lks; northern European Russia (Schljakov 1989).

##### Diagnosis

Stems 40–50 cm tall. Rosulate leaves lanceolate, base cuneate, apex triangular, with prominent narrow teeth, rigid, greyish-green, with sparse simple hairs above (glabrescent in the middle), on long petioles. Cauline leaves lanceolate to rhombic-lanceolate, 4–7. Phyllaries broadly triangular, with acute apex, ca. 9 mm long, grey-green, with sparse black-based simple hairs up to 1 mm long, rare to sparse glandular hairs 0.1–0.3(0.5) mm long and rather dense stellate hairs along the surface, apex with few ciliae. Synflorescence branches with sparse simple hairs under the central head, rare to rather dense glandular hairs 0.2–0.3(0.5) mm long and stellate hairs. Styles with dark or discoloured papillae. Ligules glabrous.

### Fulvescentia-group

#### Hieracium fulvescens

Norrl.

940244B6-A915-587D-BA75-292D59D1219A

##### Native status

Native. Sparse pine forests.

##### Distribution

Finland: Ab, N, Ka, St, Ta, Sa, Oa, Tb, Sb, Kb, Om, Obo; Norway, Sweden (Samuelsson 1954, Tyler 2006e), Baltic countries (Sennikov 2003b), northern European Russia (Schljakov 1989, Sennikov 2000, Sennikov 2008b), Asian Russia, reaching as far as Lake Baikal in Siberia (Chepinoga et al. 2024).

##### Diagnosis

Stems 40–50 cm tall. Rosulate leaves lanceolate or rhombic-lanceolate, base cuneate, apex triangular to broadly triangular, with small to prominent coarse teeth, rather thick, bright-green, with sparse to rather dense simple hairs 0.5–0.8 mm long along the margins and nearly without stellate hairs above, on long petioles. Cauline leaves similar, much reduced in size, 2–3. Phyllaries broadly triangular, with rather acute apex, 10–11 mm long, pale-green, with or without solitary simple hairs, with dense glandular hairs 0.5–0.8 mm long and sparse to rather dense stellate hairs throughout, apex with few short ciliae. Synflorescence branches usually without simple hairs, with solitary or very rare glandular hairs 0.3–0.5 mm long and stellate hairs. Styles with black papillae. Ligules glabrous.

#### Hieracium lucens

Norrl.

3A4C307F-375F-5CEA-9B63-0F54AD24ACBE

##### Native status

Native. Sparse pine forests.

##### Distribution

Finland: Ab, N, St, Ta, Sa, Oa, Tb, Sb, Om, Ok, Obo, Obu, Ks; seemingly Scandinavia and northern European Russia, Asian Russia eastwards up to Sakhalin Island (Chepinoga et al. 2024).

##### Notes

This species was included in *Hieraciumfulvescens* by Schljakov (1989), but appeared re-described under several names from Asian Russia (Chepinoga et al. 2024).

##### Diagnosis

Stems 40–50 cm tall. Rosulate leaves narrowly lanceolate to lanceolate, base narrowly cuneate, apex triangular, with small serrate teeth, rather thick, grass-green, with sparse simple hairs 0.5–1 mm long and sparse to rather dense stellate hairs above, on short petioles. Cauline leaves similar, ca. 3. Phyllaries broadly triangular, with rather acute apex, 10–11 mm long, grey-green, with or without a few simple hairs at the base, with rather dense to dense glandular hairs 0.5–0.8(1) mm long and sparse to rather dense stellate hairs throughout, apex with few short ciliae. Synflorescence branches without simple hairs, with sparse glandular hairs 0.3–0.5 mm long and stellate hairs. Styles with slightly dark (yellowish) papillae. Ligules glabrous.

#### Hieracium porrigens

Almq. ex Elfstr.

8A486794-ACB4-516E-9AE3-BD4DEC6256BB

##### Native status

Native. Forest margins.

##### Distribution

Finland: Al; Scandinavia (Tyler 2006e), Baltic countries (Sennikov 2003b). As the species is not present in Finland east to the Åland Islands, its report from the neighbouring Russia (Schljakov 1989) is unlikely correct.

##### Diagnosis

Stems 40–50 cm tall. Rosulate leaves lanceolate, rhombic-lanceolate or lanceolate-ovate, base broadly cuneate, apex triangular, with numerous coarse teeth, rather thick, with sparse simple hairs 1–1.5 mm long along the margins above, on rather long petioles. Cauline leaves similar, reduced in size, 2–3. Phyllaries broadly triangular, with broadly acute apex, 11(12) mm long, pale grey-green, with very rare black-based simple hairs 1–1.5 mm long, rather dense glandular hairs 0.5–0.8(1) mm long and rather dense stellate hairs throughout, apex with a large coma of abundant ciliae. Synflorescence branches with rare simple hairs, dense glandular hairs 0.2–0.4(0.5) mm long and stellate hairs. Styles with dark papillae. Ligules glabrous.

### Bifida-group

#### Hieracium acidotum

Dahlst.

13CD1D18-028D-57AB-9E6F-38EB8B5BE4AA

##### Native status

Native. Sparse pine forests.

##### Distribution

Finland: Ab, N, Ta, Sa; Norway, Sweden (Tyler 2006e).

##### Notes

This species is also reported from Germany (Gottschlich 2024), apparently in an expanded circumscription.

##### Diagnosis

Stems 30–50 cm tall. Rosulate leaves elliptic or elliptic-ovate, base broadly cuneate to truncate, apex subrotund to broadly triangular, with prominent large teeth, rather thin, glaucous-green, glabrous above, on long petioles. Cauline leaves similar, single at the stem base. Phyllaries linear-triangular, with a narrowly attenuated, subulate apex, 9–10 mm long, grey-green, with rather dense simple hairs 1–1.5 mm long, sparse glandular hairs 0.2–0.4 mm long and sparse stellate hairs at the base, apex without ciliae. Synflorescence branches with sparse simple hairs, rare glandular hairs 0.1–0.3 mm long and stellate hairs. Styles with dark papillae. Ligules glabrous.

#### Hieracium caesiiflorum

Almq. ex Norrl.

9371A662-68C7-5F63-8AAE-399CC90B2508

##### Native status

Native. Sparse pine forests, forest margins, tundra.

##### Distribution

Finland: Al, Ab, N, Ka, St, Ta, Sa, Kl, Oa, Tb, Sb, Kb, Om, Ok, Obu, Ks, Lkk, Le, Li; Norway, Sweden (Samuelsson 1954, Tyler 2006e), northern European Russia (Schljakov 1989, Sennikov 2000, Sennikov 2008b). Reported also as broadly distributed in Central Europe (POWO 2025).

##### Notes

Hackman and Sennikov (1998) treated *Hieraciumcaesiiflorum* as a synonym of *H.subcaesium* (Fr.) Lindeb., but the latter species name has not been nomenclaturally assessed and its true identity has not been established.

##### Diagnosis

Stems 25–50 cm tall. Rosulate leaves ovate or elliptic-ovate, base sagittate or truncate, apex broadly triangular, with prominent large teeth, rather thick, glaucous-green, usually glabrous above, on long petioles. Cauline leaves similar, single at the stem base. Phyllaries with a broadly acute apex, 9–10 mm long, pale, with sparse to rather dense whitish simple hairs 0.5–0.8 mm long, very rare glandular hairs 0.1–0.3 mm long and abundant stellate hairs throughout, apex without ciliae. Synflorescence branches with sparse simple hairs, solitary glandular hairs 0.2–0.3 mm long and stellate hairs. Styles with dark papillae. Ligules glabrous.

#### Hieracium caesitium

(Norrl.) Brenner

6D8003FB-CEB0-55C2-9CF1-FB5004AF6425

Synonym: *Hieraciummaculosum* (Dahlst. ex Stenstr.) Dahlst.

##### Native status

Native. Forest margins, sparse pine forests.

##### Distribution

Finland: Al, Ab, N, Ka, St, Ta, Sa, Oa, Tb, Sb, Kb, Ok, Obo, Ks, Lks; Norway, Sweden (Samuelsson 1954), northern European Russia (Schljakov 1989, Sennikov 2006c, Sennikov 2008b). Reported also from Western Europe (POWO 2025).

##### Diagnosis

Stems 40–50 cm tall. Rosulate leaves elliptic-ovate to triangular-ovate, base truncate to sagittate, apex rather acute, with prominent coarse teeth at the base, rather thick, bright-green, glabrous or with sparse simple hairs along the margins above, on rather long petioles. Cauline leaf single, reduced in size or absent. Phyllaries narrowly triangular, with a narrowly acute apex, 9–10 mm long, grey-green, with sparse to rather dense dark simple hairs 0.5–0.8(1) mm long, rare glandular hairs 0.2–0.4(0.5) mm long and sparse stellate hairs throughout, apex with some long ciliae. Synflorescence branches with rare to sparse simple hairs up to 0.5 mm long, sparse glandular hairs 0.2–0.3 mm long and stellate hairs. Styles with black papillae. Ligules glabrous.

#### Hieracium chlorellum

Norrl.

BA76F3E7-CBC6-5A0C-AE51-8CFAB9EBBBF2

##### Native status

Native. Sparse pine forests.

##### Distribution

Finland: Al, Ab, N, Ka, Ta, Sa, Kl, Sb, Kb; Denmark (Schou 2001), Norway, Sweden (Samuelsson 1954, Tyler 2006e), northern European Russia (Samuelsson 1954, Schljakov 1989, Sennikov 2000, Sennikov 2008b) reaching as far as Tver Region in the east (Sennikov 2006b).

##### Diagnosis

Stems 30–40 cm tall. Rosulate leaves triangular-ovate or ovate, base sagittate, apex broadly triangular or usually subrotund, with large triangular teeth in the basal half, rather thick, pale-green, with rare simple hairs 0.6–0.8 mm long above (glabrescent in the centre), on short petioles. Cauline leaf single, usually reduced. Phyllaries broadly triangular, with a broadly acute apex, 10–11 mm long, pale grey-green, with sparse to rather dense whitish simple hairs 0.5–0.8(1) mm long, sparse to rather dense glandular hairs 0.3–0.5(0.7) mm long and dense stellate hairs throughout, apex with long ciliae. Synflorescence branches with solitary simple hairs, rare to sparse glandular hairs 0.3–0.5 mm long and stellate hairs. Styles with dark papillae. Ligules glabrous.

#### Hieracium crispulum

Norrl.

58B5CBEB-9E10-5277-8E34-F66CBF1BB225

##### Native status

Native. Sparse pine forests.

##### Distribution

Finland: Ab, N, Ka, Ta, Sa, Oa; northern European Russia (Schljakov 1989, Sennikov 2000, Sennikov 2008b).

##### Diagnosis

Stems 40–50 cm tall. Rosulate leaves elliptic to elliptic-ovate, with large coarse teeth, rather thin, glaucous-green, nearly glabrous above, on long petioles. Cauline leaves usually reduced. Phyllaries linear-triangular, with a narrowly attenuated apex, 9–11 mm long, grey-green, with rather dense pale simple hairs 1–1.5 mm long, sparse glandular hairs 0.1–0.3 mm long and sparse stellate hairs in the basal half, apex without ciliae. Synflorescence branches with sparse simple hairs, rare to sparse glandular hairs 0.1–0.2 mm long and stellate hairs. Styles with black papillae. Ligules glabrous.

#### Hieracium multifrons

Brenner

B0148A08-3ABF-51A0-95BB-CF839041865F

##### Native status

Native. Herb-rich pine forests.

##### Distribution

Finland: Ab, N, St, Ta; northern European Russia (Schljakov 1989, Sennikov 2000), Estonia (Sennikov 2003b).

##### Diagnosis

Stems 40–50 cm tall. Rosulate leaves oblong-ovate or ovate, base truncate to cordate, apex triangular, with irregular coarse broad teeth, rather thin, dark-green, with rare to sparse simple hairs 0.3–0.5 mm long above, on long petioles. Cauline leaf single, rather large, elliptic-ovate or triangular-ovate. Phyllaries narrowly triangular, with a narrowly acute apex, 9–11 mm long, grey-green, with rather dense to dense simple hairs 0.5–0.7(1) mm long, sparse to rather dense glandular hairs 0.3–0.5(0.7) mm long and sparse stellate hairs along the margins, apex with conspicuous long ciliae. Synflorescence branches with rare simple hairs, rare to sparse glandular hairs 0.2–0.4 mm long and stellate hairs. Styles with black papillae. Ligules glabrous.

#### Hieracium niviferum

Norrl.

11730276-06CC-5DC6-A2A2-314C4C98AD96

##### Native status

Native. Forest margins.

##### Distribution

Finland: Sb, Kb, Ok. Endemic to Finland.

##### Diagnosis

Stems 50–60 cm tall. Rosulate leaves elliptic to lanceolate-elliptic, with minute teeth or nearly entire margins, rather thick, glaucous-green, glabrous or with few simple hairs along margins above, on long petioles. Cauline leaf single, sometimes reduced. Phyllaries linear-triangular, with a narrowly attenuated, almost subulate apex, ca. 11 mm long, pale grey-green, with sparse to rather dense, half-black simple hairs 1–1.5 mm long, solitary to very rare glandular hairs 0.2–0.3 mm long and dense stellate hairs over the surface, apex with a thin, but considerable ornamentation of ciliae and stellate hairs. Synflorescence branches with rare to sparse dark simple hairs, very rare to sparse glandular hairs 0.2–0.3 mm long and stellate hairs. Styles with black papillae. Ligules glabrous.

#### Hieracium pendulum

(Dahlst.) Dahlst.

28D3DF6E-A11D-50D7-A3CD-DFC24899D278

##### Native status

Native. Shrubs and herbs in tundra and along streams.

##### Distribution

Finand: Ks, Lkk, Lks, Le; Norway, Sweden (Samuelsson 1954, Tyler 2006e), northern European Russia (Schljakov 1989, Sennikov 2008b).

##### Diagnosis

Stems 30–40 cm tall. Rosulate leaves lanceolate-oblong, base cuneate, apex acute, with numerous small acute teeth, rather thin, slightly greyish-green, with sparse simple hairs 0.5–0.8 mm long along the margins and stellate hairs along the central vein above, on short petioles. Cauline leaf reduced or single at the stem base. Phyllaries narrowly attenuated, 9–10 mm long, grey-green, with sparse simple hairs 0.5–0.8(1) mm long, very rare glandular hairs 0.2–0.3 mm long and abundant stellate hairs throughout, apex with numerous short ciliae. Synflorescence branches usually without simple and glandular hairs, with stellate hairs. Styles with black papillae. Ligules glabrous.

#### Hieracium prolixum

Norrl.

3A0ABDEF-5C13-50F2-8AF8-0BE8D874FA6B

##### Native status

Native. Forest margins, sparse pine forests.

##### Distribution

Finland: Al; Denmark (Schou 2001), Norway, Sweden (Samuelsson 1954, Tyler 2006e), northern European Russia (Schljakov 1989), Baltic countries (Sennikov 2003b), Belarus (Sennikov 1999b). Reported from other parts of Europe (POWO 2025).

##### Diagnosis

Stems 40–50 cm tall. Rosulate leaves elliptic-ovate to triangular-ovate, base truncate to sagittate, apex rather acute, with prominent coarse teeth at the base, rather thick, bright-green, glabrous or with sparse simple hairs along the margins above, on rather short petioles. Cauline leaf single, reduced in size. Phyllaries narrowly triangular, with an acute apex, 9–11 mm long, grey-green, with rare to sparse dark simple hairs 0.5–0.8(1) mm long, sparse to rather dense glandular hairs 0.3–0.6(0.8) mm long and stellate hairs sparse in the basal part and numerous along the margins, apex with a few long ciliae. Synflorescence branches with solitary simple hairs, rare glandular hairs 0.2–0.4 mm long and stellate hairs. Styles with black papillae. Ligules glabrous.

#### Hieracium stenolepis

Lindeb.

FCB88006-B46B-5D35-873E-787C65B1F54F

##### Native status

Native. Sparse pine forests.

##### Distribution

Finland: Ab; Denmark (Schou 2001), Norway, Sweden (Samuelsson 1954, Tyler 2006e), Estonia (Sennikov 2003b). Reported from other parts of Europe (POWO 2025).

##### Diagnosis

Stems 30–40 cm tall. Rosulate leaves elliptic to lanceolate-elliptic, with coarse teeth in the basal part, rather thick, glaucous-green, glabrous above, on long petioles. Cauline leaf usually reduced. Phyllaries linear-triangular, with a narrowly attenuated, subulate apex, 10 mm long, grey-green, with sparse to rather dense, mostly pale simple hairs 0.8–1.2(1.5) mm long, very rare glandular hairs 0.2–0.3 mm long and dense stellate hairs over the surface, apex without ciliae. Synflorescence branches with rare simple hairs, very rare glandular hairs 0.1–0.2 mm long and stellate hairs. Styles with dark papillae. Ligules glabrous.

#### Hieracium subholophyllum

Brenner

463397DD-95F1-52CA-BCAC-BFE006A4B540

Synonym: *Hieraciumhololoma* Brenner

##### Native status

Native. Open places in spruce forests.

##### Distribution

Finland: Ab, N, Ta; north-western European Russia (Sennikov 2000), Estonia (Schljakov 1989, Sennikov 2003b).

##### Diagnosis

Stems 40–50 cm tall. Rosulate leaves oblong, base broadly cuneate to subrotund, apex broadly triangular to subrotund, with small distant teeth, rather thick, greyish-green, with rare to sparse simple hairs 0.5–0.6 mm long above, on long petioles. Cauline leaf single, ovate to lanceolate-ovate. Phyllaries narrowly triangular, with an acute apex, 10–11 mm long, pale grey-green, with sparse to rather dense whitish simple hairs 0.8 mm long, rather dense to dense black glandular hairs 0.3–0.5 mm long and sparse to dense stellate hairs throughout, apex with few long ciliae. Synflorescence branches with solitary simple hairs, solitary to sparse glandular hairs 0.2–0.3 mm long and stellate hairs. Styles with black papillae. Ligules glabrous.

#### Hieracium triangulare

(Almq.) Norrl.

682429D3-9DDA-54A9-97F1-A20CB30F12C8

##### Native status

Native. Forest margins.

##### Distribution

Finland: Al, Ab, N, Ka, St, Ta, Oa; Norway, Sweden (Samuelsson 1954, Tyler 2006e), north-western European Russia (Schljakov 1989), Estonia (Sennikov 2003b).

##### Notes

This species was placed to the Murorum-group by Tyler (2006e), although its leaves and indumentum are closer to the Bifida-group. It may deserve a placement into a separate group due to its isolated position (e.g. Schljakov (1989), Tyler (2006e)).

##### Diagnosis

Stems 50–60 cm tall. Rosulate leaves triangular-ovate or oblong-ovate, with large coarse teeth, rather thick, dark-green, with sparse simple hairs 0.5–0.8 mm above, on rather long petioles. Cauline leaves similar, often reduced. Phyllaries narrowly triangular, with an acute apex, 8–9 mm long, grey-green, with sparse simple hairs 0.5–0.8 mm long, rather dense glandular hairs 0.2–0.6(0.8) mm long and sparse stellate hairs, apical part with abundant stellate hairs and long ciliae. Synflorescence branches with solitary simple hairs, dense glandular hairs 0.3–0.4 mm long and stellate hairs. Styles with black papillae. Ligules glabrous.

### Murorum-group

#### Hieracium altipes

(Zahn) Dahlst.

7CCD787D-5182-5ECB-A002-A54738C3B2AC

##### Native status

Native. Sparse forests, open forest margins.

##### Distribution

Finland: Al; Sweden (Tyler 2006e). Erroneously reported from north-western European Russia by Schljakov (1989).

##### Diagnosis

Stems 40–50 cm tall. Rosulate leaves elliptic or lanceolate-elliptic, base narrowly cuneate or cuneate, apex triangular or subrotund, with small repand teeth, thin, pale-green, with sparse simple hairs 0.5 mm long above, broadly glabrescent in the centre, on long petioles. Cauline leaf single, reduced in size. Phyllaries narrowly triangular, with acute apex, 10–11 mm long, grey-green, with rare black simple hairs 1–1.5 mm long, rather dense glandular hairs 0.8–1.5 mm long and sparse stellate hairs along the margins, apex without ciliae. Synflorescence branches with solitary simple hairs, very dense glandular hairs 0.5–0.8 mm long and stellate hairs. Styles with black papillae. Ligules glabrous.

#### Hieracium canipes

(Almq. ex Stenstr.) Dahlst.

DD44D24F-66DD-5672-A252-EB0FC8A675ED

##### Native status

Native. Sparse forests, open forest margins.

##### Distribution

Finland: Al; Norway, Sweden (Samuelsson 1954). Records from north-western European Russia (Sennikov 2000) belong to another taxon.

##### Diagnosis

Stems 40–60 cm tall. Rosulate leaves elliptic or lanceolate-elliptic, base cuneate, apex broadly triangular or subrotund, with small teeth at the base, thin, pale-green, with sparse simple hairs 0.5–0.8 mm long above, glabrescent in the middle, on long petioles. Cauline leaf single, usually reduced. Phyllaries narrowly triangular, with acute apex, 10–11 mm long, grey-green, with rare black simple hairs 0.8–1 mm long, rather dense glandular hairs 0.5–1 mm long and rather dense stellate hairs throughout, apex with indistinct ciliae. Synflorescence branches with solitary simple hairs, rather dense glandular hairs 0.5–0.7 mm long and stellate hairs. Styles with black papillae. Ligules glabrous.

#### Hieracium chloromaurum

Johanss.

F4182E59-3A25-5347-9CDE-C400E5673C32

##### Native status

Native. Sparse spruce and pine forests, forest margins.

##### Distribution

Finland: Ab, N, Ka, St, Ta, Oa, Tb, Sb, Kb, Om, Ok, Ks; Norway, Sweden (Samuelsson 1954), northern European Russia (Schljakov 1989, Sennikov 2000, Sennikov 2008b), Estonia (Sennikov 2003b).

##### Diagnosis

Stems 40–50 cm tall. Rosulate leaves oblong-elliptic to lanceolate-elliptic, base subrotund, broadly cuneate to cuneate, apex obtuse, with indistinct teeth, thin, grass-green, with rather dense to dense simple hairs 0.3–0.5(0.8) mm long above, on rather long petioles. Cauline leaf 1 (rarely 2), similar, reduced in size. Phyllaries narrowly triangular, with acute apex, 10–11 mm long, dark-green, without simple hairs, with abundant slender glandular hairs 0.8–1.5(1.8) mm long and sparse stellate hairs restricted to the narrow margin, apex mostly without ciliae. Synflorescence branches without simple hairs, with dense to abundant glandular hairs 0.5–0.8(1.1) mm long and stellate hairs. Styles with black papillae. Ligules glabrous.

#### Hieracium ciliatiflorum

Pugsley

9E176F24-0B61-5791-A371-41281200493B

Synonym: *Hieraciumciliatum* (Almq.) Dahlst.

##### Native status

Native. Forest margins.

##### Distribution

Finland: Al; Denmark (Schou 2001), Sweden (Samuelsson 1954).

##### Diagnosis

Stems 30–40 cm tall. Rosulate leaves oblong-lanceolate to oblong-ovate, base broadly cuneate to truncate, apex triangular to subrotund, with smal serrate teeth, thin, dark-green, with sparse to rather dense simple hairs 1–2.5 mm long above, on long petioles. Cauline leaf single, often reduced. Phyllaries triangular, with a broad apex, 9–10 mm long, grey-green, with rare to sparse blackish simple hairs 1–1.5 mm long, abundant glandular hairs 0.5–1 mm long and sparse stellate hairs mostly along the margins, apex with abundant ciliae. Synflorescence branches with solitary simple hairs, abundant glandular hairs 0.4–0.7 mm long and stellate hairs. Styles with nearly yellow papillae. Ligules ciliate.

#### Hieracium diminuens

(Norrl.) Norrl.

5F3FE228-83D2-541B-90A9-DF0E73756592

##### Native status

Native. Forest margins.

##### Distribution

Finland: Tb, Sb, Kb, Ok, Obu, Ks, Lkk, Lks, Le, Li; Norway, Sweden (Samuelsson 1954), northern European Russia (Schljakov 1989, Sennikov 2008b).

##### Diagnosis

Stems 30–45 cm tall. Rosulate leaves elliptic to elliptic-ovate, base broadly cuneate, truncate or sagittate, apex broadly triangular, with minute or small teeth in the basal part, thin, green, with sparse simple hairs 0.5 mm long above (broadly glabrescent in the centre), on long petioles. Cauline leaf single, ovate-lanceolate, single. Phyllaries with narrowly acute apex, 10–11 mm long, grey-green, without simple hairs, with dense glandular hairs 0.5–1 mm long and rather dense stellate hairs throughout, apex with abundant short ciliae. Synflorescence branches without simple hairs, with rather dense glandular hairs 0.4–0.5 mm long and stellate hairs. Styles with black papillae. Ligules glabrous.

#### Hieracium dispansiforme

Norrl.

9EA48DC4-8C34-5C13-8C49-74C4434E5FC3

##### Native status

Native. Pine forests.

##### Distribution

Finland: Ab, N, Ka, St, Ta, Oa, Tb, Sb, Kb, Ok, Obu; northern European Russia (Sennikov 2006c).

##### Diagnosis

Stems 30–40 cm tall. Rosulate leaves oblong to oblong-lanceolate, base cuneate, apex obtuse, broadly triangular to subrotund, with small distant teeth, thin, dark-green, with rather dense simple hairs 0.5–1 mm long above, on long petioles. Cauline leaf single, reduced in size. Phyllaries narrowly triangular, with acute apex, 10–11 mm long, dark grey-green, with very rare to rare blackish simple hairs 0.5–0.8 mm long, abundant black glandular hairs 0.6–0.9(1) mm long and very sparse stellate hairs along the margins, apex without ciliae. Synflorescence branches without simple hairs, with dense glandular hairs 0.3–0.5 mm long and stellate hairs. Styles with black papillae. Ligules glabrous.

#### Hieracium distractum

Norrl.

F1D06F74-EB6F-541E-99AB-6F46A9A5D65A

##### Native status

Native. Sparse pine and spruce forests.

##### Distribution

Finland: Ab, N, Ka, St, Ta, Sa, Tb, Sb, Kb, Obu, Ks; northern Sweden (Tyler 2006e), Baltic countries (Sennikov 2003b), north-western Belarus (Sennikov 1999c), northern European Russia (Schljakov 1989, Sennikov 2000, Sennikov 2008b).

##### Diagnosis

Stems 30–50 cm tall. Rosulate leaves broadly elliptic to elliptic-ovate, with small or prominent narrow teeth, thin, grass-green, with sparse to dense simple hairs 0.5 mm long above, on rather long petioles. Cauline leaf single, often reduced. Phyllaries narrowly triangular, with acute apex, 9–10 mm long, grey-green, without simple hairs, with very dense to abundant glandular hairs 0.6–1(1.5) mm long and sparse stellate hairs mostly along the margins, apex with a few ciliae. Synflorescence branches without simple hairs, with dense glandular hairs 0.5–0.6 mm long and stellate hairs. Styles with black papillae. Ligules glabrous.

#### Hieracium fenno-orbicans

Norrl.

FC0B301E-C0A0-5B24-B657-9C617B389C2E

Synonym: *Hieraciumdistendens* Brenner

##### Native status

Native. Open places in pine and spruce forests.

##### Distribution

Finland: Ab, N, Ka, St, Ta, Kl, Oa, Tb, Sb, Kb, Obo, Obu, Ks; northern European Russia (Samuelsson 1954, Schljakov 1989, Sennikov 2000, Sennikov 2008b).

##### Notes

According to Sennikov (2002a), the name *Hieraciumfenno-orbicans* Norrl. was validly published in Lindberg (1906), whereas *H.distendens* Brenner was described in Brenner (1906). Both works are minor notes, which appeared in the same volume of *Memoranda Societatis pro Fauna et Flora Fennica*. Both have been originally issued as preprints, which effectively established their priority. Lindberg's correspondence was typeset before Brenner's note and, therefore, likely has been printed and distributed earlier and has priority over the latter. The reverse synonymy accepted by Hackman and Sennikov (1998) has no standing.

##### Diagnosis

Stems 40–50 cm tall. Rosulate leaves oblong to broadly oblong, base broadly cuneate, subrotund or truncate, apex subrotund, with numerous serrate teeth, thin, grass-green, with sparse simple hairs 0.4–0.5 mm long above, on long petioles. Cauline leaf single, triangular. Phyllaries triangular, with acute apex, 9–10 mm long, grey-green, with rather dense to dense half-black to blackish simple hairs 1–1.5 mm long, rather dense to dense glandular hairs 0.4–0.7(1) mm long and sparse stellate hairs along the margins, apex with some long ciliae. Synflorescence branches with solitary simple hairs, rather dense to dense glandular hairs 0.3–0.5 mm long and stellate hairs. Styles with black papillae. Ligules glabrous.

#### Hieracium firmiramum

Hyl.

E64B68DF-83AB-5396-A886-2838BC665833

##### Native status

Alien. Park introduction, open places among trees.

##### Distribution

Finland: Ab, N; Sweden (Hylander 1943). Only introduced populations are known. The native distribution area has not been traced.

##### Notes

Tyler (2004b) included this species in *Hieraciumpachyodon* Dahlst., which was broadly treated with the inclusion of several other taxa.

##### Diagnosis

Stems 30–70 cm tall. Rosulate leaves oblong-lanceolate to elliptic-ovate, with small to prominent narrow teeth, thin, grass-green, with sparse to dense simple hairs 0.5 mm long above, glabrescent in the middle, on long petioles. Cauline leaf single, often reduced. Phyllaries narrowly triangular, with acute apex, ca. 10 mm long, grey-green, without simple hairs, with abundant glandular hairs 0.7–1 mm long and sparse stellate hairs in the basal part along the margins, apex with some ciliae. Synflorescence branches without simple hairs, with abundant glandular hairs 0.5–0.6 mm long and stellate hairs. Styles with black papillae. Ligules glabrous.

#### Hieracium glandulosissimum

(Dahlst.) Brenner

C8097955-10DF-53F9-9BAA-C42E2FC82A18

##### Native status

Native. Sparse forests, open forest margins.

##### Distribution

Finland: Al; Sweden (Tyler 2006e). Reported also elsewhere in Europe (POWO 2025).

##### Diagnosis

Stems 50–60 cm tall. Rosulate leaves elliptic to lanceolate-elliptic, base broadly cuneate, apex broadly acute, with numerous minute teeth, rather thin, dark-green, glabrous (except for narrow margins) above, on rather long petioles. Cauline leaf similar, reduced in size. Phyllaries narrowly triangular, with acute apex, 10–12 mm long, blackish-green, without simple hairs, with abundant black glandular hairs 0.8–1.3 mm long and few stellate hairs along the margins in the basal part, apex without ciliae. Synflorescence branches without simple hairs, with abundant glandular hairs 0.5–1 mm long and stellate hairs. Styles with black papillae. Ligules glabrous.

#### Hieracium grandidens

Dahlst.

691F19EE-321C-5D08-883B-1276B837475F

##### Native status

Alien. Introduced in old parks, manors and around old summer cottages.

##### Distribution

Finland: N, Sa; also introduced and locally naturalised in Scandinavia (Hylander 1943, Schou 2001, Tyler 2004b, Tyler et al. 2015), European Russia (Sennikov 2000, Sennikov 2006b, Sennikov et al. 2012, Sennikov and Golubeva 2014) and Baltic countries (Sennikov 2003b). The native distribution area is Central Europe, from Germany to Belarus and the Baltic countries (Sennikov 1999b, Sennikov 2003b, Gottschlich 2024), but its western and southern limits are uncertain. Earlier records of the native occurrence in north-western and northern Russia (Schljakov 1989) are erroneous and referable to *H.distractum* or other introduced taxa (Sennikov 2000, Sennikov 2008b).

##### Notes

Schljakov (1989) and his followers (Hackman and Sennikov 1998, Sennikov 1999b, Sennikov 2000, Sennikov 2003b, Sennikov 2006b, Sennikov et al. 2012, Sennikov and Golubeva 2014) accepted *Hieraciumsylvularum* Jord. ex Boreau as the priority name for this species. This decision disagreed with the lectotype of *H.sylvularum*, which was recently established by Gottschlich et al. (2011).

##### Diagnosis

Stems 40–60 cm tall. Rosulate leaves elliptic-ovate to lanceolate-ovate, base sagittate to truncate or broadly cuneate, with large teeth in the basal half, rigid, dark-green, with sparse simple hairs 0.5–0.8(1) mm long above, on rather long petioles. Cauline leaf single, reduced in size. Phyllaries narrowly triangular, with acute apex, 9–10 mm long, grey-green, without simple hairs, with abundant glandular hairs 0.6–1(1.3) mm long and sparse stellate hairs mostly along the margins, apex with a few ciliae. Synflorescence branches without simple hairs, with dense glandular hairs 0.5–0.6 mm long and stellate hairs. Styles with black papillae. Ligules glabrous.

#### Hieracium hjeltii

Norrl.

9CDBBFBF-BAEC-59B1-8122-66C734FCE606

##### Native status

Native. Open places in pine and spruce forests.

##### Distribution

Finland: Ab, N, Ka, St, Ta, Sa, Oa, Tb, Sb, Kb, Om; Sweden (Samuelsson 1954), Baltic countries (Sennikov 2003b), Belarus (Sennikov 1999b), northern European Russia (Samuelsson 1954, Schljakov 1989, Sennikov 2000, Sennikov 2008b) as far as Tver Region in the east (Sennikov 2006b, Sennikov 2006c).

##### Diagnosis

Stems 40–50 cm tall. Rosulate leaves oblong, ovate-oblong to ovate, base broadly cuneate to subrotund, apex broadly triangular, with small repand dentation, rather thin, pale-green, with rare to sparse simple hairs up to 5 mm long above, on rather long petioles. Cauline leaf single, lanceolate-ovate. Phyllaries triangular, with acute apex, 9–10 mm long, pale grey-green, without simple hairs, with dense glandular hairs 0.6–1(1.2) mm long and usually dense stellate hairs throughout and abundant along the margins, apex with abundant coma. Synflorescence branches without simple hairs, with rather dense glandular hairs 0.4–0.6 mm long and stellate hairs. Styles with black papillae. Ligules densely ciliate.

#### Hieracium integratum

(Dahlst. ex Stenstr.) Dahlst.

598BEAF5-C1C6-5800-815D-443DA2B3C2DE

##### Native status

Native. Forest margins.

##### Distribution

Finland: Al; Denmark (Schou 2001), Norway, Sweden (Samuelsson 1954, Tyler 2006e). Reported from other parts of Europe (POWO 2025). Erroneously reported from north-western Russia by Schljakov (1989).

##### Diagnosis

Stems 40–50 cm tall. Rosulate leaves oblong to ovate-oblong, base subrotund, apex broadly triangular to subrotund, with indistinct minute teeth, thin, grass-green, with dense simple hairs 0.5–0.8(1) mm long above, on rather short petioles. Cauline leas single, ovate-lanceolate. Phyllaries triangular, with acute apex, 9–10 mm long, dark blackish-green, without simple hairs, with abundant glandular hairs 0.8–1.2(1.5) mm long and very sparse stellate hairs along the margins, apex with rather numerous long ciliae. Synflorescence branches without simple hairs, with dense glandular hairs 0.3–0.5 mm long and stellate hairs. Styles with black papillae. Ligules glabrous.

#### Hieracium lacerifolium

(Almq. ex Stenstr.) Dahlst.

DDB02134-0363-5EC4-BC96-D148DF79A005

##### Native status

Native. Forest margins.

##### Distribution

Finland: Al; Sweden (Samuelsson 1954, Tyler 2006e).

##### Diagnosis

Stems 40–50 cm tall. Rosulate leaves oblong to ovate-oblong, base broadly cuneate to subrotund, apex broadly triangular to subrotund, with numerous large serrate teeth along the whole margin, thin, pale-green, glabrous above, on long petioles. Cauline leaf single, often rudimentary or absent. Phyllaries narrowly triangular, with acute apex, 10–11 mm long, grey-green, without simple hairs, with rather dense glandular hairs 0.5–0.8(1) mm long and rather dense stellate hairs throughout, apex with abundant short ciliae. Synflorescence branches without simple hairs, with rare to sparse glandular hairs 0.3–0.5 mm long and stellate hairs. Styles with slightly dark (yellowish) papillae. Ligules glabrous.

#### Hieracium lateriflorum

Norrl.

560E1936-DC05-5013-AABB-9CC31FD246A1

##### Native status

Native. Shady sparse spruce forests, forest margins.

##### Distribution

Finland: Al, Ab, N, Ka, St, Ta, Sa, Oa, Tb, Sb, Kb, Om, Obu; northern European Russia (Schljakov 1989, Sennikov 2008b, Sennikov 2009).

##### Diagnosis

Stems 40–50 cm tall. Rosulate leaves oblong, base broadly cuneate to truncate, apex triangular, with sparse small obtuse teeth, thin, dark-green, with rare simple hairs up to 1 cm long above, on rather long petioles. Cauline leaf single, reduced in size. Phyllaries narrowly triangular, with acute apex, 9–10 mm long, dark-green, without simple hairs, with abundant black glandular hairs 0.7–1.5 mm long and sparse stellate hairs along the margins, apex with some short ciliae. Synflorescence branches without simple hairs, with rather dense glandular hairs 0.8–1.2 mm long and stellate hairs. Styles with black papillae. Ligules glabrous.

#### Hieracium lepistoides

(Johanss. ex Dahlst.) Brenner

A25D81FE-36F3-550C-8808-90DB011CB731

##### Native status

Native. Shady sparse spruce and pine forests, forest margins.

##### Distribution

Finland: Ab, N, Ka, St, Ta, Sa, Oa, Tb, Sb, Kb, Om, Obu, Ks; Norway, Sweden (Samuelsson 1954), northern European Russia (Samuelsson 1954, Schljakov 1989, Sennikov 2000, Sennikov 2006c, Sennikov 2008b) up to Ryazan Region in the south (Sennikov 2006b) and the Ural mountains in the east (Sennikov and Golubeva 2014). Reported from numerous other parts of Europe (POWO 2025).

##### Diagnosis

Stems 40–50 cm tall. Rosulate leaves oblong to ovate-oblong, base broadly cuneate or subrotund to truncate, apex broadly triangular, with sparse large narrow teeth, thin, grass-green, with rare to sparse simple hairs 0.5–0.8 mm long above, on long petioles. Cauline leaf single, reduced in size. Phyllaries narrowly triangular, with acute apex, 9–10 mm long, grey-green, without simple hairs, with dense to abundant glandular hairs 0.5–1(1.2) mm long and sparse stellate hairs along the margins, apex with abundant long straight ciliae. Synflorescence branches without simple hairs, with dense glandular hairs 0.3–0.5(0.8) mm long and stellate hairs. Styles with black papillae. Ligules glabrous.

#### Hieracium lyratifolium

Norrl.

1195DA6B-FE74-51B6-AAA4-9945DA1AF5F6

##### Native status

Native. Shrubs, meadows and tundra.

##### Distribution

Finland: Ta, Oa, Sb, Kb, Ok, Obu, Ks, Le; northern Sweden (Tyler 2010b), northern European Russia (Sennikov 2006c).

##### Notes

This species was reported from Russia (Novgorod and Tver Region) as a variant of *Hieraciumchloromaurum* with shorter glandular hairs on phyllaries and synflorescence branches (Sennikov 2006c).

##### Diagnosis

Stems 30–40 cm tall. Rosulate leaves oblong-elliptic to lanceolate-elliptic, base subrotund, broadly cuneate to cuneate, apex obtuse, with indistinct teeth, thin, grass-green, with dense simple hairs ca. 0.5 mm above, on short petioles. Cauline leaf single, similar, reduced in size. Phyllaries narrowly triangular, with acute apex, 9–10 mm long, grey-green, without simple hairs, with very dense glandular hairs 0.5–0.8(1) mm long and sparse stellate hairs throughout, apex without ciliae. Synflorescence branches without simple hairs, with rather dense glandular hairs 0.5–0.6 mm long and stellate hairs. Styles with black papillae. Ligules glabrous.

#### Hieracium meticeps

(Almq. ex Dahlst.) Dahlst.

B14E121C-E6BD-54B9-B182-B71E9CE6D2E6

##### Native status

Native. Forest margins.

##### Distribution

Finland: Al; Sweden.

##### Notes

Tyler (2010a) included this species in *Hieraciumcanipes* (Almq. ex Stenstr.) Dahlst.

##### Diagnosis

Stems 40–50 cm tall. Rosulate leaves oblong or oblong-ovate, base broadly cuneate to subrotund, apex broadly triangular to subrotund, with distant broad teeth, thin, grass-green, with sparse simple hairs up to 0.5 mm long above (glabrescent in the middle), on long petioles. Cauline leaf single, similar to the basal leaves or reduced. Phyllaries linear-triangular, with narrowly acute apex, 9–10 mm long, dark grey-green, with a few simple hairs 0.5–0.8 mm long at the base, rather dense black glandular hairs 0.5–0.8(1) mm long and sparse stellate hairs along the margins, apex without ciliae. Synflorescence branches without simple hairs, with rather dense glandular hairs 0.4–0.6 mm long and stellate hairs. Styles with black papillae. Ligules glabrous.

#### Hieracium neoserratifrons

T.Tyler

ABF1BE8C-2805-5F46-8DC7-E532A6EE6746

Synonym: *Hieraciumserratifrons* Almq. ex Dahlst.

##### Native status

Native. Sparse forests, open forest margins.

##### Distribution

Finland: Al; Sweden (Tyler 2006e).

##### Diagnosis

Stems 50–60 cm tall. Rosulate leaves elliptic to ovate-elliptic, base subrotund, apex broadly acute, with numerous minute teeth, rather thin, dark-green, glabrous (except for narrow margins) above, on mediocre petioles. Cauline leaf similar, reduced in size. Phyllaries narrowly triangular, with broad, rather obtuse apex, 10–12 mm long, blackish-green, without simple hairs, with abundant black glandular hairs 0.7–1.2 mm long and few stellate hairs in the basal part, apex with short ciliae. Synflorescence branches without simple hairs, with dense glandular hairs 0.4–0.7 mm long and stellate hairs. Styles with black papillae. Ligules glabrous.

#### Hieracium orbicans

(Almq. ex Stenstr.) Dahlst.

21DCE88E-12E6-5BA3-ACA0-DFF7C55682BE

##### Native status

Native. Forest margins.

##### Distribution

Finland: Al; Denmark (Schou 2001), Norway, Sweden (Samuelsson 1954, Tyler 2006e), Baltic countries (Sennikov 2003b), Belarus (Sennikov 1999b). Reported also from Hungary (POWO 2025).

##### Diagnosis

Stems 40–50 cm tall. Rosulate leaves elliptic or elliptic-ovate, base broadly cuneate to rotund, apex broadly triangular to subrotund, with distant minute teeth, thin, bright-green, with sparse simple hairs along the margins above, on rather long petioles. Cauline leaf single, elliptic-ovate, reduced in size. Phyllaries narrowly triangular, with acute apex, 10–12 mm long, grey-green, with very rare to rare dark simple hairs ca. 1 mm long, rather dense glandular hairs 1–1.8 mm long and sparse stellate hairs throughout, apex with abundant short ciliae and stellate hairs. Synflorescence branches without or with solitary simple hairs, rather dense glandular hairs 0.5–0.8(1) mm long and stellate hairs. Styles with black papillae. Ligules glabrous.

#### Hieracium panaeolum

Dahlst.

0199A92C-01A8-5F21-9C99-61CCEC9C5FD3

##### Native status

Native. Forest margins.

##### Distribution

Finland: Al; Sweden (Tyler 2006e), Estonia (Sennikov 2003b).

##### Diagnosis

Stems 40–50 cm tall. Rosulate leaves oblong-ovate or ovate, base truncate to sagittate, apex broadly triangular to subrotund, with prominent triangular teeth in the basal part, thin, dark-green, glabrous above, on long petioles. Cauline leaf single, usually completely reduced. Phyllaries narrowly triangular, with acute apex, 9–10 mm long, grey-green, without simple hairs, with rather dense glandular hairs 0.5–0.8(1) mm long and abundant stellate hairs throughout, apex with abundant short ciliae. Synflorescence branches without simple hairs, with dense glandular hairs 0.4–0.5 mm long and stellate hairs. Styles with black papillae. Ligules glabrous.

#### Hieracium parceciliatum

Norrl.

855B7814-E698-5842-995A-9FFC3D8FC5AE

##### Native status

Native. Sparse forests.

##### Distribution

Finland: Tb, Sb, Ks. Endemic to Finland.

##### Diagnosis

Stems 40–50 cm tall. Rosulate leaves oblong, lanceolate-oblong or ovate-oblong, base cuneate to truncate, apex broadly triangular, with numerous small, narrowly triangular, irregular teeth, thin, dark greyish-green, glabrous above, long-ciliate along the margins, on rather long petioles. Cauline leaf single, reduced in size. Phyllaries rather broadly triangular, with acute apex, 10 mm long, dark grey-green, with sparse dark simple hairs 1–1.5 mm long, abundant glandular hairs up to 1.2 mm long (with numerous small hairs in the apical part) and sparse stellate hairs along the margins, apex with abundant flexuose ciliae. Synflorescence branches with solitary simple hairs, very dense glandular hairs 0.5–1 mm long and stellate hairs. Styles with black papillae. Ligules glabrous.

#### Hieracium patale

Norrl.

8AF9A16B-7913-53B0-98CB-6B3E585E0E2B

##### Native status

Native. Forest margins.

##### Distribution

Finland: Al; Sweden (Tyler 2006e).

##### Diagnosis

Stems 30–40 cm tall. Rosulate leaves oblong to broadly oblong, base broadly cuneate to subrotund, apex broadly triangular, with small serrate teeth, thin, bright-green, with sparse to rather dense simple hairs 0.3–0.5 mm long above, on long petioles. Cauline leaf single, often reduced. Phyllaries triangular, with acute apex, 0.8–0.9 mm long, dark grey-green, without simple hairs, with very dense glandular hairs 0.4–0.8(1) mm long and sparse stellate hairs in the basal part along the margins, apex without ciliae. Synflorescence branches without simple hairs, with dense glandular hairs 0.4–0.5 mm long and stellate hairs. Styles with black papillae. Ligules glabrous.

#### Hieracium pellucidum

Laest.

9B93F9DF-952C-52AF-B9AE-9C849657F839

##### Native status

Native. Sparse pine and spruce forests.

##### Distribution

Al, Ab, N, St, Ta, Sa, Oa, Tb, Sb, Kb, Om, Ok, Obu, Ks, Lks, Le; British Isles (Sell and Murrell 2006, Tyler 2014), Germany (Rügen Island) (Gottschlich et al. 1998, Gottschlich 2024), Denmark (Schou 2001), Norway, Sweden (Samuelsson 1954), Baltic countries (Sennikov 2003b), Belarus (Sennikov 1999b), northern and central European Russia (Schljakov 1989, Sennikov 2000, Sennikov 2008b) reaching close to the foothills of the Urals (Sennikov and Golubeva 2014). Reported from Ukraine (POWO 2025).

##### Diagnosis

Stems 40–50 cm tall. Rosulate leaves oblong-ovate or ovate, base rotund to cordate, apex broadly triangular to subrotund, with small teeth mostly at the base, rather thick, pale-green, nearly glabrous above, on long petioles. Cauline leaf single, reduced in size or absent. Phyllaries narrowly triangular, with acute apex, 9–10 mm long, grey-green, with solitary simple hairs up to 1 mm long at the base, abundant glandular hairs 0.7–0.9(1.2) mm long (with numerous small glandular hairs aggregated at the apex), and sparse stellate hairs along the margins, apex with long ciliae. Synflorescence branches without simple hairs, with rare to rather dense glandular hairs 0.3–0.5 mm long and stellate hairs. Styles with black papillae. Ligules glabrous.

#### Hieracium praetenerum

(Almq. ex Dahlst.) Dahlst.

0226C85A-53D0-5452-AEF0-CFEEA5D29974

##### Native status

Native. Sparse pine herb-rich forests.

##### Distribution

Finland: Al, Ab, N, Ka, St, Ta, Sa, Tb, Sb, Ok, Obu, Ks, Le, Li; Norway, Sweden (Samuelsson 1954, Tyler 2006e), Baltic countries (Sennikov 2003b), northern European Russia (Schljakov 1989, Sennikov 2000, Sennikov 2008b).

##### Diagnosis

Stems 30–40 cm tall. Rosulate leaves lanceolate to elliptic or obovate, with small broad teeth, base cuneate, apex cuneate to subrotund, thin, grass-green, with sparse to rather dense simple hairs 0.4–0.5 mm long above, on long petioles. Cauline leaf single, reduced or smaller in size. Phyllaries narrowly triangular, with acute apex, 8–9 mm long, grey-green, with sparse dark simple hairs 0.5–0.8(1) mm long, rather dense glandular hairs 0.3–0.7 mm long and sparse stellate hairs in the basal part, apex with a few long ciliae. Synflorescence branches with solitary simple hairs, sparse glandular hairs 0.3–0.4 mm long and stellate hairs. Styles with black papillae. Ligules glabrous.

#### Hieracium psepharum

(Dahlst.) Sam.

B05F6F4B-2584-5356-9511-3DC8EA3D3D82

##### Native status

Native. Forest margins.

##### Distribution

Finland: Al, Ab, N, Ta; Sweden (Tyler 2006e).

##### Diagnosis

Stems 30–40 cm tall. Rosulate leaves elliptic or lanceolate-elliptic, base cuneate or truncate, apex broadly triangular, with prominent narrow teeth mostly in the basal part, thin, grass-green, glabrous above, on short petioles. Cauline leaf single, usually reduced. Phyllaries linear-triangular, with acute apex, 9–10 mm long, grey-green, without simple hairs, with dense slender glandular hairs 0.5–0.8 mm long and numerous stellate hairs mostly along the margins in the basal part, apex with abundant short ciliae. Synflorescence branches without simple hairs, with rather dense glandular hairs 0.2–0.4 mm long and stellate hairs. Styles with black papillae. Ligules glabrous.

#### Hieracium pseudopellucidum

Brenner

BB71667D-D1F6-551E-8621-8A898D796942

Synonym: *Hieraciumtenuiglandulosum* Norrl.

##### Native status

Native. Sparse spruce and pine forests, forest margins.

##### Distribution

Finland: Ta, Kb, Ok, Ks; northern European Russia (Schljakov 1989, Sennikov 2000, Sennikov 2008b).

##### Diagnosis

Stems 40–50 cm tall. Rosulate leaves oblong-elliptic to lanceolate-elliptic, base subrotund, broadly cuneate to cuneate, apex obtuse, with indistinct teeth, thin, grass-green, with dense simple hairs 0.5–0.8(1) mm long above, on rather long petioles. Cauline leaf 1 (rarely 2), similar, reduced in size. Phyllaries narrowly triangular, with acute apex, 10–11(12) mm long, dark-green, without simple hairs, with abundant slender glandular hairs 0.9–1.5(1.8) mm long and sparse stellate hairs throughout, apex with numerous long ciliae. Synflorescence branches without simple hairs, with dense or abundant glandular hairs 0.5–0.8(1.1) mm long and stellate hairs. Styles with dark papillae. Ligules glabrous.

#### Hieracium savonicum

Norrl.

9805ABFC-2236-5CFC-AE38-EC53AD54DF38

##### Native status

Native. Sparse forests, open forest margins.

##### Distribution

Finland: Oa, Tb, Sb, Ok. Endemic to Finland.

##### Diagnosis

Stems 50–60 cm tall. Rosulate leaves elliptic, ovate-elliptic or lanceolate-elliptic, base broadly cuneate, apex rather obtuse, with small broad teeth, thin, greyish-green, with sparse simple hairs up to 0.5 mm long above (glabrescent in the middle), on long petioles. Cauline leaf similar, reduced in size. Phyllaries narrowly triangular, with acute apex, 10 mm long, greyish-green, without simple hairs, with dense glandular hairs 0.4–0.8 mm long and stellate hairs along the margins and sparsely in the basal part, apex with short ciliae. Synflorescence branches without simple hairs, with dense glandular hairs 0.3–0.6 mm long and stellate hairs. Styles with black papillae. Ligules glabrous.

#### Hieracium subcrassum

(Almq. ex Dahlst.) Brenner

7FEA8200-9B1D-5B8A-9EC6-90D03348C608

##### Native status

Native. Forest margins.

##### Distribution

Finland: Al; Sweden (Tyler 2006e). Reported also from Central Europe (POWO 2025).

##### Diagnosis

Stems 50–60 cm tall. Rosulate leaves narrowly oblong, base truncate to broadly cuneate, apex broadly acute, with large coarse teeth mostly in the basal half, rather thin, dark-green, with dense simple hairs up to 5 mm above, on short petioles. Cauline leaf single, similar, reduced in size. Phyllaries narrowly triangular, with acute apex, 10–11 mm long, blackish-green, with sparse blackish simple hairs 0.5–0.8 mm long (covering the whole length in the central head, at the base in lateral heads), abundant black glandular hairs 0.7–1.2 mm long and sparse stellate hairs along the margins, apex with short ciliae. Synflorescence branches without simple hairs, with dense glandular hairs 0.3–0.5(0.7) mm long and stellate hairs. Styles with black papillae. Ligules glabrous.

#### Hieracium tenebrescens

Norrl.

111D4E96-9785-5DAE-B793-4F82BA423E75

##### Native status

Native. Forest margins.

##### Distribution

Finland: Ab, N, Ta. Endemic to Finland.

##### Diagnosis

Stems 40–60 cm tall. Rosulate leaves lanceolate to lanceolate-ovate, base cuneate, apex subrotund, with sparse prominent serrate teeth in the basal half, thin, grass-green, with sparse simple hairs 0.5–0.7 mm long above, on rather long petioles. Cauline leaf single, lanceolate-ovate. Phyllaries narrowly triangular, with acute apex, 10 mm long, blackish-green, with rather rare black simple hairs 0.8–1 mm long, very dense or abundant black glandular hairs 0.5–0.8 mm long and rare stellate hairs along the margins in the basal part, apex with few ciliae. Synflorescence branches with solitary or very rare simple hairs, very dense or abundant glandular hairs 0.5–0.7 mm long and stellate hairs. Styles with black papillae. Ligules glabrous.

### Sagittata-group

#### Hieracium expallidiforme

(Dahlst. ex Stenstr.) Dahlst.

7754F4FF-2D12-547C-B482-BEBB0B2DC4B0

##### Native status

Native. Forest margins.

##### Distribution

Finland: Al; Norway, Sweden (Tyler 2006e), Estonia (Sennikov 2003b).

##### Diagnosis

Stems 40–50 cm tall. Rosulate leaves oblong-ovate, base truncate to sagittate, apex broadly triangular, subobtuse, with small broad teeth (repand dentation), thin, pale-green, with sparse to rather dense simple hairs up to 0.5 mm above (mostly along the margins, glabrescent in the middle), on long petioles. Cauline leaf single, reduced in size. Phyllaries narrowly triangular, with acute apex, 9–10 mm long, grey-green, with rather dense to dense white simple hairs 1(1.5) mm long, sparse to rather dense glandular hairs 0.2–0.5 mm long and dense stellate hairs along the margins, apex with rather abundant long ciliae. Synflorescence branches with rare simple hairs, rather dense glandular hairs 0.3–0.4 mm long and stellate hairs. Styles with black papillae. Ligules glabrous.

#### Hieracium livescentiforme

Schljakov

9C3F0D69-F350-5917-A4AF-82CFBEB897FB

##### Native status

Native. Tundra.

##### Distribution

Finland: Le; northern European Russia (Schljakov 1989).

##### Diagnosis

Stems 30 cm tall. Rosulate leaves oblong-ovate or lanceolate-ovate, base broadly cuneate, apex triangular or narrowly triangular, with small narrow teeth mostly in the basal part, thin, pale-green, with sparse simple hairs 0.5–0.8 mm long above, on rather long petioles. Cauline leaf single, usually rudimentary. Phyllaries narrowly triangular, with acute apex, 9–10 mm long, grey-green, with sparse to rather dense blackish simple hairs 1 mm long, rather dense black glandular hairs 0.5–0.8 mm long and sparse stellate hairs throughout, apex with some short ciliae. Synflorescence branches with very rare simple hairs, sparse to rather dense glandular hairs 0.4–0.5 mm long and stellate hairs. Styles with dark papillae. Ligules glabrous.

#### Hieracium oistophyllum

Pugsl.

9EBCFC59-7C26-510F-8D3D-FE4480DD8E2A

##### Native status

Native. Pine and spruce forests.

##### Distribution

Finland: Al, Ab, N, Ka, St, Ta, Sa, Tb, Sb; British Isles (Sell and Murrell 2006, Tyler 2014), Germany (Rügen Island) (Gottschlich et al. 1998), Denmark (Schou 2001), Norway, Sweden (Samuelsson 1954, Tyler 2006e), Baltic countries (Sennikov 2003b), Belarus (Sennikov 1999b), northern and central European Russia (Schljakov 1989, Sennikov 2000, Sennikov 2006c, Sennikov 2008b) as far as Kostroma Region to the east (Sennikov 2006b). In Central Europe, the species is known from Germany (Gottschlich 2024) and Poland (POWO 2025).

##### Diagnosis

Stems 40–50 cm tall. Rosulate leaves lanceolate-oblong, oblong or ovate-oblong, base subrotund or truncate, with small spaced teeth, thin, pale-green, with dense to abundant simple hairs 0.8–1 mm above, on rather short petioles. Cauline leaf single, reduced in size. Phyllaries narrowly triangular, with rather acute apex, 9–10 mm long, pale grey-green, with dense to abundant white simple hairs up to 2 mm long throughout the surface, rare to sparse glandular hairs 0.3–0.5 mm long and sparse stellate hairs mostly along the margins, apex with numerous long soft ciliae. Synflorescence branches with dense simple hairs, rather dense to dense glandular hairs 0.3–0.4(0.5) mm long and stellate hairs. Styles with black papillae. Ligules glabrous.

#### Hieracium penduliforme

(Dahlst.) Johanss.

3B94CDA8-166A-576A-AE69-4F74CA6CF44C

Synonym: *Hieraciumconnatum* Norrl.

##### Native status

Native. Pine and spruce forests.

##### Distribution

Finland: N, Ta, Sa, Oa, Tb, Sb, Om, Ks; Sweden (Tyler 2006e), northern European Russia (Schljakov 1989, Sennikov 2008b).

##### Diagnosis

Stems 30–50 cm tall. Rosulate leaves oblong-ovate or lanceolate-ovate, base truncate to sagittate, with small triangular teeth in the basal part, thin, intensely green, with sparse to dense simple hairs 0.5–0.7 mm above, on rather long petioles. Cauline leaf single, often reduced. Phyllaries attenuated, 9–10 mm long, grey-green, with sparse simple hairs 0.7–1.5 mm long, rather dense glandular hairs 0.3–0.5 (0.7) mm long and sparse stellate hairs mostly in the basal half, apex with sparse long ciliae. Synflorescence branches with sparse simple hairs, rather dense glandular hairs 0.3–0.5 mm long and stellate hairs. Styles with black papillae. Ligules glabrous.

#### Hieracium philanthrax

(Stenstr.) Dahlst.

237D6593-88B2-5CED-8A20-45601513A9F5

##### Native status

Native. Spruce forests.

##### Distribution

Finland: Al, Ab, N, Ka, Ta, Sb, Ok; Denmark (Schou 2001), Norway, Sweden (Samuelsson 1954, Tyler 2006e), Baltic countries (Sennikov 2003b), northern Belarus (Sennikov 2002b), northern European Russia (Schljakov 1989, Sennikov 2000).

##### Diagnosis

Stems 40–60 cm tall. Rosulate leaves ovate-oblong, triangular-oblong or lanceolate-oblong, base cuneate to truncate, apex acute, with narrow prominent teeth in the basal part, thin, dark-green, with sparse to dense simple hairs 0.5–0.8 mm long above, on rather short petioles. Cauline leaf single, similar in shape. Phyllaries narrowly triangular, with acute apex, 9–10 mm long, dark grey-green, with rather dense to dense straight black-based to blackish simple hairs 0.8–1(1.5) mm long, sparse to dense glandular hairs 0.2–0.4(0.7) mm long and sparse stellate hairs in the basal part and along the margins, apex with numerous short ciliae. Synflorescence branches with rare simple hairs, dense glandular hairs 0.3–0.4(0.5) mm long and stellate hairs. Styles with black papillae. Ligules glabrous.

### Alpina-group

#### Hieracium alpinum

L.

37A686C7-433D-5A94-92AC-50DC48A87E32

##### Native status

Native. Tundra.

##### Distribution

Finland: Ks, Lkk, Lks, Le, Li; northern and mountainous Norway and Sweden (Samuelsson 1954), northern and mountainous European Russia (Samuelsson 1954, Schljakov 1989) including the Urals in the east (Chepinoga et al. 2024), mountains of Central Europe (POWO 2025).

##### Notes

According to Mráz et al. (2009), only triploid populations of the species are present in Scandinavia and Finland, whereas diploid populations are restricted to the Carpathians. The diploid and triploid populations are taxonomically indistinct.

##### Diagnosis

Stems 15–25 cm tall. Basal leaves pale greyish-green, oblong-lanceolate to spatulate, base narrowly cuneate to attenuate, apex narrowly subrotund to subacute, without or with few indistinct teeth or sometimes with small coarse teeth, on a rather long petiole (1/2–2/3 as long as the lamina), with dense simple hairs 1.5–3.5 mm long above; cauline leaves 1–2, usually strongly reduced. Synflorescence of 1 flowering head. Phyllaries very narrowly triangular to nearly linear, with acute to narrowly attenuated apex, 13–15 mm long, dark-olivaceous, with abundant black-based or dark simple hairs 2–4 mm long, dense glandular hairs 0.2–0.3 mm long and without stellate hairs, apex with some long ciliae. Stems under flowering heads with very dense to abundant simple hairs 2–3.5 mm long, dense stiff glandular hairs 0.3–0.7 mm long and stellate hairs. Styles with yellow papillae. Ligules ciliate.

### Nigrescentia-group

#### Hieracium acuescens

Norrl.

8B6E4A78-7AF0-5A77-97A8-8EDA361A4C01

##### Native status

Native. Tundra.

##### Distribution

Finland: Le. Endemic to Finland.

##### Diagnosis

Stems 20–25 cm tall. Basal leaves greyish-green, lanceolate to spatulate, base narrowly cuneate to attenuate, apex triangular to narrowly subrotund, with sparse small to coarse teeth in the basal half, on a rather short petiole, with rather dense simple hairs 1–1.5 mm long above; cauline leaves 2–3, usually strongly reduced or a lowermost leaf similar to the basal ones. Synflorescence of 1 flowering head. Phyllaries very narrowly triangular to nearly linear, with acute to narrowly attenuated apex, 10–11 mm long, dark-olivaceous, with abundant black-based or dark simple hairs 1.5–2 mm long, rather dense glandular hairs 0.2–0.4 mm long and without stellate hairs, apex with abundant long ciliae. Synflorescence branches with sparse simple hairs ca. 1 mm long, dense slender glandular hairs 0.2–0.4 mm long and sparse stellate hairs (looking dark). Styles with black papillae. Ligules nearly glabrous.

#### Hieracium aquilonium

(Elfstr.) Norrl.

0BE11D29-E3C6-5C7A-86C9-0686E3758FBC

##### Native status

Native. Riversides, tundra.

##### Distribution

Finland: Le; northern Norway and Sweden.

##### Diagnosis

Stems 25–35 cm tall. Basal leaves greyish-green, lanceolate to spatulate, base narrowly cuneate to attenuate, apex triangular to narrowly subrotund, with indistinct or without teeth, on a rather long petiole, with dense simple hairs 1.5–2 mm long above; cauline leaves 2–3, usually strongly reduced. Synflorescence of 1(2) flowering head(s). Phyllaries very narrowly triangular to nearly linear, with acute to narrowly attenuated apex, 11–12 mm long, dark-olivaceous, with abundant black-based simple hairs 2–2.5 mm long, rather dense to dense glandular hairs 0.2–0.5 mm long and without stellate hairs, apex with abundant long ciliae. Synflorescence branches with rather dense simple hairs 2–3.5 mm long, dense stiff glandular hairs 0.5–1 mm long and stellate hairs. Styles with blackish (discoloured) papillae. Ligules sparsely ciliate.

#### Hieracium capnostylum

Dahlst. & Elfstr.

C89A0501-04BC-5D57-8A05-EF4C81E1B1F8

Synonym: *Hieraciumamaurostylum* Dahlst.

##### Native status

Native. Tundra.

##### Distribution

Finland: Le; northern Sweden.

##### Diagnosis

Stems 20–25 cm tall. Basal leaves greyish-green, lanceolate to spatulate or oblong-lanceolate, base narrowly cuneate to attenuate, apex triangular to narrowly subrotund, with sparse coarse teeth in the basal half, on a long petiole, with dense simple hairs 1–1.5 mm long above; cauline leaves 2–3, usually strongly reduced. Synflorescence of 1 flowering head. Phyllaries very narrowly triangular to nearly linear, with acute to narrowly attenuated apex, 11–12 mm long, dark-olivaceous, with abundant black-based or dark simple hairs 2–3 mm long, rather dense to dense glandular hairs 0.2–0.5(0.7) mm long and without stellate hairs, apex with abundant long ciliae. Synflorescence branches with very dense to abundant simple hairs 2–3.5 mm long, dense slender glandular hairs 0.3–0.7 mm long and very sparse stellate hairs (looking blackish). Styles with black papillae. Ligules sparsely ciliate.

#### Hieracium fuliginosum

(Laest.) Andersson

9BD7BF7C-EF3A-5025-85B8-3C38FE7A6D4F

##### Native status

Native. Riversides, tundra.

##### Distribution

Finland: Lkk, Le, Li.; northern Norway and Sweden, northern European Russia (Schljakov 1989).

##### Diagnosis

Stems 20–25 cm tall. Basal leaves greyish-green, lanceolate to rhombic-lanceolate, base narrowly cuneate to attenuate, apex triangular to narrowly subrotund, with a few broad coarse teeth at the middle, on a rather long petiole, with dense simple hairs ca. 2 mm long above; cauline leaves 2–3, lowermost one near the stem base, others strongly reduced. Synflorescence of 1(2) flowering head(s). Phyllaries very narrowly triangular to nearly linear, with acute to narrowly attenuated apex, ca. 13 mm long, dark-olivaceous, with abundant black-based simple hairs 2–3 mm long, dense glandular hairs 0.2–0.5 mm long and without stellate hairs, apex with a few long ciliae. Synflorescence branches with rather dense simple hairs 2–3 mm long, dense stiff glandular hairs 0.2–0.8 mm long and stellate hairs. Styles with blackish (discoloured) papillae. Ligules sparsely ciliate.

#### Hieracium glabriligulatum

Norrl.

FACA7953-5561-5B65-B072-19DDF1A8F1D9

Synonym: *Hieraciumsubaquilonium* (Norrl.) Norrl.

##### Native status

Native. Tundra, riversides.

##### Distribution

Finland: Le, Li; northern Sweden and Norway, northern European Russia (Kravchenko and Sennikov 2009).

##### Notes

The synonymy was established in Kravchenko and Sennikov (2009).

##### Diagnosis

Stems 30–35 cm tall. Basal leaves pale greyish-green, oblong, base cuneate, apex subrotund, with a few broad coarse teeth at the base and minute ones in the basal half, on a long petiole, with dense simple hairs ca. 1.5 mm long above (glabrescent in the middle); cauline leaves 3–4, lowermost one at the stem basal third, reduced in size. Synflorescence of 1(2) flowering head(s). Phyllaries very narrowly triangular to nearly linear, with acute to narrowly attenuated apex, 12–13 mm long, dark-olivaceous, with rather dense to dense black-based simple hairs 2–3 mm long, dense glandular hairs 0.3–0.5 mm long and without stellate hairs, apex with a small coma of long ciliae. Synflorescence branches with rather dense simple hairs 2–3 mm long, dense stiff glandular hairs 0.5–1 mm long and stellate hairs. Styles with blackish (discoloured) papillae. Ligules glabrous.

#### Hieracium lignyotum

Norrl.

7B237090-68FE-57BE-89F6-ED28C8009342

##### Native status

Native. Riversides, shrubs, meadows.

##### Distribution

Finland: Le, Li; northern Norway and Sweden, northern European Russia (Schljakov 1989).

##### Diagnosis

Stems 20–30 cm tall. Basal leaves pale greyish-green, oblanceolate to spatulate, base narrowly cuneate to attenuate, apex subrotund to subacute, with minute to coarse sparse teeth in the basal half, on a rather short to long petiole, with dense simple hairs 0.5–1 mm long above; cauline leaves 1–2, lowermost one at the stem base, reduced in size. Synflorescence of 1–3 flowering heads. Phyllaries very narrowly triangular to nearly linear, with narrowly acute apex, 9–10 mm long, dark-olivaceous, with dense to abundant black-based simple hairs 1.5–2 mm long, dense glandular hairs 0.2–0.5 mm long and with sparse stellate hairs at the base, apex with a small coma of long ciliae. Synflorescence branches with sparse simple hairs 1–1.5 mm long, dense stiff glandular hairs 0.2–0.5 mm long and stellate hairs. Styles with black papillae. Ligules almost glabrous.

#### Hieracium muonioense

Norrl.

F7FD553D-49F0-5232-B2F0-22F106552450

##### Native status

Native. Meadows.

##### Distribution

Finland: Lkk; northern Sweden.

##### Notes

This species is included in *Hieraciumaurigerum* Norrl. in POWO (2025).

##### Diagnosis

Stems 20–30 cm tall. Basal leaves pale greyish-green, oblong-lanceolate, base narrowly cuneate to cuneate, apex narrowly subrotund, with indistinct minute teeth, on a long petiole, with dense simple hairs up to 1.5 mm long above (glabrescent in the middle); cauline leaves 2–3, lowermost one at the stem base, reduced in size. Synflorescence of 1(2) flowering head(s). Phyllaries very narrowly triangular to nearly linear, with rather obtuse apex, 12–13 mm long, dark-olivaceous, with abundant black-based simple hairs 3–4 mm long, dense glandular hairs 0.2–0.3(0.5) mm long and without stellate hairs, apex with a small coma of long ciliae. Synflorescence branches with rather dense simple hairs ca. 2 mm long, dense stiff glandular hairs 0.5–1 mm long and stellate hairs. Styles with black papillae. Ligules unevenly ciliate.

#### Hieracium teligerum

Norrl.

88145057-4941-5DBA-9E38-9EFED7EC6271

##### Native status

Native. Tundra, riversides.

##### Distribution

Finland: Lkk, Le; northern European Russia (Schljakov 1989).

##### Diagnosis

Stems 15–20 cm tall. Basal leaves pale greyish-green, oblong, base cuneate, apex subrotund, teeth minute and indistinct, on a long petiole, with abundant simple hairs 2–3 mm long above; cauline leaves ca. 3, lowermost one at the stem base, similar to the basal ones, others reduced. Synflorescence of 1 flowering head. Phyllaries very narrowly triangular to nearly linear, with acute to narrowly attenuated apex, 11–12 mm long, dark-olivaceous, with dense to abundant blackish simple hairs 3–4 mm long, rather dense to dense glandular hairs 0.3–0.6 mm long and without stellate hairs, apex with a small coma of long ciliae. Synflorescence branches with rather dense simple hairs 2–3 mm long, rather dense stiff glandular hairs 0.5–1.2 mm long and stellate hairs. Styles with black papillae. Ligules unevenly ciliate.

### Atrata-group

#### Hieracium corrasum

Norrl.

F186B817-87EA-50E3-8176-60995E7C96B1

##### Native status

Native. Riversides, tundra.

##### Distribution

Finland: Le; northern Norway and Sweden.

##### Notes

This species is included in *Hieraciumsubellipticum* Elfstr. in POWO (2025).

##### Diagnosis

Stems 20–30 cm tall. Basal leaves plumbeous-green, lanceolate-ovate, oblong-ovate to oblong, base cuneate to truncate, apex triangular, with prominent narrow acute teeth in the middle and the basal half, on a long petiole, with sparse simple hairs ca. 1 mm long along the margins or glabrous above; cauline leaves 2, usually strongly reduced. Synflorescence of 2–3 flowering heads, with rather short slender final branches (central one is not abbreviated). Phyllaries nearly linear, with narrowly acute apex, 11–12 mm long, dark-olivaceous, with rather dense blackish simple hairs 1.5–2 mm long, sparse to rather dense slender glandular hairs 0.2–0.5 mm long and sparse stellate hairs along the margins in the basal part, with narrow glabrous margins, apex with several long ciliae. Synflorescence branches with sparse simple hairs 1–1.5 mm long, dense stiff glandular hairs 0.3–0.5 mm long and stellate hairs. Styles with black papillae. Ligules glabrous.

#### Hieracium corynellum

Norrl.

363B3B7D-E6B4-56B4-972B-F0A5142A1E52

##### Native status

Native. Riversides, tundra.

##### Distribution

Finland: Le. Endemic to Finland.

##### Diagnosis

Stems 20–30 cm tall. Basal leaves dark greyish-green, narrowly lanceolate to spatulate, base narrowly cuneate to attenuated, apex triangular to subrotund, with narrow acute teeth along the margin, on a long petiole, with very dense simple hairs 0.8–1 mm long above; cauline leaf usually 1, much reduced. Synflorescence of 1–3 flowering heads, with short slender final branches (central one is strongly abbreviated). Phyllaries nearly linear, with acute apex, 9–10 mm long, blackish-olivaceous, with very dense half-black or blackish simple hairs ca. 1 mm long, dense slender glandular hairs 0.3–0.6 mm long and without stellate hairs, apex with abundant long ciliae. Synflorescence branches with sparse simple hairs ca. 1 mm long, abundant rather stiff glandular hairs 0.3–0.5 mm long and rather sparse stellate hairs. Styles with blackish (discoloured) papillae. Ligules glabrous.

#### Hieracium cyathodes

Norrl.

E8DAE293-DF79-5031-A46D-CBDA3E31CC15

##### Native status

Native. Riversides, tundra.

##### Distribution

Finland: Le; northern Sweden and Norway.

##### Notes

This species is included in *Hieraciumenantiodon* Omang in POWO (2025).

##### Diagnosis

Stems 20–30 cm tall. Basal leaves plumbeous-green, lanceolate-ovate, oblong-ovate to oblong, base cuneate to truncate or sagittate, apex triangular, with prominent irregularly serrate dentation in the middle and the basal half, on a rather long petiole, glabrous above; cauline leaf 1, at the stem base, reduced in size. Synflorescence of ca. 2 flowering heads, with rather short slender final branches (central one is not abbreviated). Phyllaries narrowly triangular, with acute apex, 12–13 mm long, dark-olivaceous, with sparse blackish simple hairs 1–1.5 mm long, dense stiff glandular hairs 0.5–1.2 mm long and sparse stellate hairs along the margins in the basal part, with very narrow glabrous margins, apex with a few ciliae. Synflorescence branches with solitary to sparse simple hairs 1–1.5 mm long, very dense stiff glandular hairs 0.5–1 mm long and stellate hairs. Styles with black papillae. Ligules glabrous.

#### Hieracium fraudans

Norrl. ex Dahlst.

6528DE40-EF43-5D4C-927F-5A57AC717021

##### Native status

Native. Sparse forest margins, riversides, tundra.

##### Distribution

Finland: Lkk, Lks, Le, Li; northern Sweden and Norway.

##### Diagnosis

Stems 35–45 cm tall. Basal leaves dark greyish-green, oblong to lanceolate-oblong, base broadly cuneate to subrotund, apex broadly triangular to subrotund, with minute dentation along the margin, on a rather long petiole, with sparse simple hairs ca. 1 mm long mostly along the margins above; cauline leaves 1–2, usually much reduced. Synflorescence of 1–6 flowering heads, with rather short slender final branches (central one is not strongly abbreviated). Phyllaries narrowly triangular, with acute apex, 10–11 mm long, dark-olivaceous, with rather dense blackish simple hairs ca. 1 mm long, rather dense stiff glandular hairs 0.3–0.8 mm long and sparse stellate hairs in the basal part, with very narrow glabrous margins, apex with a few ciliae. Synflorescence branches with rare simple hairs 0.5–0.8 mm long, very dense slender glandular hairs 0.5–0.7 mm long and stellate hairs. Styles with black papillae. Ligules glabrous.

#### Hieracium geminatum

Norrl.

C5562F54-9755-5F7C-B813-14E80B9FFAAB

##### Native status

Native. Riversides, tundra.

##### Distribution

Finland: Ok, Lkk, Le; northern European Russia (Schljakov 1989, Sennikov 2008b).

##### Diagnosis

Stems 25–40 cm tall. Basal leaves pale greyish-green, oblong-ovate to lanceolate-ovate, base subrotund, apex broadly triangular to subrotund, with minute dentation along the margin, on a long petiole, with dense simple hairs 0.8–1 mm long (glabrescent in the middle); cauline leaves 1–2, one at the base similar to the basal leaves, another much reduced. Synflorescence of 2–5 flowering heads, with rather short slender final branches (central one is not strongly abbreviated). Phyllaries very narrowly triangular, with narrowly acute apex, 9–10 mm long, dark-olivaceous, with sparse to rather dense blackish simple hairs 0.5–0.8 mm long, rather dense stiff glandular hairs 0.2–0.5 mm long and sparse stellate hairs mostly along margins in the basal part, with very narrow glabrous margins, apex with a few ciliae. Synflorescence branches with solitary to rather rare simple hairs 0.5–0.8 mm long, very dense slender glandular hairs 0.2–0.4 mm long and stellate hairs. Styles with black papillae. Ligules glabrous.

#### Hieracium incurrens

Norrl.

3E81FBBF-7EA0-5510-B64A-9FA5F4041AEB

##### Native status

Native. Spruce forests, forest margins, shrubs, riversides.

##### Distribution

Finland: Ab, N, Ka, St, Ta, Sa, Oa, Tb, Sb, Kb, Om, Ok, Obo, Obu, Ks, Lkk; Sweden (Samuelsson 1954, Tyler 2017), Baltic countries (Sennikov 2003b), northern and central European Russia (Samuelsson 1954, Schljakov 1989, Sennikov 2000, Sennikov 2006b, Sennikov 2006c, Sennikov 2008b), eastwards up to the Urals (Chepinoga et al. 2024).

##### Diagnosis

Stems 50–70 cm tall. Basal leaves dark greyish-green, lanceolate to elliptic-lanceolate, base cuneate, apex broadly triangular to subobtuse, with prominent sparse serrate dentation, thin, with rare to rather dense simple hairs 0.5–1 mm long and sparse stellate hairs above, on rather long petioles. Cauline leaves similar, 2–3. Phyllaries triangular or narrowly triangular, with acute apex, ca. 11 mm long, dark olivaceous, with rather dense blackish simple hairs ca. 1.5 mm long, rather dense blackish glandular hairs 0.5–0.8(1.2) mm long and sparse stellate hairs along the margins, apex without ciliae. Synflorescence branches with rare to sparse simple hairs 1 mm long, rare to sparse glandular hairs 0.2–0.4 mm long and stellate hairs. Styles with dark papillae. Ligules glabrous.

#### Hieracium mallaense

Norrl.

423EB38D-E4F8-5399-8164-AC193D06237A

##### Native status

Native. Tundra, riversides.

##### Distribution

Finland: Le; northern Sweden and Norway.

##### Notes

This species is included in *Hieraciumrubefactum* Johanss. in POWO (2025).

##### Diagnosis

Stems 25–35 cm tall. Basal leaves pale greyish-green, lanceolate to lanceolate-oblong, base narrowly cuneate to cuneate, apex narrowly triangular to triangular, with minute to prominent teeth (may be coarse at the base), on a long petiole, with sparse to dense simple hairs 1–1.5 mm long above (glabrescent in the middle); cauline leaf 1, at the middle of the stem, strongly reduced. Synflorescence of 2–3 flowering heads, with long slender branches. Phyllaries narrowly triangular, with acute apex, ca. 11 mm long, dark-olivaceous, with rather dense to dense blackish simple hairs 1–1.5 mm long, rather dense glandular hairs 0.2–0.7 mm long and rare to sparse stellate hairs in the basal part, without glabrous margins, apex with a few short ciliae. Synflorescence branches with rare to rather rare simple hairs up 1–1.5 mm long, dense stiff glandular hairs 0.5–0.8 mm long and stellate hairs. Styles with black papillae. Ligules glabrous.

#### Hieracium microplacerum

Norrl.

6E559DE2-2F0C-58FC-B642-71BC12D1C147

##### Native status

Native. Meadows, shrubs, riversides.

##### Distribution

Finland: Ks; northern European Russia (Schljakov 1989).

##### Diagnosis

Stems 35–45 cm tall. Basal leaves plumbeous-green, oblong to ovate-oblong, base subrotund to sagittate, apex broadly triangular to subrotund, with small, but prominent teeth at the base and minute dentation in the middle part, on a long petiole, with dense simple hairs ca. 1 mm long above (glabrescent in the middle); cauline leaf 1, similar to the basal ones when at the base and reduced in size when at the middle. Synflorescence of 3–5 flowering heads, with rather short slender branches (central one is abbreviated). Phyllaries narrowly triangular, with acute apex, 10–11 mm long, dark-olivaceous, with very rare to rare blackish simple hairs 0.5–1 mm long mostly at the base, rather dense to dense glandular hairs 0.5–1 mm long and sparse stellate hairs in the basal part, with sparsely pubescent or glabrous margins, apex with a few ciliae. Synflorescence branches with solitary simple hairs up to 1 mm long, dense slender glandular hairs 0.5–0.8 mm long and stellate hairs. Styles with black papillae. Ligules glabrous.

#### Hieracium morulum

(Dahlst.) Dahlst.

6AC09D0D-21C3-5A11-9548-C60739F450C2

Synonym: *Hieraciumlutulentum* Norrl.

##### Native status

Native. Forest margins, shrubs, riversides.

##### Distribution

Finland: Sb, Kb, Ks, Le; Norway, Sweden (Samuelsson 1954), northern European Russia (Schljakov 1989, Sennikov 2008b, Sennikov 2009) up to the Ural Mountains in the east (Chepinoga et al. 2024).

##### Diagnosis

Stems 55–65 cm tall. Basal leaves dark greyish-green, oblong to ovate-oblong or ovate-lanceolate, base broadly cuneate to nearly truncate, apex triangular to broadly triangular, with prominent serrate dentation along the margin, on a long petiole, with dense to very dense simple hairs 0.5–0.8 mm long above; cauline leaf 1 or reduced. Synflorescence of 8–20 flowering heads, with rather short slender final branches (central one is not abbreviated). Phyllaries very narrowly triangular, with narrowly acute apex, ca. 11 mm long, dark-olivaceous, with rather dense blackish simple hairs 1–1.3 mm long, rather dense glandular hairs 0.5–0.8(1) mm long and sparse stellate hairs in the basal part, without glabrous margins, apex with a few ciliae. Synflorescence branches with sparse simple hairs up to 1 mm long, dense slender glandular hairs 0.5–0.8 mm long and stellate hairs. Styles with black papillae. Ligules glabrous.

#### Hieracium semicurvatum

Norrl.

ABEA3B75-8827-5F54-8606-4253D05C891F

##### Native status

Native. Birch forests.

##### Distribution

Finland: Lkk, Le; Norway, Sweden, northern European Russia (Samuelsson 1954).

##### Diagnosis

Stems 35–50 cm tall. Basal leaves greyish-green, rhombic-lanceolate to broadly oblong, base broadly cuneate to truncate, apex broadly triangular to subrotund, with a distinct serrate dentation in the basal and middle parts, on a very long petiole, with dense simple hairs ca. 1 mm long and stellate hairs along the central nerve above; cauline leaf 1, reduced in size. Synflorescence of 2–3 flowering heads, with rather short stiff branches. Phyllaries narrowly triangular, with acute or narrowly attenuated apex, ca. 11 mm long, dark-olivaceous, with rather dense to dense black-based simple hairs 1.5–2 mm long, dense glandular hairs 0.2–0.5 mm long and sparse stellate hairs in the basal part, apex with a small coma of long ciliae. Synflorescence branches with rather dense simple hairs up to 1 mm long, abundant stiff glandular hairs 0.5–0.7 mm long and stellate hairs. Styles with black papillae. Ligules glabrous.

#### Hieracium spilodes

Norrl.

20261CE2-2575-58D9-B511-21AF1195B2E2

##### Native status

Native. Tundra, riversides.

##### Distribution

Finland: Le. Endemic to Finland.

##### Diagnosis

Stems 35–45 cm tall. Basal leaves plumbeous-green, rhombic-oblong to broadly oblong, base broadly cuneate, apex broadly triangular, with a distinct serrate dentation along the margin, on a rather long petiole, with a few simple hairs up to 1 mm long along margins above (nearly glabrous); cauline leaf 1, rudimentary. Synflorescence of 2–3 flowering heads, with long slender branches. Phyllaries very narrowly triangular, with acute apex, ca. 11 mm long, dark-olivaceous, with rather dense to dense blackish simple hairs 1–1.5 mm long, dense glandular hairs 0.5–0.8 mm long and sparse stellate hairs in the base along the margins, apex with a small coma of long ciliae. Synflorescence branches with rather dense simple hairs up to 1 mm long, abundant slender glandular hairs 0.5–0.7 mm long and stellate hairs. Styles with black papillae. Ligules glabrous.

## Identification Keys

### Key to *Hieracium* groups in Finland

**Table:** 

1	Phyllaries dark-olivaceous, with simple hairs 2–4 mm long, which are flexuous or 1–1.5 mm long, which are almost completely dark; leaves with an ochrous tint when dry, with tiny glandular hairs among simple pubescence	2
–	Phyllaries variously coloured, with simple hairs usually 1–1.5 mm long; if hairs on phyllaries are almost completely dark, then leaves without an ochrous tint and tiny glandular hairs	4
2	Flowering heads always single; phyllaries 13–15 mm long, always with abundant simple hairs 2–4 mm long; styles always purely yellow	**Alpina**-group
–	Flowering heads 1 to several; phyllaries 9–13 mm long, often with shorter simple hairs; styles always with black or dark papillae, never purely yellow	3
3	Flowering heads usually single on the end of the stem of its main branches, which are thick and stout; phyllaries (10)11–13 mm long, with dense to abundant simple hairs (1.5)2–4 mm long, usually without or with only very few stellate hairs; leaves mostly lanceolate to oblanceolate, basally narrowly cuneate to attenuate	**Nigrescentia**-group
–	Flowering heads 2 to several, on slender branches (synflorescence resembles that of the Murorum-group); phyllaries 9–11(12–13) mm long, with (rare) sparse to dense simple hairs (0.5)1–1.5(2) mm long, with sparse stellate hairs in the basal part; leaves mostly oblong, lanceolate-ovate or oblong-ovate, basally broadly cuneate to subrotund or truncate	**Atrata**-group
4	Phyllaries with a glaucous tint, usually with setose simple hairs; leaves intensely glaucous, glabrous above, with setose hairs along margins	**Oreadea**-group
–	Phyllaries without a glaucous tint, with non-setose simple hairs; leaves glaucous or not, with thin or rigid, but not setose simple hairs	5
5	Phyllaries and synflorescence branches with numerous simple hairs and scattered very short (0.1–0.3 mm long) glandular hairs; central flowering heads in the synflorescence are often on very short, strongly abbreviated branches	**Constringentia**-group
–	Phyllaries and synflorescence branches variously covered by simple and glandular hairs; central flowering heads in the synflorescence are not abbreviated	6
6	Basal leaves single to few, typically smaller than the lowermost cauline leaves, often withering at anthesis	7
–	Basal leaves in a developed rosette, noticeably larger than the lowermost cauline leaves, usually not withering at anthesis	11
7	Phyllaries with prominently reflexed apices	**Umbellata**-group
–	Phyllaries with tightly or loosely appressed apices	8
8	Middle cauline leaves distinctly panduriform, base amplexicaul; phyllaries and synflorescence branches with abundant glandular hairs; ligules ciliate	**Prenanthoidea**-group
–	Middle cauline leaves indistinctly or not panduriform, base subrotund; synflorescence branches with very rare to sparse glandular hairs; ligules glabrous	9
9	Cauline leaves largely lanceolate-ovate or oblong, base subrotund	**Aestiva**-group
–	Cauline leaves largely lanceolate-ovate or rhombic-lanceolate to linear, base subrotund (upper leaves) or cuneate to attenuated	10
10	Cauline leaves linear or very narrowly lanceolate or ovate-lanceolate, indistinctly broader near the middle or towards the base	**Tridentata**-group
–	Cauline leaves lanceolate, rhombic-lanceolate or ovate-lanceolate, prominently broader in the middle or lower half	**Rigida**-group
11	Leaves mostly oblong, oblong-ovate and triangular-ovate, base broadly cuneate, subrotund, truncate or sagittate, lamina apparently delimited from the petiole	12
–	Leaves mostly lanceolate, oblong-lanceolate or lanceolate-ovate, base variously cuneate, lamina rather gradually narrowing into the petiole	14
12	Phyllaries with apparently longer glandular hairs (0.7–1.5 mm long)	**Murorum**-group
–	Phyllaries with short glandular hairs (usually 0.2–0.5, some up to 0.8 mm long)	13
13	Leaves glabrous to sparsely hairy above; synflorescence branches with very rare to sparse pubescence of simple and glandular hairs	**Bifida**-group
–	Leaves sparsely to densely hairy above; synflorescence branches with dense to very dense pubescence of simple and glandular hairs	**Sagittata**-group
14	Phyllaries without or with very rare (sometimes scattered) simple hairs, mostly at the base of flowering heads or on the outer phyllaries	15
–	Phyllaries with rare, sparse or dense to abundant simple hairs along the whole length, always present in the upper half	17
15	Phyllaries with abundant glandular hairs 0.8–1.5 mm long	**Diaphanoidea**-group
–	Phyllaries with sparse to dense or abundant glandular hairs 0.5–0.8 mm long (only single hairs may reach the length of 1 mm)	16
16	Leaves bright-green or glaucous-green	**Fulvescentia**-group
–	Leaves pale-green, grass-green or dark-green	**Subsimilia**-group
17	Phyllaries with sparse to rather dense simple and glandular hairs, usually covering their central line without the margins	**Caesia**-group
–	Phyllaries broadly covered by very dense or abundant simple and glandular hairs, usually including their margins	**Vulgata**-group

Genus description: Perennial plants with milky juice, variously hairy (simple hairs at various colour, length, thickness and density that are covering stems, leaves and involucres; glandular hairs at various length and density that are covering stems, leaves (Alpina-group and its hybrids, Oreadea-group) and involucres; stellate hairs variously covering stems, leaves and involucres). Rhizomes short. Stems single or few, branching in the upper part, runners absent. Leaves variably green (pale or dark, glaucous or plumbeous, ochrous, sometimes with purple spots above and purplish colouration below), thin or rigid or slightly fleshy, basal (1 to several, developing in 1 year or forming a rosette in two years) and cauline (1 to many, variously distributed along the stem), petiolate or sessile, lamina ovate, oblong, lanceolate or linear, base sagittate, auriculate to rotund, truncate, cuneate or attenuated, apex acute to rounded, margin entire or variously toothed. Synflorescence corymbiform, candelabriform, furcate or single-headed, branches with or without simple and glandular hairs, always with stellate pubescence. Involucre cupuliform or turbiniform; phyllaries multiseriate, tightly or laxly appressed or reflexed, usually covered by variable pubescence of simple, glandular and stellate hairs. Flowers with yellow ligules of various hues (from pale to deep yellow), variably ciliate or glabrous at the apex, sometimes turning tubulate (in Alpina-group and Nigrescentia-group). Styles with sweeping papillae coloured in yellow or black, with intermediate variants. Achenes 3–5 mm long, with 10 unequal (alternating in size) ribs connected in the apical rim, usually brown, but sometimes stramineous; pappus persistent, serrate of shortly protruding cell apices, yellowish.

### Key to the Aestiva-group in Finland

**Table:** 

1	Outer phyllaries oblong to oblong-triangular, broadly covered by short glandular hairs	2
–	Outer phyllaries broadly triangular, covered by some simple and glandular hairs mostly along the central line	3
2	Leaves 2–3 cm wide, with minute indistinct teeth	***Hieraciumpruiniferum***
–	Leaves ca. 1 cm wide, with small serrate dentation	***Hieraciumpseudohypochnoodes***
3	Phyllaries (nearly) without simple hairs	***Hieraciumangustum***
–	Phyllaries with numerous simple hairs	4
4	Phyllaries blackish, nearly without stellate hairs	***Hieraciumcrocatum***
–	Phyllaries dark, with sparse to rather dense stellate hairs	***Hieraciumcondylodes***

Group description: Stems robust, regularly and densely leafy. Basal leaves a few, not forming a rosette, usually withering at anthesis. Cauline leaves numerous, lanceolate-ovate or oblong-ovate, slightly or indistinctly panduriform, basally mostly subrotund, minutely toothed, hairy. Synflorescence usually many-headed, corymbiform. Involucre basally broadly turbinate. Phyllaries mostly oblong, with sparse to dense glandular hairs, some simple and stellate hairs. Synflorescence branches with some glandular and simple hairs. Styles with black papillae. Achenes brown.

### Key to the Rigida-group in Finland

**Table:** 

1	Leaves broadly elliptic-ovate to broadly rhombic-ovate, with purple spots above	***Hieraciumarchaeum***
–	Leaves mostly rhombic-lanceolate to oblong-lanceolate, without apparent purple spots above	2
2	Leaves lanceolate-ovate and oblong-lanceolate, base cuneate to subrotund, sessile	3
–	Leaves lanceolate to rhombic-lanceolate, base narrowly cuneate or petiolate	11
3	Phyllaries broadly triangular	4
–	Phyllaries narrowly triangular	5
4	Phyllaries with rare to rather rare simple hairs 0.5–1 mm long, rare glandular hairs 0.1–0.3 mm long and sparse stellate hairs throughout	***Hieraciumlapponicum***
–	Phyllaries with solitary to rare (or lacking) simple hairs 1–1.3 mm long, rather dense glandular hairs 0.3–0.7(1) mm long and very rare (nearly lacking) stellate hairs	***Hieraciumlaterale***
5	Phyllaries pale grey-green, usually without simple and glandular hairs (sometimes with solitary simple hairs 0.5–0.8 mm long), with sparse to rather dense stellate hairs	***Hieraciumlissolepium***
–	Phyllaries variously covered with apparent simple and glandular hairs	6
6	Phyllaries with sparse to dense simple hairs, visually dominating over glandular hairs	7
–	Phyllaries with solitary to rather rare simple hairs, apparently less numerous than glandular hairs	9
7	Phyllaries nearly without stellate hairs, not grey in appearance	***Hieraciumsubrigidum***
–	Phyllaries greyish of abundant stellate hairs which are densely covering their surface (resembling the phyllaries of *H.vulgatum*)	8
8	Phyllaries narrowly triangular, narrowly covered by simple and glandular hairs along the central line; synflorescence branches with very rare simple hairs and solitary glandular hairs 0.1–0.2 mm long	***Hieraciumgriseellum***
–	Phyllaries triangular, broadly covered by simple and glandular hairs; synflorescence branches with sparse to rather dense simple hairs and rather dense glandular hairs 0.1–0.2 mm long	***Hieraciumgodbyense***
9	Leaves with serrate dentation and very narrow teeth	***Hieraciumsubaureum***
–	Leaves with sparse, narrowly triangular teeth	10
10	Phyllaries triangular, grey-green; leaves grass-green	***Hieraciumobatrescens***
–	Phyllaries broadly triangular, blackish; leaves dark-green	***Hieraciumavae***
11	Phyllaries broadly triangular	12
–	Phyllaries narrowly triangular	13
12	Phyllaries nearly linear-oblong, with solitary simple hairs 0.5–1 mm long and sparse glandular hairs 0.3–0.8 mm long	***Hieraciumrasile***
–	Phyllaries broadly triangular, with rather dense simple hairs 0.8–1(1.3) mm long and rather dense glandular hairs 0.1–0.3 mm long	***Hieraciumtersiflorum***
13	Phyllaries with glandular hairs 1–1.3 mm long, apex with a small coma of short ciliae	14
–	Phyllaries with glandular hairs below 0.8 mm long, apex without a coma	15
14	Phyllaries blackish-green, with glandular hairs throughout, rare simple hairs mostly at the base and scattered stellate hairs	***Hieraciuminternatum***
–	Phyllaries grey-green, with glandular hairs throughout, sparse simple hairs and rather dense stellate hairs (more observable along the margins)	***Hieraciumimpunctatum***
15	Lower cauline leaves basally attenuated, long-petiolate; phyllaries 8–9 mm long	16
–	Lower cauline leaves basally cuneate, shortly petiolate or sessile; phyllaries 9–11 mm long	17
16	Leaves with a narrowly obtuse apex, teeth narrowly triangular to linear; phyllaries narrowly triangular, with solitary simple hairs at the base and sparse glandular hairs 0.3–0.5 mm long	***Hieraciumcornigerum***
–	Leaves with a long-attenuated apex, teeth linear; phyllaries triangular, with rare to rather rare simple hairs 0.7–1 mm long and rather dense glandular hairs 0.3–0.7 mm long	***Hieraciumsavo-karelicum***
17	Cauline leaves 8–10; phyllaries with sparse to rather dense simple hairs ca. 1 mm long, sparse to rather dense glandular hairs 0.3–0.5(0.7) mm long and sparse stellate hairs along the margins and in the basal part, apex with numerous short ciliae; synflorescence branches with some simple and glandular hairs	***Hieraciumorbolense***
–	Cauline leaves 18–25; phyllaries without simple hairs, with sparse to rather dense glandular hairs 0.1–0.5 mm long and sparse stellate hairs in the basal half, apex without ciliae; synflorescence branches without simple and glandular hairs	***Hieraciumfloccimarginatum***

Group description: Stems robust, usually densely and evenly leafy. Basal leaves a few, withering or present at anthesis. Cauline leaves numerous, elliptic-ovate to rhombic-ovate, rhombic-lanceolate to oblong-lanceolate, narrowly oblong, basally subrotund to cuneate or attenuated, sessile or shortly petiolate, variously toothed, usually glabrous, but usually with stellate hairs above. Synflorescence often many-headed, irregularly corymbose or paniculately branched. Involucre basally broadly turbinate. Phyllaries triangular to oblong, with some glandular, simple and stellate hairs. Synflorescence branches usually with few glandular and simple hairs. Styles with yellowish or black papillae. Achenes brown.

### Key to the Tridentata-group in Finland

**Table:** 

1	Leaves purple-spotted	***Hieraciumcruentiferum***
–	Leaves green, not spotted	2
2	Phyllaries with numerous simple hairs 1–1.5 mm long	***Hieraciumlinifolium***
–	Phyllaries without or with some simple hairs up to 1 mm long	3
3	Phyllaries with sparse to rather dense stellate hairs, apex with a small coma of short ciliae	4
–	Phyllaries with very few stellate hairs, apex without ciliae	5
4	Phyllaries with a few simple hairs and glandular hairs 0.3–0.5 mm long	***Hieraciumcrepidioides***
–	Phyllaries with rather rare black-based simple hairs and glandular hairs 0.3–0.8 mm long	***Hieraciumsuccedaneum***
5	Phyllaries with sparse glandular hairs 0.2–0.5 mm long	***Hieraciumdolabratum***
–	Phyllaries with rather dense glandular hairs 0.3–0.8(1–1.5) mm long	***Hieraciumsubumbellatum***

Group description: Stems robust, usually densely and evenly leafy. Basal leaves a few, much reduced in size, usually withering, but sometimes present at anthesis. Cauline leaves numerous, usually linear to narrowly lanceolate, basally narrowly cuneate, sessile, variously toothed, usually glabrous. Synflorescence many-headed, corymbose. Involucre basally turbinate. Phyllaries (narrowly) triangular, with some glandular, simple and stellate hairs along the central line. Synflorescence branches with very few or no glandular and simple hairs. Styles with black (sometimes yellowish) papillae. Achenes brown.

### Key to the Oreadea-group in Finland

**Table:** 

1	Basal leaves very few, withering early; stems with numerous (5–15) cauline leaves	2
–	Basal leaves remain at anthesis, forming a rosette; stems with few (1–3) cauline leaves	6
2	Synflorescence branches with abundant simple and glandular hairs	***Hieraciumsubonosmoides***
–	Synflorescence branches with very rare to sparse, if any, simple and glandular hairs	3
3	Leaves nearly linear	4
–	Leaves broadly lanceolate to ovate-lanceolate	5
4	Phyllaries pale, ca. 9 mm long, with sparse pale simple hairs 1–2 mm long, rather dense pale glandular hairs 0.1–0.2(0.3) mm long and sparse stellate hairs mostly along the margins, apex with a small coma of very short ciliae	***Hieraciumsubobatrescens***
–	Phyllaries dark, ca. 11 mm long, with rare to sparse black-based simple hairs 0.5–0.8 mm long, dense glandular hairs 0.1–0.3 mm long and rather dense stellate hairs in the basal part, apex without ciliae	***Hieraciumcuspidifolium***
5	Phyllaries with rather dense stellate hairs throughout, apex with a small coma of short ciliae; styles yellowish	***Hieraciumrufescens***
–	Phyllaries with sparse stellate hairs along the margins, apex without ciliae; styles black	***Hieraciumnorvegicum***
6	Leaf margins with simple hairs 4–5 mm long	***Hieraciumcrinellum***
–	Leaf margins with simple hairs up to 3 mm long	7
7	Leaves dark; phyllaries dark, their glandular hairs black, 0.2–0.5(0.7–0.9) mm long; styles dark	***Hieraciumargenteum***
–	Leaves pale; phyllaries pale, their glandular hairs pale, 0.1–0.5 mm long; styles yellowish	***Hieraciumsaxifragum***

Group description: Stems slender or robust, sparsely or densely leafy. Basal leaves in a rosette, present or withering at anthesis. Cauline leaves few or numerous, linear or lanceolate, basally narrowly cuneate, sessile, variously toothed, with a glaucous or plumbeous tint, glabrous above, but with setose simple hairs along margins and below. Synflorescence few-headed, irregularly branching or laxly corymbose. Involucre basally cupuliform. Phyllaries (narrowly) triangular, with a glaucous tint, with numerous glandular hairs 0.1–0.4 mm long, usually numerous simple and some stellate hairs. Synflorescence branches with or without glandular and simple hairs. Styles with yellow or black papillae. Achenes brown.

### Key to the Subsimilia-group in Finland

**Table:** 

1	Leaves dark-green, with prominent purple spots on the surface	***Hieraciumdelineatum***
–	Leaves not spotted	2
2	Phyllaries with glandular hairs 0.1–0.4 mm long	***Hieraciumresupinatum***
–	Phyllaries with glandular hairs 0.5–0.7 mm long	3
3	Phyllaries with abundant glandular hairs, covering the whole surface including margins	***Hieraciumimprovisum***
–	Phyllaries with dense to very dense glandular hairs, broadly distributed along the central line, with apparent glabrous margins	4
4	Phyllaries with sparse, scattered stellate hairs	***Hieraciumcaespiticola***
–	Phyllaries with rather dense, apparent stellate hairs	***Hieraciumsubsimile***

Group description: Stems slender, with 2–5 leaves. Basal leaves in a rosette, always present at anthesis, lanceolate or oblong-lanceolate, basally cuneate, petiolate, variously toothed, sometimes with purple spots, variously hairy or subglabrous above. Synflorescence few-headed, irregularly candelabriform. Involucre basally cupuliform. Phyllaries triangular, with short glandular and some stellate hairs, with or without few simple hairs. Synflorescence branches without simple hairs, with some glandular hairs. Styles with black or dark papillae. Achenes brown.

### Key to the Diaphanoidea-group in Finland

**Table:** 

1	Phyllaries broadly triangular, especially the outer ones, with an apparent coma at the apex	2
–	Phyllaries narrowly triangular, with or without ciliae at the apex	3
2	Phyllaries with acute apex; leaves dark-green, with indistinct minute teeth	***Hieraciumsilenii***
–	Phyllaries with broadly obtuse apex; leaves grass-green, with (very) small serrate dentation	***Hieraciumsubarctoum***
3	At least the outer phyllaries with scattered simple hairs	***Hieraciumhyalinellum***
–	Involucres without simple hairs or with a few simple hairs at the very base only	4
4	Phyllaries with sparse to rather dense stellate hairs on the surface and along the margins in the basal half	5
–	Phyllaries with rare to sparse stellate hairs mostly along the margins and very few on the surface in the basal half	6
5	Leaves with sparse irregular dentation; phyllaries with rather numerous ciliae at the apex	***Hieraciumsubpellucidum***
–	Leaves with small, but dense serrate dentation; phyllaries without ciliae at the apex	***Hieraciumacidodontum***
6	Leaves with very sparse minute teeth; phyllaries with abundant glandular hairs 1–2 mm long	***Hieraciumprogrediens***
–	Leaves with rather numerous minute to small serrate teeth; phyllaries with abundant glandular hairs 0.8–1.5 mm long	***Hieraciumdiaphanoides***

Group description: Stems slender to robust, with a few to several leaves. Basal leaves in a rosette, always present at anthesis, lanceolate or oblong-lanceolate, basally cuneate, petiolate, variously toothed, variously hairy (sometimes glabrescent in the centre) above. Synflorescence few-headed, irregularly corymbose. Involucre basally cupuliform. Phyllaries triangular, with abundant long glandular hairs, an admixture of simple hairs and only some stellate hairs. Synflorescence branches with glandular hairs, without simple hairs. Styles with black papillae. Achenes brown.

### Key to the Vulgata-group in Finland

**Table:** 

1	Cauline leaves 3–5	***Hieraciumvulgatum***
–	Cauline leaves 5–10	***Hieraciummegavulgatum***

Group description: Stems slender to robust, with a few to several leaves. Basal leaves in a rosette, always present at anthesis, lanceolate or oblong-lanceolate, basally cuneate, petiolate, with apparent serrate dentation, sparsely to densely hairy above. Synflorescence few-headed, irregularly corymbose. Involucre basally cupuliform. Phyllaries triangular, with abundant simple hairs, small glandular hairs and variable stellate pubescence. Synflorescence branches with some simple and glandular hairs. Styles with black or dark papillae. Achenes brown.

### Key to the Constringentia-group in Finland

**Table:** 

1	Cauline leaves 1–3, lanceolate to rhombic-lanceolate, base cuneate, basal leaves in a strong rosette; phyllaries (narrowly) triangular	2
–	Cauline leaves 3–8, narrowly oblong-lanceolate, base narrowly subrotund, basal leaves few; phyllaries broadly triangular	3
2	Leaves narrowly lanceolate to rhombic-lanceolate, with prominent dentation, densely hairy above	***Hieraciumkuusamoense***
–	Leaves broadly lanceolate, with small acute teeth, sparsely hairy above	***Hieraciumconstringens***
3	Phyllaries and synflorescence branches with simple hairs 1–1.5 mm long	***Hieraciumturbidum***
–	Phyllaries and synflorescence branches with simple hairs 0.5–1 mm long	***Hieraciumbrennerianum***

Group description: Stems slender, with a few (1–4) leaves. Basal leaves always present at anthesis, lanceolate or rhombic-lanceolate, cauline leaves narrowly oblong-lanceolate, lanceolate or rhombic-lanceolate, basally narrowly subrotund or cuneate, sessile or petiolate, with small, but apparent serrate dentation, sparsely to densely hairy above. Synflorescence few-headed, irregularly corymbose, central heads usually on strongly abbreviated branches. Involucre basally cupuliform. Phyllaries triangular, with abundant simple hairs, small glandular hairs and sparse stellate hairs. Synflorescence branches with dense to abundant simple and short glandular hairs. Styles with black papillae. Achenes brown.

### Key to the Caesia-group in Finland

**Table:** 

1	Phyllaries with a prominent white ornamentation along the margins, sharply distinct from the central part	***Hieraciumcaesiomurorum***
–	Phyllaries without ciliae or with some ciliae that do not form a prominent coma	2
2	Phyllaries dark to blackish, long attenuated into a very narrow apex	***Hieraciumplumbeum***
–	Phyllaries not attenuated into a very narrow apex	3
3	Phyllaries with sparse to rather dense glandular hairs up to 0.5–0.7 mm long	4
–	Phyllaries with very rare to sparse glandular hairs up to 0.3(0.5) mm long	5
4	Leaves lanceolate, with rare to rather dense simple hairs above	***Hieraciumcoronarium***
–	Leaves lanceolate to ovate-lanceolate, glabrous above	***Hieraciumlongimanum***
5	Synflorescence branches mostly without or with very rare simple and glandular hairs, which are very short	6
–	Synflorescence branches at least with scattered simple and glandular hairs	7
6	Phyllaries pale greyish-green, with mostly pale simple hairs and dark glandular hairs; leaves pale glaucous-green	***Hieraciumconiops***
–	Phyllaries dark or blackish grey-green, with black-based to half-black simple hairs and black glandular hairs; leaves dark glaucous-green	***Hieraciumumbricola***
7	Phyllaries broadly triangular	8
–	Phyllaries narrowly triangular to triangular	9
8	Cauline leaves 4–7, lanceolate to rhombic-lanceolate; glandular hairs on phyllaries and synflorescence branches rare to sparse	***Hieraciumwainioi***
–	Cauline leaves 1–2, lanceolate-ovate; glandular hairs on phyllaries and synflorescence branches very rare to rare	***Hieraciumravidum***
9	Phyllaries broadly covered by simple and glandular hairs, including apices and margins; simple hairs 1–1.5 mm long	***Hieraciumcaesium***
–	Phyllaries covered by simple and glandular hairs along the central line, with apices and margins mostly devoid of the hairs; simple hairs usually up to 1 mm long	***Hieraciumlaeticolor***

Group description: Stems slender, with a few (1–3) leaves. Basal leaves in a rosette, always present at anthesis, lanceolate or elliptic-lanceolate or lanceolate-ovate, basally cuneate, petiolate, variously toothed, with a slight to prominent glaucous tint, glabrous or sparsely hairy above. Synflorescence few-headed, irregularly corymbose. Involucre basally cupuliform. Phyllaries variously triangular, with dominating simple hairs, very short glandular hairs and more or less numerous stellate hairs. Synflorescence branches with or without some glandular and simple hairs. Styles with black papillae. Achenes brown.

### Key to the Fulvescentia-group in Finland

**Table:** 

1	Phyllaries broadly triangular, 11(12) mm long, pale grey-green, apex with a large coma of abundant ciliae; synflorescence branches with dense glandular hairs	***Hieraciumporrigens***
–	Phyllaries ca. 9 mm long, grey-green, apex with few ciliae; synflorescence branches with solitary to sparse glandular hairs	2
2	Leaves pale-green or bright-green, coarsely dentate; phyllaries pale-green; synflorescence branches with very few glandular hairs	***Hieraciumfulvescens***
–	Leaves grass-green, with small serrate teeth; phyllaries darker, grey-green; synflorescence branches with sparse glandular hairs	***Hieraciumlucens***

Group description: Stems slender, with 2–5 leaves. Basal leaves in a rosette, always present at anthesis, lanceolate or rhombic-lanceolate, basally cuneate, petiolate, variously toothed, sometimes with a glaucous tint, variously hairy or glabrous above. Synflorescence few-headed, irregularly candelabriform. Involucre basally cupuliform. Phyllaries variously triangular, with rather long glandular and stellate hairs, also with few simple hairs. Synflorescence branches with glandular and sometimes simple hairs. Styles with black papillae. Achenes brown.

### Key to the Bifida-group in Finland

**Table:** 

1	Phyllaries with a white ornamentation at the apex and along the margins	2
–	Phyllaries with or without some ciliae and stellate hairs on the apex, never with a white ornamentation	3
2	Leaves triangular-ovate or oblong-ovate, with large coarse teeth; phyllaries with a prominent ornamentation	***Hieraciumtriangulare***
–	Leaves elliptic to lanceolate-elliptic, with minute teeth or nearly entire margins; phyllaries with a thin ornamentation	***Hieraciumniviferum***
3	Leaves narrow, lanceolate-oblong, without simple hairs, but with stellate hairs above	***Hieraciumpendulum***
–	Leaves broadly oblong, oblong-ovate, triangular-ovate or ovate, glabrous or variably hairy, but without stellate hairs above	4
4	Phyllaries narrowly attenuated, with a subulate apex; leaves oblong-ovate, usually coarsely dentate at the base, glabrous	5
–	Phyllaries triangular, with a narrowly to broadly acute, but not subulate apex	7
5	Phyllaries with dense stellate hairs; synflorescence branches with rare simple hairs and very rare glandular hairs	***Hieraciumstenolepis***
–	Phyllaries with sparse stellate hairs; synflorescence branches with sparse simple and glandular hairs	6
6	Phyllaries with glandular hairs 0.2–0.4 mm long, which are blackish and easily observable	***Hieraciumacidotum***
–	Phyllaries with glandular hairs 0.1–0.3 mm long, which are discoloured and little recognisable among simple hairs	***Hieraciumcrispulum***
7	Phyllaries broadly triangular, with dense stellate hairs; basal leaves usually ovate, with prominent dentation	8
–	Phyllaries narrowly triangular	9
8	Phyllaries with very rare glandular hairs 0.1–0.3 mm long; synflorescence branches with solitary glandular hairs 0.2–0.3 mm long	***Hieraciumcaesiiflorum***
–	Phyllaries with sparse to rather dense glandular hairs 0.3–0.5(0.7) mm long; synflorescence branches with rare to sparse glandular hairs 0.3–0.5 mm long	***Hieraciumchlorellum***
9	Basal leaves oblong-triangular or lanceolate-triangular, elongated, innermost basal and cauline leaves nearly linear, with prominent or coarse acute teeth	10
–	Basal leaves oblong, ovate-oblong or ovate, with small teeth	11
10	Phyllaries with rare to sparse simple hairs and sparse to rather dense glandular hairs 0.3–0.6(0.8) mm long	***Hieraciumprolixum***
–	Phyllaries with sparse to rather dense simple hairs and rare glandular hairs 0.2–0.4(0.5) mm long	***Hieraciumcaesitium***
11	Basal leaves with small acute teeth; phyllaries with sparse to rather dense glandular hairs, apex with conspicuous long ciliae; synflorescence branches with rare simple hairs and rare to sparse glandular hairs 0.2–0.4 mm long	***Hieraciummultifrons***
–	Basal leaves with repand dentation; phyllaries with rather dense to dense black glandular hairs, apex with a few long ciliae; synflorescence branches with solitary simple hairs and usually a few glandular hairs 0.2–0.3 mm long	***Hieraciumsubholophyllum***

Group description: Stems slender, with a single, usually strongly reduced leaf. Basal leaves in a rosette, always present at anthesis, oblong, elliptic, elliptic-ovate or ovate, basally broadly cuneate, rotund, truncate or sagittate, petiolate, variously toothed, often with a glaucous tint, glabrous or sparsely hairy above. Synflorescence few-headed, irregularly candelabriform. Involucre basally cupuliform. Phyllaries variously triangular, with dominating simple hairs, very short glandular hairs and more or less numerous stellate hairs. Synflorescence branches with or without some glandular and simple hairs. Styles with black papillae. Achenes brown.

### Key to the Murorum-group in Finland

**Table:** 

1	Phyllaries with a prominent white ornamentation at the apex and along the margins; ligules densely ciliate at the apex	***Hieraciumhjeltii***
–	Phyllaries with or without some ciliae and stellate hairs on the apex, never with a prominent ornamentation	2
2	Phyllaries always without simple hairs, even at the base	3
–	Phyllaries always with simple hairs, at least a few at the base	19
3	Leaves glabrous above (sometimes with few simple hairs along narrow margins)	4
–	Leaves with sparse to dense simple hairs above (sometimes glabrescent in the centre, but pubescent broadly otherwise)	8
4	Phyllaries blackish, nearly lacking stellate hairs, which may be present very narrowly along margins	5
–	Phyllaries greyish, with sparse to abundant stellate hairs on the surface and more prominently along margins	6
5	Leaves oblong to oblong-ovate, with mostly subrotund base	***Hieraciumneoserratifrons***
–	Leaves lanceolate-oblong, with mostly cuneate base	***Hieraciumglandulosissimum***
6	Leaves with large coarse teeth	***Hieraciumlacerifolium***
–	Leaves with apparent, but small dentation	7
7	Phyllaries with stiff glandular hairs; leaves oblong-ovate or ovate, basally often sagittate with a couple of prominent teeth facing downwards	***Hieraciumpanaeolum***
–	Phyllaries with slender glandular hairs; leaves narrowly oblong, basally often truncate with incised dentation	***Hieraciumpsepharum***
8	Leaves lanceolate-oblong to oblong, with indistinct dentation, except for a few small teeth at the base	9
–	Leaves with apparent or even prominent acute or obtuse teeth	12
9	Phyllaries apically with numerous long ciliae; leaves with dense simple hairs 0.5–0.8(1) mm long above	10
–	Phyllaries apically without apparent ciliae; leaves with sparse simple hairs mostly 0.3–0.5 mm above	11
10	Phyllaries with strong glandular hairs 0.8–1.2(1.5) mm long	***Hieraciumintegratum***
–	Phyllaries with slender glandular hairs 0.9–1.5(1.8) mm long	***Hieraciumpseudopellucidum***
11	Phyllaries with abundant glandular hairs 0.8–1.5(1.8) mm long and stellate hairs only along the margins	***Hieraciumchloromaurum***
–	Phyllaries with very dense glandular hairs 0.5–0.8(1) mm long and sparse stellate hairs on the surface	***Hieraciumlyratifolium***
12	Leaves with simple hairs up to 0.5 mm long above	13
–	Leaves with simple hairs 0.8–1 mm and longer above	17
13	Phyllaries with rather dense stellate hairs on the surface, apically with abundant short ciliae; leaves broadly glabrescent, with simple hairs mostly along the margins above	***Hieraciumdiminuens***
–	Phyllaries with stellate hairs mostly along the margins, with or without rare stellate hairs on the surface, apically with inconspicuous short ciliae	14
14	Phyllaries with small, but conspicuous coma at the apex, with sparse stellate hairs on the surface	***Hieraciumsavonicum***
–	Phyllaries with some ciliae, but not an apparent coma at the apex, with stellate hairs mostly along the margins	15
15	Leaves with simple hairs mostly along the margins above, largely glabrous in the centre	***Hieraciumfirmiramum***
–	Leaves evenly covered by simple hairs above	16
16	Phyllaries with very dense to abundant glandular hairs 0.6–1(1.5) mm long	***Hieraciumdistractum***
–	Phyllaries with very dense glandular hairs 0.4–0.8(1) mm long	***Hieraciumpatale***
17	Phyllaries with a conspicuous coma of long straight ciliae; styles usually yellow; leaves grass-green	***Hieraciumlepistoides***
–	Phyllaries without ciliae at the apex; styles black; leaves dark-green	18
18	Leaves dark-green, glossy; phyllaries with dark, but not blackish glandular pubescence	***Hieraciumgrandidens***
–	Leaves very dark-green, dull; phyllaries with blackish glandular pubescence	***Hieraciumlateriflorum***
19	Phyllaries with very rare simple hairs, usually recognisable in the central capitula and often absent in the lateral ones	20
–	Phyllaries with sparse to rather dense simple hairs, covering all capitula	23
20	Leaves narrowly oblong, with large prominent teeth	***Hieraciumsubcrassum***
–	Leaves with very small teeth	21
21	Leaves mostly ovate, basally with a couple of apparent teeth facing downwards, with small or obscure dentation otherwise, glabrescent above; phyllaries with abundant small glandular hairs crowded at the apex	***Hieraciumpellucidum***
–	Leaves oblong to oblong-ovate; phyllaries without abundant small glandular hairs crowded at the apex	22
22	Leaves broadly oblong to subrotund, with very small teeth; phyllaries apically with an apparent coma of long ciliae, marginally with numerous stellate hairs	***Hieraciumorbicans***
–	Leaves lanceolate-oblong, ovate-oblong to broadly oblong, with small, but prominent narrow teeth; phyllaries apically with a few ciliae, marginally with very few stellate hairs	***Hieraciummeticeps***
23	Phyllaries with abundant glandular hairs broadly covering the surface in the upper part	24
–	Phyllaries with dense glandular hairs narrowly covering the surface in the upper part	27
24	Leaves narrowly oblong or slightly obovate, base cuneate; phyllaries with a narrow apex narrowly covered by small glandular hairs	25
–	Leaves oblong-ovate to oblong, base broadly cuneate to subrotund; phyllaries with a broadly triangular apex broadly covered by abundant small glandular hairs	26
25	Leaves with minute repand dentation, with rather dense simple hairs 0.5–1 mm long above	***Hieraciumdispansiforme***
–	Leaves with sparse prominent serrate teeth in the basal half, with sparse simple hairs 0.5–0.7 mm long above	***Hieraciumtenebrescens***
26	Leaves with numerous coarse simple hairs up to 2 mm long above	***Hieraciumciliatiflorum***
–	Leaves mostly glabrous above	***Hieraciumparceciliatum***
27	Leaves narrowly oblong, oblong to obovate, with dense serrate dentation	28
–	Leaves lanceolate-oblong to oblong, with sparse repand dentation	29
28	Phyllaries small, 8–9 mm long; leaves typically 3–4 cm wide; phyllaries with sparse simple hairs 0.5–0.8(1) mm long	***Hieraciumpraetenerum***
–	Phyllaries of average size, 9–10 mm long; leaves typically 5–6 cm wide; phyllaries with rather dense to dense simple hairs 1–1.5 mm long	***Hieraciumfenno-orbicans***
29	Phyllaries with glandular hairs 0.8–1.5 mm long and sparse stellate hairs along the margins	***Hieraciumaltipes***
–	Phyllaries with glandular hairs 0.5–1 mm long and rather dense stellate hairs on the surface	***Hieraciumcanipes***

Group description: Stems slender, with a single, sometimes two leaves, which can be reduced in size. Basal leaves in a rosette, always present at anthesis, oblong, elliptic, elliptic-ovate or ovate, basally broadly cuneate, rotund, truncate or sagittate, petiolate, variously toothed, glabrous or variously hairy above. Synflorescence few-headed, irregularly candelabriform. Involucre basally cupuliform. Phyllaries variously triangular, with numerous long glandular hairs, also possibly with simple and stellate hairs. Synflorescence branches with or without some glandular and simple hairs. Styles with black or yellow papillae. Achenes brown.

### Key to the Sagittata-group in Finland

**Table:** 

1	Phyllaries with simple hairs 1.5–2 times longer than glandular hairs; leaves narrowly oblong-ovate or lanceolate-ovate	***Hieraciumlivescentiforme***
–	Phyllaries with simple hairs 2–4 times longer than glandular hairs; leaves oblong or oblong-ovate	2
2	Phyllaries with black-based or half-black, usually straight simple hairs 0.8–1(1.5) mm long and glandular hairs 0.3–0.5 (0.7) mm long	3
–	Phyllaries with nearly white and flexuous simple hairs 1–1.5(2) mm long and glandular hairs 0.2–0.5 mm long	4
3	Phyllaries with sparse simple hairs and rather dense glandular hairs, apex with sparse long ciliae	***Hieraciumpenduliforme***
–	Phyllaries with rather dense to dense simple hairs and sparse to dense glandular hairs, apex with numerous short ciliae	***Hieraciumphilanthrax***
4	Leaves with small acute teeth, with dense to abundant simple hairs 0.8–1 mm above	***Hieraciumoistophyllum***
–	Leaves with repand dentation, with sparse to rather dense simple hairs up to 0.5 mm above (mostly along the margins, glabrescent in the middle)	***Hieraciumexpallidiforme***

Group description: Stems slender, with a single, sometimes reduced leaf. Basal leaves in a rosette, always present at anthesis, mostly oblong, oblong-ovate or lanceolate-ovate, basally broadly cuneate, rotund, truncate or sagittate, petiolate, variously toothed, sparsely to densely hairy above. Synflorescence few-headed, irregularly candelabriform. Involucre basally cupuliform. Phyllaries variously triangular, with numerous simple hairs, short glandular hairs and some stellate hairs. Synflorescence branches with some glandular and simple hairs. Styles with black papillae. Achenes brown.

### Key to the Nigrescentia-group in Finland

**Table:** 

1	Phyllaries up to 12 mm long	2
–	Phyllaries 12–14 mm long	6
2	Leaves without or with indistinct teeth	3
–	Leaves with small but apparent dentation	4
3	Phyllaries with simple hairs 2–2.5 mm long	***Hieraciumaquilonium***
–	Phyllaries with simple hairs 3–4 mm long	***Hieraciumteligerum***
4	Synflorescence usually branched; phyllaries 9–10 mm long	***Hieraciumlignyotum***
–	Flowering heads usually single on the stem top, rarely 2	5
5	Phyllaries 10–11 mm long, with simple hairs 1.5–2 mm long; synflorescence branches with sparse simple hairs ca. 1 mm long and glandular hairs 0.2–0.4 mm long	***Hieraciumacuescens***
–	Phyllaries 11–12 mm long, with simple hairs 2–3 mm long; synflorescence branches with very dense to abundant simple hairs 2–3.5 mm long and glandular hairs 0.3–0.7 mm long	***Hieraciumcapnostylum***
6	Phyllaries with obtuse apex	***Hieraciummuonioense***
–	Phyllaries with narrowly acute apex	7
7	Phyllaries with abundant simple hairs	***Hieraciumfuliginosum***
–	Phyllaries with rather dense to dense simple hairs	***Hieraciumglabriligulatum***

Group description: Stems robust, simple or furcately branched, with 1–3 strongly reduced leaves. Basal leaves in a rosette, always present at anthesis, mostly lanceolate to oblanceolate, basally narrowly cuneate to attenuate, petiolate, entire or coarsely dentate, with dense long hairs above. Heads single or a few in the end of branches, large. Involucre broadly cupuliform. Phyllaries narrowly triangular to linear, with abundant long flexuous simple hairs, very short glandular hairs and nearly without stellate hairs. Stems under flowering heads with abundant simple and glandular hairs. Styles with black or dark papillae. Achenes brown.

### Key to the Atrata-group in Finland

**Table:** 

1	Leaf blades large, 8–15 cm long; synflorescences large, usually with 8–20 flowering heads	2
–	Leaf blades small, up to 5 mm long; synflorescences smaller, usually with 2–5 flowering heads	3
2	Basal leaves oblong to ovate-oblong or ovate-lanceolate, base broadly cuneate to nearly truncate, with dense to very dense simple hairs 0.5–0.8 mm long above; cauline leaf 1 or reduced	***Hieraciummorulum***
–	Basal leaves lanceolate to elliptic-lanceolate, base cuneate, with rare to rather dense simple hairs 0.5–1 mm long and sparse stellate hairs above; cauline leaves 2–3	***Hieraciumincurrens***
3	Basal leaves minute (up to 3 cm long), narrowly lanceolate to spatulate, base narrowly cuneate to attenuate, regularly with narrow acute teeth along the margin	***Hieraciumcorynellum***
–	Basal leaves 3–5 cm long, broadly lanceolate, rhombic or lanceolate-ovate, base cuneate to truncate (rarely narrowly cuneate), variously toothed to subentire	4
4	Leaves at least partly lanceolate to broadly lanceolate or rhombic, usually with prominent small teeth	5
–	Leaves mostly oblong to oblong-ovate, usually with minute or indistinct dentation	7
5	Phyllaries 9–10 mm long, with simple hairs 0.5–0.8 mm long, rather dense stiff glandular hairs 0.2–0.5 mm long	***Hieraciumgeminatum***
–	Phyllaries 10–11 mm long, with simple hairs up to 1 mm long and glandular hairs up to 0.8–1 mm long	6
6	Phyllaries with rather dense simple hairs and glandular hairs 0.3–0.8 mm long	***Hieraciumfraudans***
–	Phyllaries with very rare to rare simple hairs mostly at the base and glandular hairs 0.5–1 mm long	***Hieraciummicroplacerum***
7	Basal leaves larger, blade usually 5–6 cm long, with sparse to dense simple hairs above	8
–	Basal leaves smaller, blade usually 3–4 cm long, glabrous or nearly so above	9
8	Phyllaries with simple hairs 1–1.5 mm long and glandular hairs 0.2–0.7 mm long	***Hieraciummallaense***
–	Phyllaries with simple hairs 1.5–2 mm long and glandular hairs 0.2–0.5 mm long	***Hieraciumsemicurvatum***
9	Basal leaves with small triangular teeth	***Hieraciumspilodes***
–	Basal leaves with long narrow teeth	10
10	Phyllaries nearly linear, with narrowly acute apex, 11–12 mm long, with rather dense simple hairs 1.5–2 mm long, sparse to rather dense slender glandular hairs 0.2–0.5 mm long; synflorescence branches with simple hairs and glandular hairs 0.3–0.5 mm long	***Hieraciumcorrasum***
–	Phyllaries narrowly triangular, with acute apex, 12–13 mm long, with sparse simple hairs 1–1.5 mm long, dense stiff glandular hairs 0.5–1.2 mm long; synflorescence branches with simple hairs and glandular hairs 0.5–1 mm long	***Hieraciumcyathodes***

Group description: Stems slender, with 1–2(3) leaves. Basal leaves in a rosette, always present at anthesis, mostly oblong, lanceolate-ovate or oblong-ovate, basally broadly cuneate to subrotund or truncate, petiolate, entire or coarsely dentate, with sparse to dense hairs above. Synflorescence irregularly branched or laxly corymbiform. Involucre broadly cupuliform. Phyllaries triangular, with numerous dark simple hairs, short glandular hairs and sparse stellate hairs. Synflorescence branches with simple and glandular hairs. Styles with black or dark papillae. Achenes brown.

## Analysis

The occurrence data for the *Hieracium* species in Finland (the collection-based presence-absence data for each species in each biogeographical province, combined with the estimated abundance data) demonstrate a slightly higher taxonomic diversity in the south, whereas the species abundance is also much greater in southern Finland than in northern Ostrobothnia and southern Lapland (Fig. 3). However, the species numbers in Lapponia enontekiensis and Kuusamo, which are two well-explored regions in the Finnish north, are high again, thus showing the potential for a great taxonomic diversity in the Nigrescentia-group and the Atrata-group. A similar pattern of the high taxonomic diversity in the north was revealed in Russian Lapland (Schljakov 1989).

**Figure 4.**
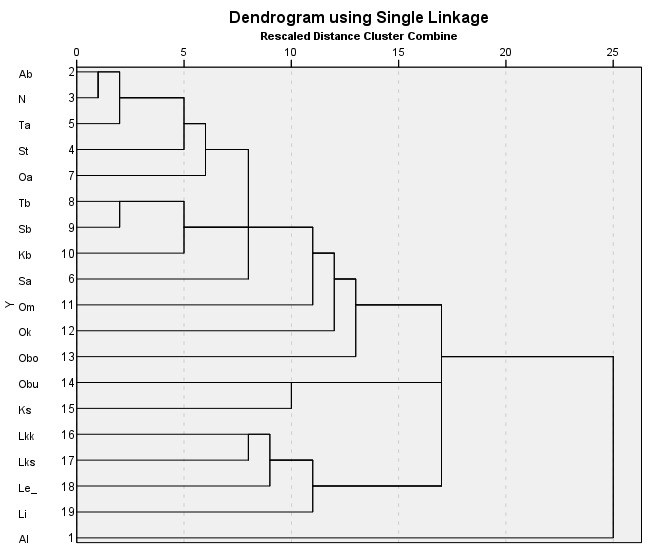
A dendrogram of the hierarchical cluster analysis showing spatial relationships within the territory-level occurrence dataset of native *Hieracium* species in Finland. Province codes and their linear sequence as in Fig. 1b.

The occurrence data have been analysed in a hierarchical cluster analysis in order to uncover main patterns of the species distributions in the country. Despite the provisional character of the present inventory, the resulting dendrogram (Fig. 4) demonstrates a strong phytogeographical signal.

Surprisingly, the most dissimilar *Hieracium* species set has been found in the Åland Islands, by far the smallest biogeographic province of Finland in land size. While a common pattern in the Finnish vascular plants is a gradual extension of termophilous and oceanic elements along the southern coast, including the neighbouring islands (Lahti et al. 1988), the *Hieracium* species set of the Åland Islands has a low level of similarity with any other southern Finnish territory not only due to a high presence of largely western ("southern Swedish") taxa, but also because of a prominent shortage in typically eastern ("southern Finnish") taxa. Common southern (western and eastern) *Hieracium* taxa are also present in the Åland Islands, but far from sufficiently to outnumber the specific ones.

A large group of southern and central Finnish regions with a high level of *Hieracium* species richness and abundance occupies a central position in the dendrogram. Two distinct subgroups are observable in this group and both can be reasonably interpreted taxonomically and phytogeographically. The first subgroup is characterised by the abundance of the Oreadea-group and occurs mostly along the south-western area with a higher temperature and oceanity (Ahti et al. 1968). The second subgroup refers to central and south-eastern Finland, with the addition of the continental members of the Prenanthoidea-group and Aestiva-group. Both patterns were observed in the spatial distribution of the vascular plants in general (Lahti et al. 1988).

The third prominent group covers the northern regions of Finland, i.e. Lapland. This area is characterised by the extension of the Scandinavian mountains (Corner 2005) and the extensive distribution of tundra landscapes and plant communities (Wielgolaski 1975). Characteristic of the northern group are *Hieraciumalpinum* and its hybrid derivatives, i.e. the Nigrescentia-group and the Atrata-group. Nevertheless, other taxonomic groups, which are common between the north and south, are often represented by different species in the northern and southern areas, with very few being common to both areas. The Kuusamo Region and its adjacent territories represent a transition between the northern (tundra) and southern (forest) zones (Hämet-Ahti 1979, Lahti et al. 1988), also harbouring a mixture of *Hieracium* taxa that reach this area from both north and south.

**Figure 5.**
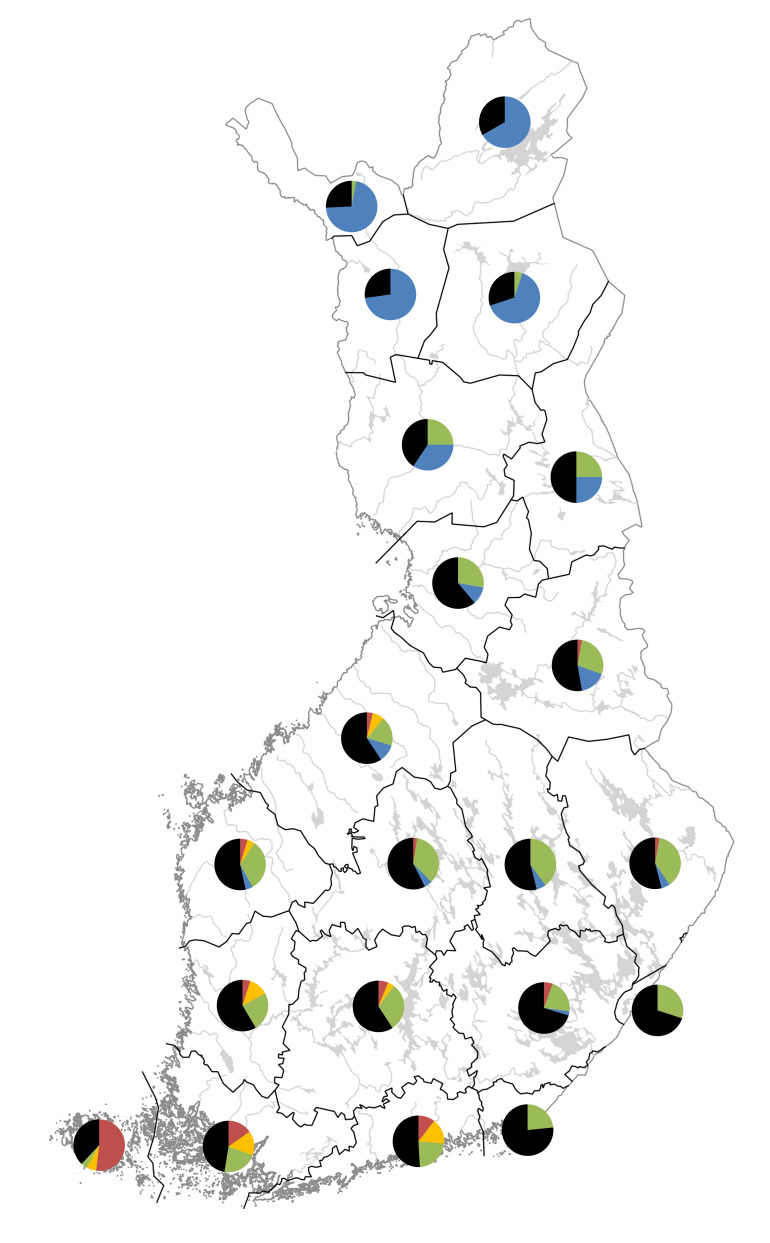
Major geographical elements in the native *Hieracium* species of Finland. For province names and codes, see Fig. 1b. Colour codes: Red - western; Yellow - oceanic; Green - eastern; Blue - northern; Black - common.

Five geographical elements in the native *Hieracium* species of Finland, following Lahti et al. (1988), are visualised in Fig. 5. The western element (29 species) belongs to the western (Scandinavian) sector of Fennoscandia; these species have the main distribution in Norway and Sweden, but extend to Finland mostly in the Åland Islands. The western species are also present, to a minor extent, in the Finnish mainland, but are largely restricted to the extreme south-west (Samuelsson 1954). The oceanic element (10 species) may be considered a variant of the western one, but its species are prominently restricted to the south-western coastal areas, thus showing oceanic climatic preferences. The eastern element (33 species) is characterised by the centre of its distribution placed in southern and central Finland; these species are partly endemic to Finland, but largely extend their distributions to the neighbouring Russia (Schljakov 1989, Sennikov 2000) or, sometimes, Norrbotten in northern Sweden (Tyler 2010b). The northern element (32 species) largely belongs to Lapland with only minor extensions southwards. The common element (31 species) is composed of the southern species which evenly cover both sectors, Scandinavian and Finnish, with a possible presence in the Baltic countries and also elsewhere in Europe and even in Asia.

**Figure 6.**
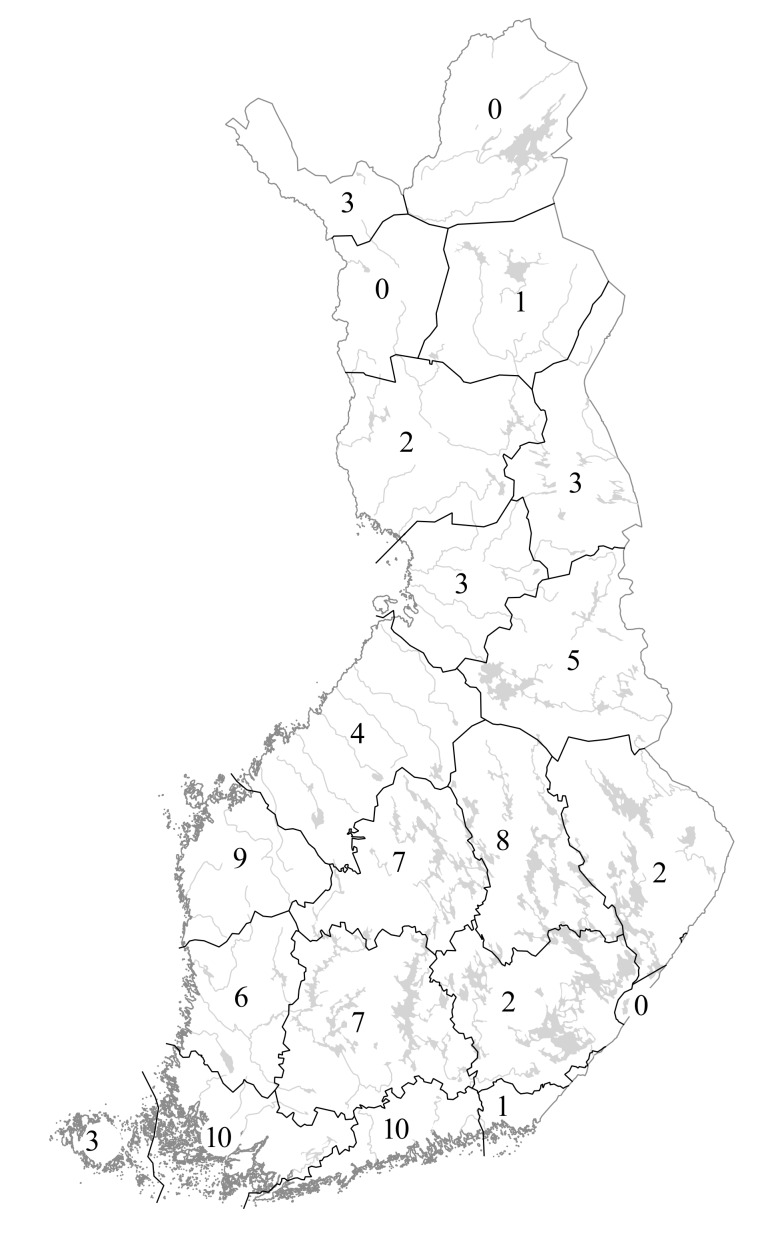
Province-level statistical distribution of *Hieracium* endemics in Finland. For province names and codes, see Fig. 1b.

Altogether, there are 25 species that are deemed endemic to Finland (Fig. 6). This figure is highly provisional because some taxa can still be found identical to Swedish species or occurring also in the Russian west or in the Swedish and Norwegian north. On the other hand, this number can also be increased with a deeper revision of the local species diversity, especially in taxonomically unresolved groups and the poorly-explored north. Nevertheless, the current data show that the number of endemic species is higher in the southern and central provinces, thus indicating a higher proportion of the southern and continental element among the endemic taxa. The majority of these endemic species (16 or ca. 50%) belong to the eastern geographic element, stressing the importance of Finland as a regional taxonomic diversity centre of the genus.

## Discussion

This work summarises the current knowledge of the taxonomy and distribution of *Hieracium* in Finland and lays the foundation for a new taxonomic revision of the genus in this country. The present checklist identifies currently resolved taxa, i.e. those which can be provided with diagnostic characters and classified and for which some reliable distributional data exist. Several local taxa, mostly known from the type material only, have been described from Finland by J.P. Norrlin in his later years or by M. Brenner (Sennikov 2002a) and have never been subjected to taxonomic evaluation and included into any synoptic revision or compilation. Such taxa currently remain unresolved and will be addressed in the near future, with their new catalogue (in preparation) serving for a gap analysis, to provide the first guidance towards a new revision.

Species are classified into unranked groups which are smaller than informal groups previously accepted in Finland (Hackman and Sennikov 1998) and sections or subsections of East European authors (Schljakov 1989), but broader than aggregates currently elaborated for Sweden (Tyler 2006e, Tyler 2017). At present, these groups may be partly heterogeneous (especially Murorum group and Rigida group), but their further subdivision depends on more refined taxonomic knowledge which is not yet available in Finland. Their current use is, therefore, considered a practical, rather than a strictly scientific development.

The keys provided in this work are traditional and based on the most essential diagnostic characters, mostly following the Russian tradition (Schljakov 1989, Sennikov 2000, Sennikov 2003b), which is ultimately based on the old Finnish revisions (Norrlin 1906). However, we recognise the natural difficulties in application of dichotomous identification keys to polymorphic groups with difficult-to-grasp morphological features; in the future, multi-entry keys should be developed instead.

The present checklist includes 137 accepted species, of which 136 are deemed constantly apomictic. The only sexual (and also facultatively apomictic) taxon at the northern latitudes in the Northern Hemisphere, *H.umbellatum* (Baldesi et al. 2025), was confirmed as diploid and triploid in Finland (Jalas and Pellinen 1985); although its sexual status has not been directly assessed in the country, it can be safely inferred from the ploidy level (Mráz and Zdvořák 2018). For this reason, a different taxonomic approach is applied to this taxon and no "microspecies" are recognised in H.sect.Hieracioides Dumort. (syn. H.sect.Umbellata (Fr.) Gremli) (Sennikov 2003c).

Only two apomictic species of *Hieracium* are considered alien in Finland, but much more are expected with a deeper exploration. All these species were introduced over 100 years ago in old parks and manors as ornamental plants (Hylander 1943). Their native Central European distribution areas was postulated by Hylander (1943); so far, the origin remains largely unknown for the majority of such introduced species (Tyler 2004b) with a notable exception of *H.grandidens*, which may have been introduced from Central Europe, for example, the Czech Republic, which belonged to the area of the German-language European park style before the First World War.

As long as detailed distribution maps are available for only a selection of the Finnish *Hieracium* species (Samuelsson 1954, Ranta 1991, Ranta 1994, Ranta 1996, Ranta 2001, Ranta 2015), province-level distribution data may serve for a spatial analysis of species distribution patterns and the delimitation of phytogeographic elements.

According to the distribution pattern of *Hieracium* species in Finland and their phytogeographic connections, three major areas and their corresponding floristic elements can be delimited: the western sector (mostly Åland Islands with the strong phytogeographic connections to Sweden), the eastern sector (mainland Finland with the distribution areas extending mostly eastwards) and the northern zone (Lapland with its southern extensions).

The analysed distributions generally follow the same patterns as in other vascular plants of Finland (Lahti et al. 1988), i.e. with a prominent presence of southern (forest) and northern (tundra) types, as well as oceanic and continental elements. The only one, but remarkable exception from the common patterns is the species set of the Åland Islands that clearly belongs to the Swedish flora, but has not much in common with the neighbouring Finnish mainland, in which the western connections are much weaker. This anomaly should be explained by separate in situ hybridogenous speciation events in the territories of southern Scandinavia and southern Finland, which have produced pools of hybrid taxa with regionally restricted distributions and by the subsequent colonisation of the islands from Sweden with the lack of dispersal from the south Finnish gene pool. A phytogeographic connection between southern Sweden and Åland Islands has been found also by Tyler (2000b).

As the Åland Islands emerged from the sea in the Holocene and have never been connected with the mainland (Björck 1995), the *Hieracium* species had to colonise their territory across the waters (38 km) separating the islands from Roslagen on the Swedish mainland. The seeds cannot be transported by water and their winter drift is unlikely because of the early dispersal; human-aided transportation can be excluded although the *Hieracium* species were deemed largely hemerophilous by some researchers (Ahti and Hämet-Ahti 1971). Ornithochory (perhaps implying active dispersals by Fringillidae) is, therefore, suggested as the main pathway that presumably formed regional distribution areas of many *Hieracium* species; similarly, it can be invoked to explain the dispersal of many "Swedish" *Hieracium* species to Saaremaa Island, which occur there in the original isolation from the mainland (Sennikov 2003b). Birds appear to act as regional dispersal agents in various plant groups (e.g. shaping the secondary area of *Bidensfrondosa*: Sennikov and Lazkov (2022)) and their role in forming plant distribution areas in *Hieracium* should be more closely examined.

Another group of species with the western phytogeographic connections shows a strictly oceanic pattern of their geographical distribution in Finland. These species mostly belong to the Oreadea group and occur rather narrowly restricted to the south-western coastlines. Their exceptional presence on lakeside rocks in the mainland (Tavastia australis: *H.crinellum*, *H.subonosmoides*) is undoubtedly a relic of their postglacial distribution along the coasts of the former Ancylus Lake in the Holocene (Björck 1995, Tikkanen 2006).

The eastern sector constitutes the specifics of the taxonomic diversity of *Hieracium* in Finland and may represent the result of the autochthonous development of the primary genetic pool that was established during the colonisation of Northern Europe by *Hieracium* plants in the postglacial period. It harbours the species that can be characterised by more southern (and oceanic) and more northern (and continental) distributions.

In this context, highly remarkable is the presence of several apomictic *Hieracium* species which are either endemics or near-endemics of Finland. While this phenomenon can be suspected in the poorly-explored Finnish north, it can be claimed with certainly in the south, where the eastern species distribution limits have been already established (Schljakov 1989, Sennikov 2000, Sennikov 2003b). A large number of taxa is specific to southern or central Finland; their distributions do not extend to southern Sweden and even the Åland Islands, but may slightly continue eastwards to Lake Ladoga or Lake Onega within the phytogeographic Eastern Fennoscandia (Hiitonen 1962). These species with regionally restricted distributions imply the existence of the south-Finnish *Hieracium* speciation centre, whose time period and genetic (or phytogeographic) connections remain unexplored.

The northern floristic area is not so specific in Finland because of a minor portion of Lapland within the national borders. Its actual diversity and distribution limits remain insufficiently studied.

The species number of the apomictic *Hieracium* species currently revealed in Finland is far from final. Some taxonomic groups (Atrata, Nigrescentia, Oreadea, Rigida) are clearly underexplored and their much higher taxonomic diversity is expected. Similarly, the southern territories are relatively well known, but the *Hieracium* exploration of northern Finland has been only rudimentary. It can be expected that at least 200 native species of *Hieracium* occur in Finland. Although this figure is still much lower than the corresponding taxonomic diversity in Sweden (e.g. Tyler (2017)), the difference seems to be naturally grounded on the closer proximity of Sweden to the Scandinavian Mountains, which are the main centre of the postglacial speciation of *Hieracium* in Northern Europe.

## Supplementary Material

99469F8E-0E94-5B0F-BE02-E68C51F23054 10.3897/BDJ.13.e154676.suppl1 Supplementary material 1 Province-level occurrence dataset of *Hieracium* species in Finland Data type occurrences Brief description This dataset contains occurrence data for the currently recognised species of *Hieracium* s. str. in Finland. The species occurrences are based on herbarium collections kept at the Botanical Museum, University of Helsinki and are detailed to the level of biogeographic province. Species abundance (three-step gradation) is estimated for each record on the basis of the number of herbarium specimens collected. Residence status (native vs. introduced) is also indicated for each record. The province names and abbrevations used in the dataset are explained in Fig. 1. File: oo_1290710.csv https://binary.pensoft.net/file/1290710 Sennikov, A.N.

## Figures and Tables

**Figure 1a.**
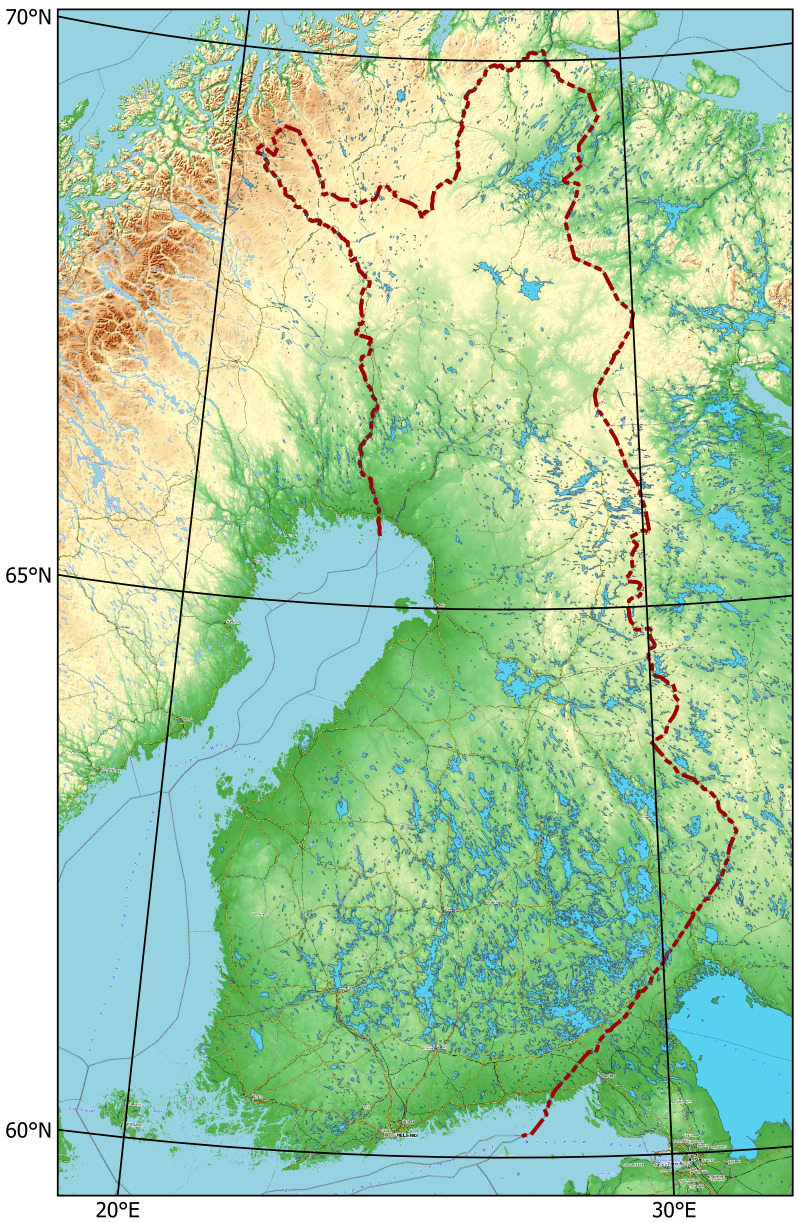
Orographic map of Finland (country land borders as a red line). Colour codes for elevations: green (0-200 m), yellow (200-400 m), brown (400-1400 m). Base map data: © OpenStreetMap contributors. Base map rendering: © OpenTopoMap (CC-BY-SA).

**Figure 1b.**
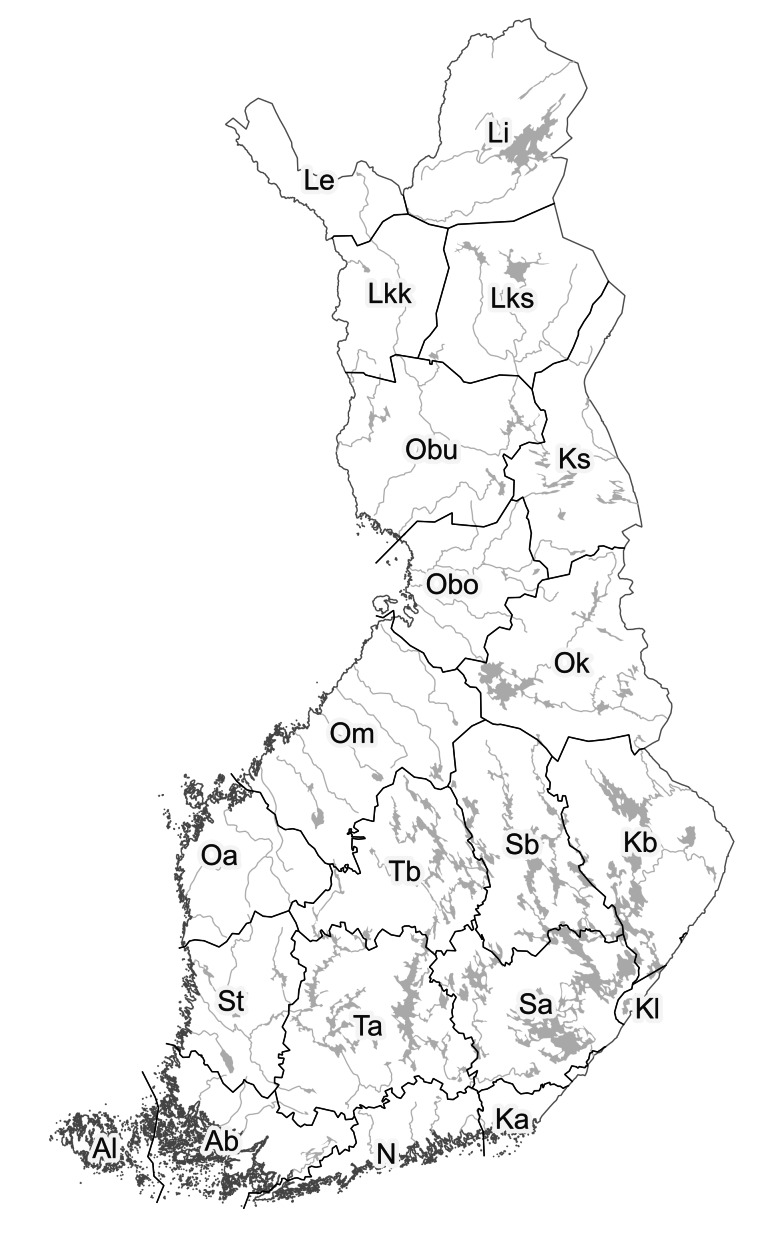
Traditional biogeographic provinces of Finland. Province codes and names in Latin, in their linear sequence (west to east, then south to north): Al (Alandia), Ab (Regio aboënsis), N (Nylandia), Ka (Kareliaaustralis), St (Satakunta), Ta (Tavastia australis), Sa (Savonia australis), Kl (Karelialadogensis), Oa (Ostrobottnia australis), Tb (Tavastia borealis), Sb (Savonia borealis), Kb (Karelia borealis), Om (Ostrobottnia media), Ok (Ostrobottnia kajanensis), Obo (Ostrobottnia borealis), Obu (Ostrobottnia ultima), Ks (Regio kuusamoënsis), Lkk (Lapponia kittilensis), Lks (Lapponia sompiensis), Le (Lapponia enontekiensis), Li (Lapponia inarensis). Source: Heikinheimo and Raatikainen (1981).

**Figure 1c.**
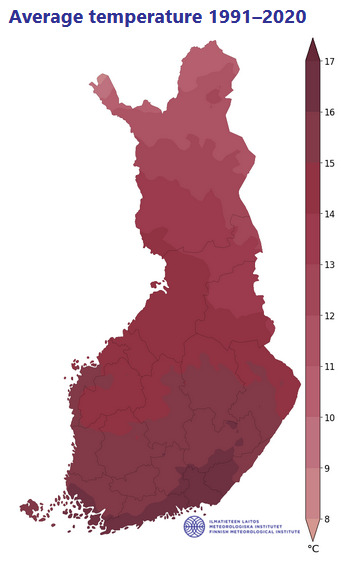
Average summer temperatures in Finland for 1991-2020. Source: Finnish Meteorological Institute (https://en.ilmatieteenlaitos.fi/). Image distributed under the Creative Commons Attribution 4.0 International licence (CC BY 4.0).

**Figure 1d.**
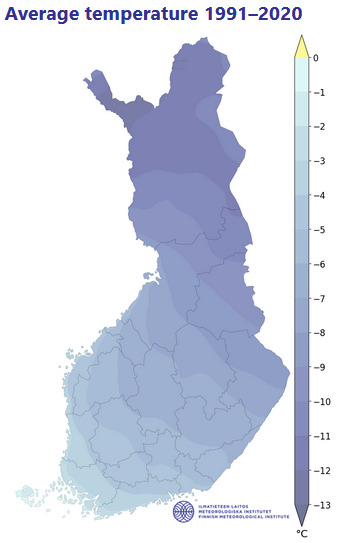
Average winter temperatures in Finland for 1991-2020. Source: Finnish Meteorological Institute (https://en.ilmatieteenlaitos.fi/). Image distributed under the Creative Commons Attribution 4.0 International licence (CC BY 4.0).

**Figure 2a.**
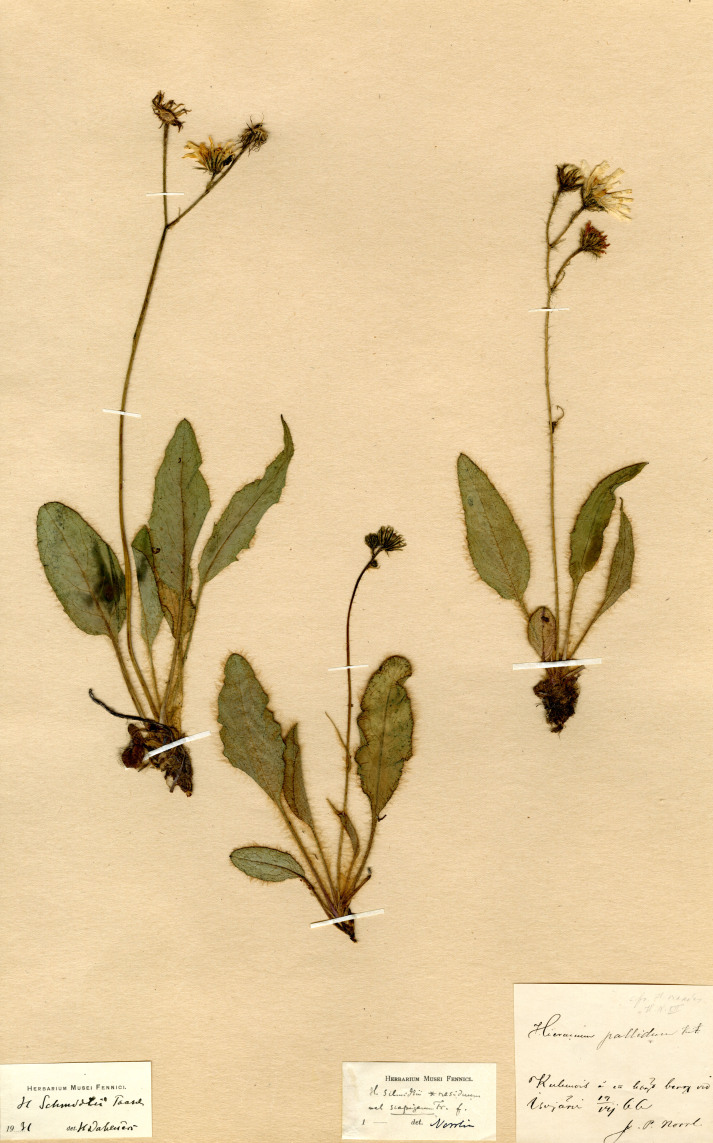
Lectotype specimen. Finland. Tavastia australis: Kuhmois å en högt berg vid Isojärvi, 19.07.1866, *J.P. Norrlin* (H).

**Figure 2b.**
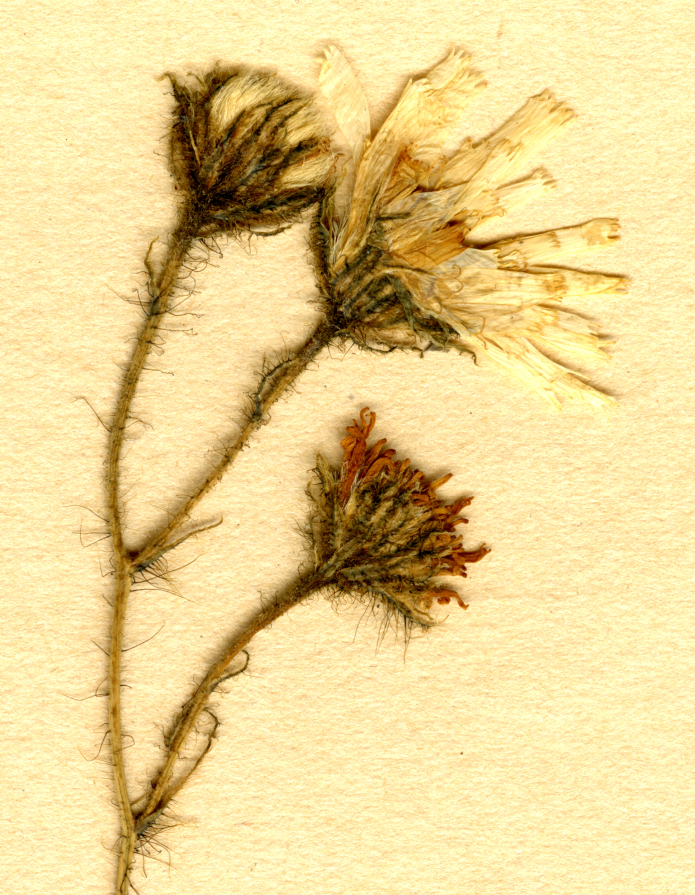
Details of the synflorescence.

**Figure 3a.**
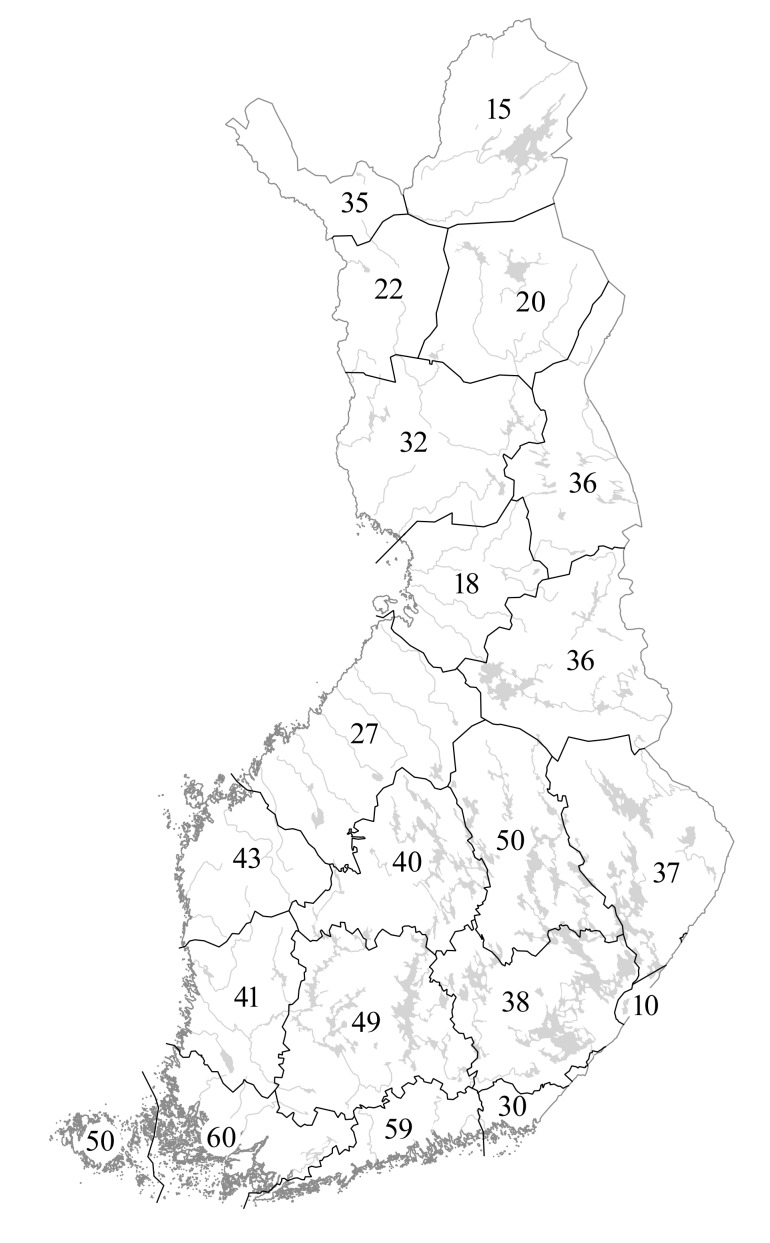
Species number;

**Figure 3b.**
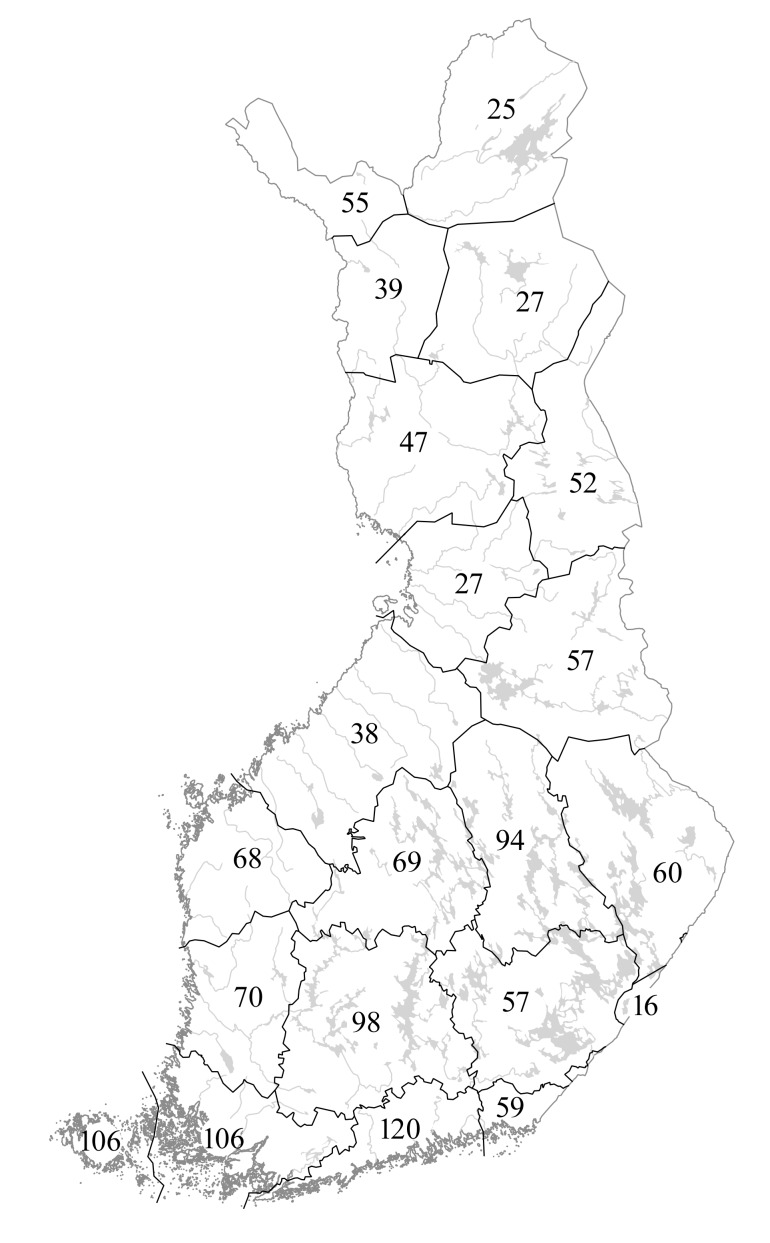
Cumulative species abundance (three-step graded).
